# The effects of abiotic factors in South African semi-arid grassland communities on *Seriphium plumosum* L density and canopy size

**DOI:** 10.1371/journal.pone.0202809

**Published:** 2018-08-30

**Authors:** Hosia T. Pule, Julius T. Tjelele, Michelle J. Tedder

**Affiliations:** 1 Agricultural Research Council, Animal Production Institute, Irene, South Africa; 2 School of Life Sciences, College of Agriculture, Engineering and Science, University of Kwa-Zulu Natal, Scottsville, South Africa; Estacion Experimental del Zaidin, SPAIN

## Abstract

Most studies suggest that multiple factors are responsible for woody plant encroachment, but are inconclusive on its causes. Woody plant encroachment is modified by local patterns of disturbance, topography, soil texture and fertility, and their relative importance and interaction strength varies among locations. We used grassland communities, Carletonville Dolomite Grassland (CDG), Rand Highveld Grassland (RHG) and abiotic factors (Soil Organic Carbon (SOC), Total nitrogen (TN), Phosphorus (P), Potassium (K), Sodium (Na), Calcium (Ca), Magnesium (Mg) and pH) to investigate habitat preferences of *Seriphium plumosum* L. in South African semi-arid grassland. Grassland community had a significant effect on *S*. *plumosum* density, canopy size, and on components of soil fertility (P< 0.05). Slope position had a significant effect on *S*. *plumosum* density and canopy size (P< 0.05). *Seriphium plumosum* density was significantly lower (23 plants/50 m^2^±2.39 (SE)) in CDG than in RHG (40 plants/50 m^2^±4.19). By contrast, *S*. *plumosum* canopy size was significantly higher (1.39 m^2^±0.11) at CDG than at the RHG (1.06 m^2^±0.08). The interaction of grassland community and slope position had a significant effect on *S*. *plumosum* density and soil sodium content (P< 0.05). Bottom slope regions in RHG (59.00 plants/50 m^2^±8.62) had higher *S*. *plumosum* density than bottom slope regions (19.75 plants/50 m^2^±3.01) in CDG. Soil sodium content was similar in CDG slopes and higher than in RHG slopes. *Seriphium plumosum* density was positively related to components of soil fertility; P (*r*^*2*^ = 0.1270; P 0.0036), K (*r*^*2*^ = 0.0786; P 0.0237), Na (*r*^*2*^ = 0.0686; P 0.0350), Ca (*r*^*2*^ = 0.0681; P 0.0358), and SOC content (*r*^*2*^ = 0.0669; P 0.0374). However, *Seriphium plumosum* canopy size did not show any relationship with components of soil fertility. This study revealed that *S*. *plumosum* density in grassland communities increased with increasing soil texture and fertility.

## Introduction

Woody plant encroachment is an increase in density, cover and biomass of trees or shrubs, especially in the arid and semi-arid grassland and savanna biomes [1]. Causes of woody plant encroachment have been widely documented [2, 3, 4, 5], however the problem persists. This is probably because the interaction of different factors such as species adaptations, land-use history, and climate trends alone or in combination may lead to woody plant encroachment [6]. Woody plant encroachment is associated with decline in forage productivity [7], species richness [8], biodiversity [9] and increased erosion [10]. Consequently, it decreases pastoral productivity, particularly in dryland ecosystems [11] where grazing by cattle and sheep is the primary land use [12, 6]. Understanding causes of woody plant encroachment is important because arid and semi-arid lands or dry lands cover about 41% of the terrestrial surface of the earth with approximately 2.4 billion people living in these habitats [13].

Woody plant encroachment has global and local drivers. The global drivers includes climate change and increased atmospheric carbon dioxide [6]. The prime causes of woody plant encroachment at local scale are heavy grazing pressure, animal seed dispersal [14], fire suppression [9], and their interactions [2]. Grazing pressure reduces fuel load and fire frequency, which may contribute to increasing woody plant dominance [2]. This is consistent with the overgrazing hypothesis, which proposes that sustained heavy grazing reduces above- and below-ground grass biomass, thus increasing resource availability for the establishment and recruitment of shrubs [7]. However, woody plant encroachment is also widespread in areas where grazing is infrequent and light [14, 15]. Woody plants seeds that passes through digestive tracks of browser also have a potential to germinate and recruit, thus facilitating woody plant encroachment [16]. Elevated atmospheric carbon dioxide [17, 18] and increased temperatures [19] caused by increasing industrialization [20] may also contribute to cause woody plant encroachment [21].

Soil properties (texture, depth and fertility) and topography interact to influence the pattern of woody plant abundance and distribution [22]. The extent to which portions of a landscape may differentially capture or retain scarce water and nutrient resources (e.g. top to the bottom of the slope) is an important determinant of vegetation patterns, particularly with respect to the distribution of woody plants [23]. Woodlands occur on coarse-textured soils and savannas on fine-textured soils [24]. The two-layer soil water hypothesis of tree-grass coexistence [25] posits that trees and grasses differ in rooting depth, with grasses exploiting soil water in shallow layers, while trees have exclusive access to deep water [26, 25, 27]. Soil texture regulates the infiltration and percolation of rainwater, suggesting that the deep sandy soils often on bottom slopes are more suitable for woody or shrubby plant encroachment compared to the shallow, fine textured soils on top slopes, which are dominated by grasses [28]. The rainfall dependence of dry savanna and grasslands obscures the importance of edaphic factors in determining community structure and functioning [29]. In areas where climate and soils are capable of supporting increased woody plant densities, the occurrence of periodic fires or higher densities of browsers utilizing woody plants can prevent woody plants from encroaching [6]. Generally, there is a complex interaction of local-scale factors (soil properties and topography) with grazing pressure and browsers through dispersing viable seeds that contribute to causing woody plant encroachment [14]. However, most of these factors are studied individually [15]. Consequently, this study addresses how complex interaction of these local factors and other drivers may contribute to the development of management strategies for woody plant encroachment in rangeland communities.

The study also aims to clarify the effect of *S*. *plumosum* encroachment on soil fertility. This is because woody or shrubby plant encroachment can also create “islands of fertility”, by increasing nutrient availability below the shrubs, thus providing habitat for species that are sensitive to low nutrient availability [30]. Nutrient concentration and organic matter are positively and negatively associated with woody or shrubby plant canopy cover and interspaces, respectively [2]. In South African semi-arid savannas, the soil nutrient status (Total Nitrogen (TN), Soil Organic Carbon (SOC), and Calcium (Ca)) under woody plant canopies was higher than between canopy areas [31]. Thus, woody plant encroachment may obstruct surface flows of water and wind, thereby capturing, concentrating and conserving water and nutrient runoff [31]. Although in doing so, woody plant encroachment may mitigate disturbances caused by intense grazing and high fire intensities [32], thus enhancing woody plants persistence and development more than grasslands [33]. However, little research exist on how *S*. *plumosum* encroachment affects soil fertility, yet important to understand its general role on ecosystems.

Generally, the outcomes of woody plant encroachment in semi-arid grassland and savanna communities are not universal [34]. There is little consensus on the consequences of woody plant encroachment for soil nutrient enrichment [35, 36, 37]. This is because the outcomes of woody plant encroachment depend on soil properties, topographic position [38], rainfall [39], plant functional traits and their interactions [38]. Understanding woody plant encroachment is critical because grassland and savanna communities are undergoing widespread degradation [39] caused by among others, reduction in grass productivity [12].

In South Africa, *Seriphium plumosum* L., an indigenous, unpalatable shrub in the Asteraceae family, has increased in abundance within its historic range in the fynbos biome and expanded its geographic range in to the grassland biome [40]. *Seriphium plumosum* encroachment has converted extensive grazing areas into less productive rangelands [41], through displacing palatable grass species [42], and reducing carrying capacities of grazing lands. Control methods, including chemical and mechanical options have failed to control *S*. *plumosum* encroachment. Consequently, solutions that integrate knowledge of the ecology of *S*. *plumosum* species, such as soil—water relationships in rangelands communities, will probably improve our ability to control its encroachment in grasslands.

This study is aimed at determining; 1) the effect of grassland communities, slope position (top, mid and bottom) and their interactions on *S*. *plumosum* density, cover and soil fertility (Total nitrogen (TN), Phosphorus (P), Potassium (K), Sodium (Na), Calcium (Ca), Magnesium (Mg) and Soil Organic Carbon (SOC) and pH). 2) The type of association found between *S*. *plumosum* densities, cover, and soil fertility. We predicted that 1) *Seriphium plumosum* density, size and soil fertility will be high at bottom slopes due to run-on of nutrients and rain water. 2) *Seriphium plumosum* density will vary between grassland communities, because of local differences in environmental factors and disturbance regimes, among others.

## Materials and methods

### Study area

The study was conducted on Kaalfontien and Schietfontein private owned farms situated in Gauteng, South Africa. The farm owners gave us permission to do field work on their farms and there was no specific permission required for each locations, since both farms were private owned and the study did not involve any endangered or protected species. These farms are located in Carletonville Dolomite Grassland (Gh15) and Rand Highveld Grassland (Gm 11) veld types, respectively [43]. Kaalfontein farm is situated approximately 40 km, west of Johannesburg (27.46738° E, 26.08041° S). Schietfontein farm is situated approximately 40 km east of Pretoria (28.67918° E, 25.76907° S). In both veld types *S*. *plumosum* coexisted with a diversity of herbs, many of which also belong to Asteraceae family. *Seriphium plumosum* occurs in predominantly disturbed and overgrazed areas [42, 44] of the Eastern Cape, Free State, Mpumalanga, North West and Gauteng province [44] of South Africa. It also occur in Africa [45], Madagascar and in the USA [46]. Both farms used in this study had a history of livestock grazing with accidental fire occurring approximately every second year. These farms are also encroached by the unpalatable, fynbos shrub, *Seriphium plumosum*.

Generally, the sites on Carletonville Dolomite Grassland (CDG) are higher (mean 1614.28 m ± 0.78 (SE)) in altitude compared to the Rand Highveld Grassland (RHG) sites (1457.24 m ± 0.85). Similarly, soils in the CDG had higher silt (8.66% ± 0.96) and clay (28% ± 0.29) than silt (2.66% ± 0.96) and clay (11% ± 0.20) in RHG. Rain in both veld types where the farms are located falls almost exclusively in summer (October- April), with means of 593 mm and 654 mm per annum for CDG and RHG, respectively [43]. However, during the experiment (2015), the mean annual rainfall of 803 mm and 461.8 mm for CDG and RHG was recorded, respectively. The minimum and maximum summer and winter temperatures for CDG are 11.44 °C and 26.43 °C and in RHG are 9.99 °C and 27.62 °C [43], respectively. However, during the experiment, the mean monthly minimum and maximum summer and winter temperatures for CDG was 10.06 °C and 24.88 °C and in RHG was 7.00 °C and 25.80 °C [S1 Fig], respectively.

Carletonville Dolomite Grassland has a complex mosaic pattern of grasses such as *Aristida congesta*, *Brachiaria serrata*, *Eragrostis chloromelas* and *Alloteropsis semialata*. The soils are mostly from the Dolerite and chert of the Malmani Subgroup, which support mostly shallow Mispah and Glenrosa soil forms [43]. The RHG is species-rich, with wiry, sour grassland alternating with low, sour shrublands on rocky outcrops and steep slopes. The most common species belong to the genera; *Themeda*, *Eragrostis*, *Heteropogon* and *Elionurus*. The soils are from the Quartzite ridges of the Witwatersrand Super group, which support soils of various quality such as Glenrosa and Mispah, along rocky ridges [43].

### Sampling design

A factorial design consisting of two grassland communities (CDG and RHG) x three slope positions (bottom, mid and top slopes) with twelve replicates. The sampling sites at each grassland community had six replicates and two subplots at each of the three slope positions (top, mid and bottom) on the north-facing slopes. The two subplots at each slope position were chosen randomly from four previously established subplots. There were 10 m buffer zones between subplots and slope positions. The distance (measured from the center of each subplot) between the two subplots used in this study ranged between 35 m (i.e. for adjacent subplots) and 75 m (i.e. for nonadjacent subplots) apart within slope positions and 35 m (i.e. for adjacent subplots) and 130 m (i.e. nonadjacent subplots) between slope positions (Table 1).

**Table 1:** Schematic representation of a replicate used at each of the grassland community.

Slope position	1	2	3	4
**Top**	**X**	**X**		
**Mid**		**X**		**X**
**Bottom**	**X**		**X**	

**X** represents randomly selected subplots within and between slope positions at each of the six replicates and grassland community.

There were 50 m buffer zones between the replicates at each of the grassland communities. The centers of the replicates were approximately 150 m apart from each other. The variables measured included *S*. *plumosum* density and canopy size, and components of soil fertility: Soil organic carbon (SOC), Total Nitrogen (TN), Phosphorus (P), Potassium (K), Sodium (Na), Calcium (Ca), Magnesium (Mg) and pH.

### Vegetation and soil sampling

*Seriphium plumosum* density, canopy size and soil samples were collected from March to June 2015 at both veld type-sites. *Seriphium plumosum* density was determined by counting all individuals rooted in a 50 m^2^ (25 m x 2 m) belt-transect, running downhill, at the center of each 625 m^2^ (i.e. 25 m x 25 m) subplots. Density was expressed as mean individual plants per unit area (50 m^2^). Canopy size was determined by measuring horizontal diameter (to the nearest 2 cm) of the long and short axis of the canopy of individuals whose canopy intercepted the 25 m line transect placed down slope in each of the subplots and slope positions. Canopy size was calculated using an ellipse function (C = *abπ* / 4.0), where a represents the long axis and b represents the short axis of the canopy [47] and expressed as mean individual canopy size per unit area (m^2^). Since *S*. *plumosum* requires water and nutrients to germinate, recruit and survive, but reported to dislike fertile soils [41], the soil samples were taken from 5 cm depth and five random points, irrespective of their proximity to (i.e. below or between) *S*. *plumosum* plants in each belt transect. The soils were analyzed for eight components of fertility using Ambic I extraction followed by AA spectroscopy. The sub-sample of soils from each of the two subplots at each slope position in each replicate were pooled together and analysed for texture (silt and clay) at the Agricultural Research Council-Institute for Soil, Climate and Water (ARC-ISCW) soil analyses accredited laboratory.

### Statistical analyses

*Seriphium plumosum* density, canopy size and soil fertility at two grassland communities and three slope positions was analysed as a completely randomised design with a 2 × 3 factorial analysis of variance (ANOVA) using GLM procedures. Data for *S*. *plumosum* density, size and soil fertility for each slope position were averaged before the analysis. Data met the assumption of ANOVA concerning homogeneity of variance before being analysed for the effect of grassland community, slope position and their interaction on *S*. *plumosum* density, canopy size, and components of soil fertility using SAS [48]. When the ANOVA produced significant results, the effect of grassland community, slope position, their interactions on *S*. *plumosum* density, cover, and components of soil fertility were compared using the Turkey’s HSD test, and the differences declared significant at P < 0.05. Using linear regression analyses in SAS [48], we explored the relationship between *S*. *plumosum* density, individual size and the eight components of soil fertility.

## Results

### Vegetation parameters

The results on the main factors of grassland community and slope position, as well as their interaction on *S*. *plumosum* density and canopy size are presented in table below (Table 2).

**Table 2:** *F* values and *P* values for the effects of grassland community and slope position and their interaction on *Seriphium plumosum* L density and canopy size.

		Vegetation parameters			
Factors		*S*. *plumosum* density		*S*. *plumosum* canopy size	
	DF	F	P	F	P
Grassland community	1	16.11	**0.0002**	5.83	**0.0186**
Slope position	2	3.97	**0.0236**	3.67	**0.0307**
Grassland community x slope position	2	7.08	**0.0016**	0.05	0.9521

Significant values are shown in bold.

**Fig 1:**
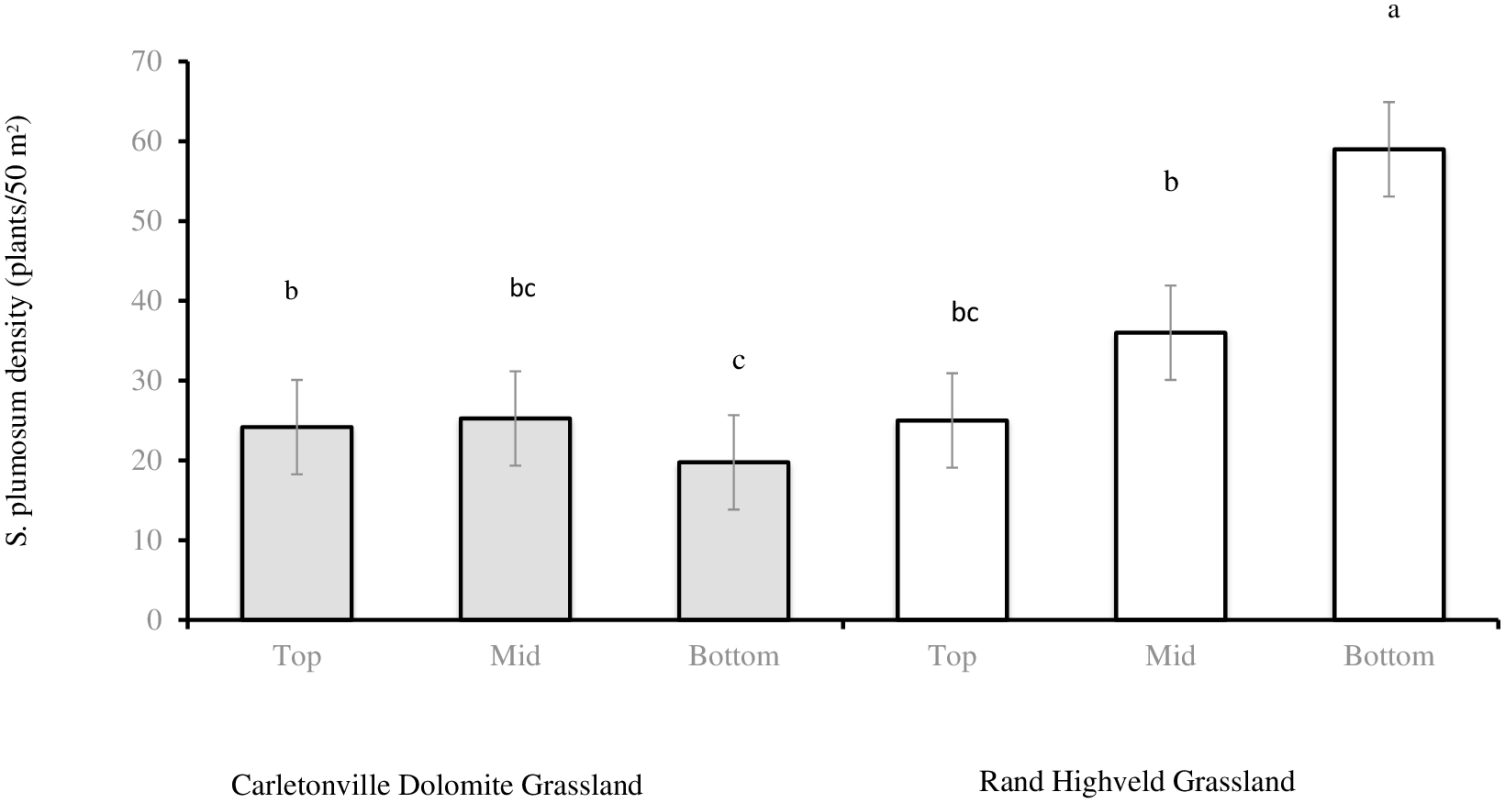
*Seriphium plumosum* density at CDG (n = 36) and RHG (n = 36) slopes sites. Bars represent standard error (SE). Same letters on the bars mean that P > 0.0500.

*Seriphium plumosum* density was significantly lower in CDG (23 plants/50 m^2^ ± 2.39) than in the RHG (40 plants/50 m^2^ ± 4.19). In general, *Seriphium plumosum* density at bottom slopes (39 plants/50 m^2^ ± 6.06) was significantly higher than at the top slopes (25 plants/50 m^2^ ± 2.88). *Seriphium plumosum* density at the mid slopes (31 plants/50 m^2^ ± 3.61) was not significantly different from the bottom and top slopes. Grassland community x slope position had a significant interaction effect on *S*. *plumosum* density (P < 0. 0016) (Fig 1).

Bottom slope regions in RHG (59.00 plants/50 m^2^ ± 8.62) had significantly higher *S*. *plumosum* density than in the mid slopes (36 plants/50 m^2^ ± 4.88) and top slopes (25 plants/50 m^2^ ± 3.94) as well as all slope positions in the CDG. Canopy size of *S*. *plumosum* was larger in CDG (1.39 m^2^ ± 0.11) than in RHG (1.06 m^2^ ± 0.09). In general, *S*. *plumosum* individual canopy size were bigger at the top slopes (1.42 m^2^ ± 0.13) than at the bottom slopes (0.99 m^2^ ± 0.12), while canopy size at the mid slopes (1.29 m^2^ ± 0.11) was similar to both top and bottom slopes.

### Components of soil fertility

Grassland community differed significantly in soil organic carbon (P < 0.0001), total nitrogen (P < 0.0001), phosphorus (P < 0.0001), potassium (P < 0.0001), sodium (P < 0.0001), calcium (P < 0.0001) and magnesium (P < 0.0001) content. However, these differences were not observed for soil pH content (Table 3).

**Table 3:** *F* values and *P* values for the effects of grassland community and slope position and their interaction on components of soil fertility.

Factors	Components of soil fertility																
		SOC		TN		P		K		Na		Ca		pH		Mg	
	DF	F	P	F	P	F	P	F	P	F	P	F	P	F	P	F	P
**GC**	1	164.47	**< 0.0001**	36.43	**< 0.0001**	98.19	**< 0.0001**	53.02	**< 0.0001**	80.15	**< 0.0001**	104.70	**< 0.0001**	0.32	0.5759	55.47	**< 0.001**
**SP**	2	1.28	0.2845	1.76	0.1799	1.26	0.2915	0.04	0.9622	2.38	0.1006	0.45	0.6412	0.10	0.9041	0.56	0.5716
**GC X SP**	2	0.84	0.4376	1.54	0.2231	158	0.2145	0.54	0.5854	4.28	**0.0181**	0.11	0.8978	2.90	0.0619	0.80	0.4552

Significant values shown in bold.

GC, Grassland Community; SP, Slope position; SOC, Soil Organic Carbon; TN, Total Nitrogen; P, Phosphorus; K, Potassium; Na, Sodium, Ca; Calcium, Mg; Magnesium.

All the components of soil fertility were significantly higher in CDG than in RHG, except pH that was similar at both grassland communities (Table 4).

**Table 4:** Components of soil fertility in Carletonville Dolomite Grassland (CDG) and Rand Highveld Grassland (RHG).

	CDG			RHG		
Components of soil fertility	N	Mean	SE	N	Mean	SE
SOC (%)	36	1.52	0.06	36	0.77	0.03
TN (%)	36	0.05	0.00	35	0.00	0.00
P (%)	36	4.14	0.26	36	1.13	0.16
K (mg/kg)	36	115.04	7.09	36	52.71	4.47
Ca (mg/kg)	35	136.97	11.62	36	15.71	1.06
Mg (mg/kg)	34	49.14	4.25	36	8.39	4.41
pH (mg/kg)	36	5.0	0.04	36	9.90	2.22
Na (mg/kg)	33	3.79	0.30	35	0.79	0.20

**Fig 2:**
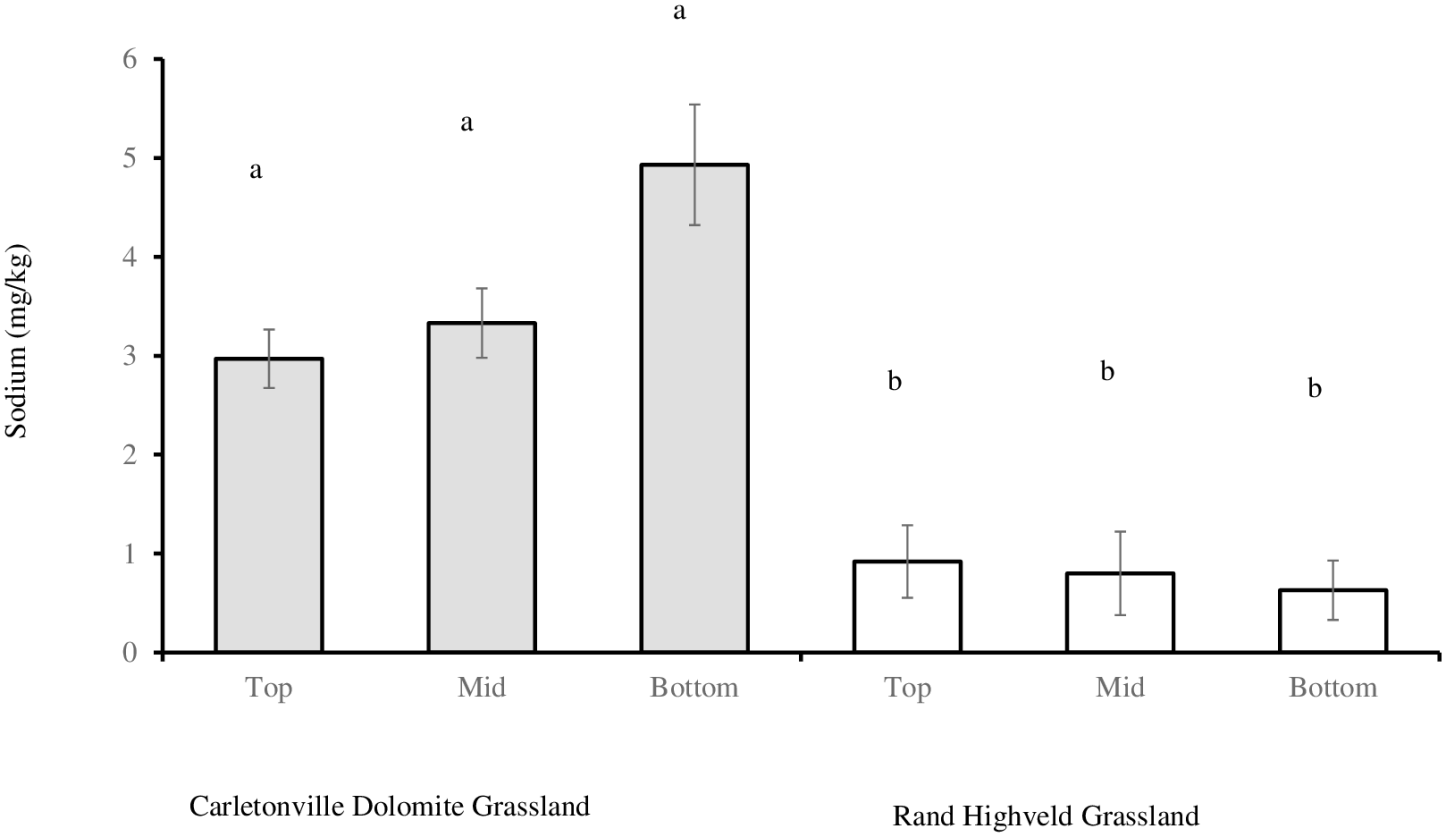
Soil sodium content at CDG (n = 33) and RHG (n = 35) slopes sites. Bars represent standard error (SE). Same letters on the bars indicate that P > 0.05.

No effect of slope position in any soil fertility components was found (P = 0.1006). The interaction of grassland community and slope position had significant effect on soil sodium content (P = 0.0181). Soil sodium content was the same at all slope positions in Carletonville Dolomite Grassland and significantly higher than similar slopes in Rand Highveld Grassland (Fig 2).

The density of *S*. *plumosum* increased with soil fertility. The effect was weak but significant (P < 0.05). Canopy size did not show any significant relationship with the components of soil fertility (P > 0.05; Table 5).

**Table 5:** Regression analysis for the relationship between *Seriphium plumosum* density, canopy size and components of soil fertility; P < 0.05 significance level.

Components of Soil fertility	*S*. *plumosum* density		*S*. *plumosum* canopy size	
	*R*^*2*^	*P*	*R*^*2*^	*P*
Soil organic carbon (%)	0.0669	**0.0374**	0.0302	0.1660
Total Nitrogen (%)	0.0112	0.4010	0.0009	0.8170
Phosphorus (mg/kg)	0.1270	**0.0036**	0.0207	0.2530
Potassium (mg/kg)	0.0786	**0.0237**	0.0158	0.3182
Sodium (mg/kg)	0.0686	**0.0350**	0.0153	0.3270
Calcium (mg/kg)	0.0681	**0.0358**	0.0398	0.1112
Magnesium (mg/kg)	0.0548	0.0605	0.0324	0.1513
pH (H_2_0)	0.0390	0.1149	0.0125	0.3762

## Discussion

*Seriphium plumosum* density was significantly higher at the RHG than in the CDG. This difference between grassland communities suggests that variation in the rate and extent of encroachment is mediated by, among others, the differences in environmental factors among locations [6, 49]. The RHG bottom slopes had higher *S*. *plumosum* density than the top slopes. This supports our prediction that, *S*. *plumosum* density will be high at bottom slopes due to run-on of nutrients and rain water. Ben-Shahar [50] also found that bottom slopes with high woody vegetation density had high soil nutrient content. In addition, Ludwig et al. [51] proposed a decrease in ratio of open areas, free from woody plants to run-on areas of woody plants with increasing rainfall, particularly at the bottom slopes. Woody or shrubby plant encroachment in dryland ecosystems is influenced by, among other factors, high rainfall, which may enhance the establishment of woody or shrubby plant seedlings [52]. Consequently, the higher rainfall and nutrients accumulation at bottoms slopes may explains the higher *S*. *plumosum* density at the RHG bottom slopes found in this study.

Contrary to our prediction, the bottom slopes in CDG had significantly lower *S*. *plumosum* density than mid slopes and top slopes. These results are inconsistent with the results found in RHG, where we found a decrease in *S*. *plumosum* density down the slope. These findings in RHG further contradict research finding reporting a decrease in *S*. *plumosum* density down the slope, with no plants occurring on bottom slope areas [53]. Research reported that the top slopes contains on average less water compared to bottom slopes [50]. The fact that *S*. *plumosum* density were lower at the bottom slopes of CDG may also suggests that it is multiple factors responsible for *S*. *plumosum* germination and recruitment and not only water and nutrients deposited at the bottom slopes. Furthermore, it may suggests that the rate of encroachment stage varies between grassland communities, with RHG being encroached recently relative to CDG. This is because the rate of encroachment are high in the early stages of encroachment, and then declines [54] or fluctuate [35] as maximum cover threshold are approached. Since the CDG had relatively higher soil silt (8. 66%) and clay (28%) than silt (2.66%) and clay (11%) content in the RHG also suggests that CDG bottom slopes may be water logged, and *S*. *plumosum* is sensitive to water logging conditions [53]. Although contrary to our results, other research showed a negative correlation between *S*. *plumosum* density and soil nutrients, i.e., lower density of *S*. *plumosum* when the soil nutrients are higher, which resulted in decreased *S*. *plumosum* density at the bottom slope [53]. The inconsistency in *S*. *plumosum* densities between grassland communities and slope positions observed in this study may suggests that no single driving factor may explain woody plant encroachment, but a combination of interacting factors [28]. Furthermore, it may also suggests that woody plants are generally well suited to a broad range of grassland topoedaphic settings [38]. Generally, the results from this study propose that the effects of slope positions on *S*. *plumosum* density are not universal. Although nutrient and water transported to the bottom slopes may facilitate woody plant seeds germination and assist in explaining *S*. *plumosum* density at bottom slopes, this condition alone may not adequately explain its encroachment, but a combinations of multiple interacting factors [1]. Furthermore, because shrubs act as fertility islands, different management actions may be required at different stages of encroachment because of shrub mediate changes in soil fertility.

*Seriphium plumosum* density was inversely related to individual plant canopy size at the bottom and top slopes of both grassland communities. The RHG had significantly higher *S*. *plumosum* density and lower canopy size. These results suggests that there is a tradeoff between density and plant canopy size. The higher neighborhood densities in plants may result in density dependent mortality or compensate by shifting the crown centers away from the trunks or neighbours [55]. This density dependent mortality or shifting the crown away from the trunks or neighbours could also mean that *S*. *plumosum* is sensitive to shading by other co-existing species in the same communities.

The fact that CDG had a higher percentage of soil silt and clay content than soils in the RHG, might explain the fewer *S*. *plumosum* density observed in CDG than in the RHG (Fig 1). Many other differences observed between CDG and RHG observed in this study might result in the differences in density, such that a higher percentage of certain soil textures may not be the only explanation for observed *S*. *plumosum* densities. The difference in rooting depth between woody and herbaceous species suggests that the fine-textured soils should be more resistant to woody plant encroachment compared to the course-textured soils [17]. Previous research [56], predicted an inverse relationship between woody plant abundance and soil clay content. The higher *S*. *plumosum* density observed in relatively sandier soils in this study support this prediction. *Seriphium plumosum* encroachment in the CDG suggests that the species is able to spread in soils with greater than the 24% clay encroachment threshold, provided they are deep and well drained. The high soils silt and clay content found in CDG, suggested that *S*. *plumosum* is among others, sensitive to fine textured soils and probably water logging conditions. Generally, *S*. *plumosum* prefers slightly deeper sandier soils, which are periodically subjected to water stress [53]. These results further suggests that soil texture is a primary factor influencing *S*. *plumosum* density, with slope position being a background factor.

Carletonville Dolomite Grassland had fertile soils and lower *S*. *plumosum* density compared to the marginal soils and higher *S*. *plumosum* density at the RDG. This finding confirm the research reporting *S*. *plumosum* encroachment of mostly soils low in fertility [45] which are withdrawn from cash-crop cultivation. The lower soil sodium content and higher *S*. *plumosum* density on the bottom slope regions of CDG, may suggests that *S*. *plumosum* cannot tolerate high soil sodium content (Fig 2). This finding suggests that sodium chloride application may assist in controlling *S*. *plumosum* spread on rangeland communities [42, 53], and probably increase grass production.

Interestingly, components of soil fertility (SOC, P, K, Na, and Ca) were positively correlated with *S*. *plumosum* density, but not canopy sizes. These result could not explain whether the ‘‘fertility island effect” was caused by woody plant encroachment and not intrinsic condition of the soils. However these results confirm research findings that the rate of encroachment are highest in the early stages of encroachment and decline or fluctuate as maximum cover threshold are reached [6] and/or when nutrients and water are depleted due to intraspecific competition [29]. It is also proposed that, some attributes increased, and others declined as woody plant encroaches [34]. The findings by Snyman [41] that *S*. *plumosum* encroaches first on marginal soils (low in fertility), need further investigation. The fact that, as woody plants encroach, grass productivity declines, causing soil erosion and reducing the amount of soil nutrients [57] may apply in certain circumstances and not others. Some authors reported that, as woody plants encroaches, soil fertility might increase [56, 34], due to patches of woody vegetation obstructing surface flows of water and wind, thereby capturing, concentrating and conserving runoff water and nutrients [30]. This creates “islands of fertility”, which increase nutrient availability below the shrubs. This “islands of fertility” will in turn provide habitat for species that are sensitive to low nutrient availability [28]. The positive relationship between *Seriphium plumosum* density and components of soil fertility, and *S*. *plumosum* relative abundance in bottom slope may suggests the sensitivity of *S*. *plumosum* to low soil nutrient availability. Although this finding confirm our predictions that, *S*. *plumosum* encroachment would be positively correlated with some components of soil fertility, which would facilitate its germination and recruitment, other research have found the opposite. Consequently, it is suggested in this study that the effect of woody plant encroachment are not universal [34]. Furthermore, it may suggests that woody plant traits also determine the causes and outcomes of encroachment [38].

## Future directions

This study shows *S*. *plumosum* encroachment of mostly, fine textured and fertile soils. Previous research found that, *S*. *plumosum* occurs mainly in coarse-textured, nutrient poor soils, often with relatively low sodium content. These results and that of other, confirm the similarity on the occurrence of *S*. *plumosum* on coarse textured soils, but the opposite on the soil nutrient status. The fact that in this study, *S*. *plumosum* density has shown a weak positive correlation with components of soil fertility and negative correlation reported in the other study, suggests that controlled research on manipulation of soil nutrient resources, especially soil organic carbon (SOC), Phosphorus (P) Potassium (K), and Sodium (Na). This could contribute to understanding how *S*. *plumosum* responds to soil fertility. Furthermore, an interaction of abiotic factors at grassland communities found in this study, suggests that *S*. *plumosum* encroachment is influenced by a complex interaction of multiple abiotic factors. Consequently, a better understanding of the complex interaction of these abiotic factors on *S*. *plumosum* encroachment is needed to aid in its control and management on South African grassland communities.

The relationship between the changes in abiotic factors and the extent, rate and the times of *S*. *plumosum* encroachment on South African semi-arid grassland communities is also lacking. Consequently, more research to understand the relationship between changes in abiotic factors and the extent, rate and times of *S*. *plumosum* encroachment is needed to aid in the development of management and control methods for its encroachment in South African semi-arid grassland communities.

## Supporting information

S1 Fig Mean monthly precipitation (a), minimum (Min) and maximum (Max) temperature (b), for June 2014 to June 2015 in CDG and RHG. (JPG)

S1 Appendix (RTF)
