# Supplementary figures and images for: The effects of abiotic factors in South African semi-arid grassland communities on Seriphium plumosum L density and canopy size
Source: PLoS One. 2018 Aug 30;13(8):e0202809. doi: 10.1371/journal.pone.0202809 (PMC6117004; doi:10.1371/journal.pone.0202809)

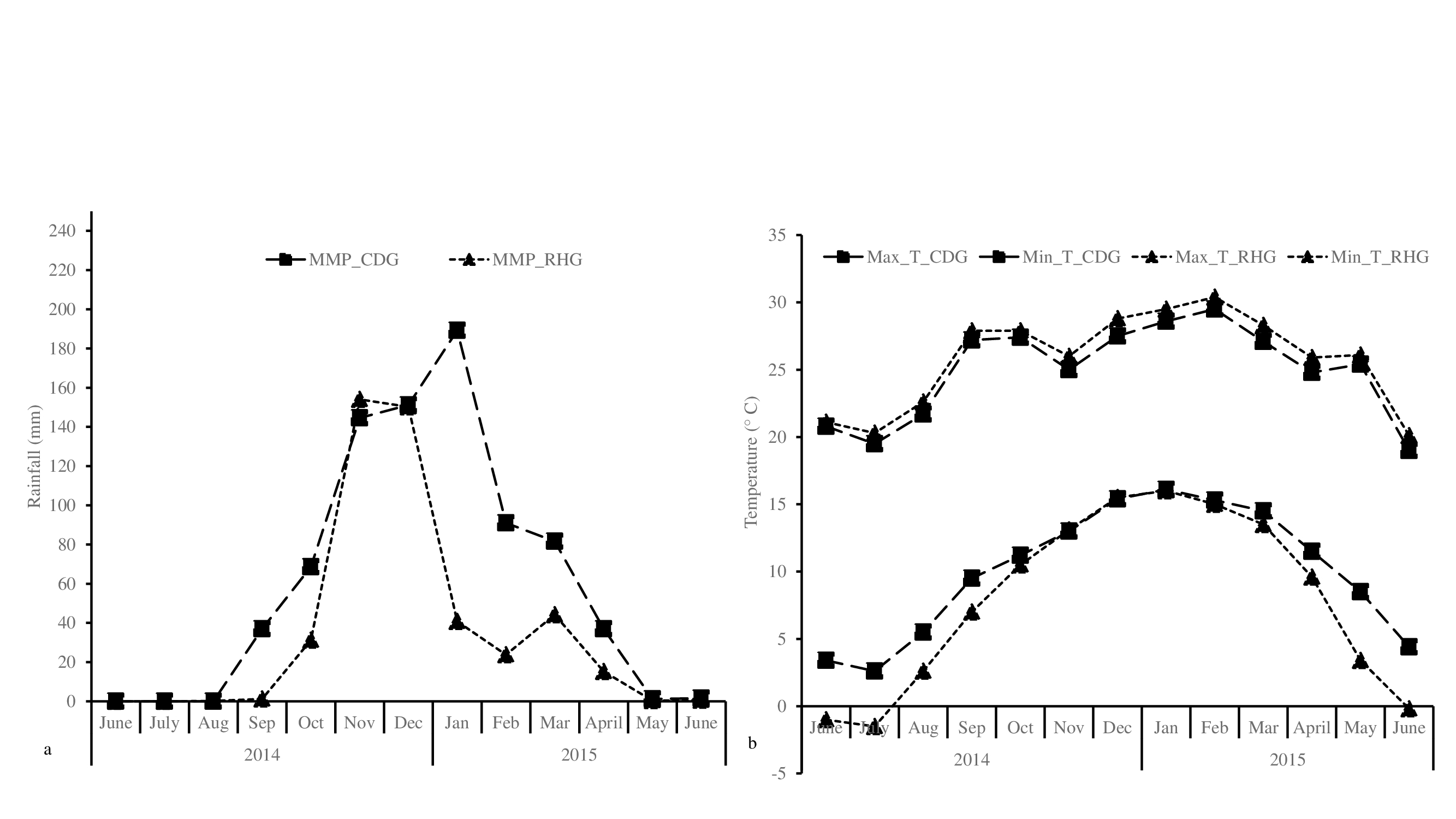

Supplement: S1 Fig — (JPG) [file pone.0202809.s001.jpg]
