# Supplementary material for: The effects of abiotic factors in South African semi-arid grassland communities on Seriphium plumosum L density and canopy size
Source: PLoS One. 2018 Aug 30;13(8):e0202809. doi: 10.1371/journal.pone.0202809 (PMC6117004; doi:10.1371/journal.pone.0202809)
Supplement: S1 Appendix — (RTF) [file pone.0202809.s002.rtf]

  Gilbert Pule: Experiment 2 data for statistical analysisNo Outliers 5cm only.SAS  1
                             Project number = 000971-Y5   08:36 Friday, March 9, 2018

                     G
                     r
                     a
                     s                                                S
                     s                                                p
                     L           S                                    l     I
                     a           l                                    u     n
                     n           o                                    m     d
                     d           p                                    M     i
                     C           e              L               S     e     v
                     o     R     P       A      o        L      p     a     I
                     m     a     o       l      n        a      l     n     n
                     m     i     s       t      g        t      u     C     t
                     u     n     i       i      i        i      m     o     e
           F         n     f     t       t      t        t      D     n     r
  O        a         i     a     i       u      u        u      e     C     c
  b        r         t     l     o       d      d        d      n     o     e
  s        m         y     l     n       e      e        e      s     v     p

  1  Kaalfontein    Gh15  593  Bottom  1615  26.0992  27.4665   25  1.060  11
  2  Kaalfontein    Gh15  593  Bottom  1618  26.0807  27.4671   32  1.140  11
  3  Kaalfontein    Gh15  593  Bottom  1613  26.0814  27.4675   31  0.750  10
  4  Kaalfontein    Gh15  593  Bottom  1613  26.0820  27.4679   32  1.560  13
  5  Kaalfontein    Gh15  593  Bottom  1619  26.0824  27.4680    3  0.000   0
  6  Kaalfontein    Gh15  593  Bottom  1614  26.0868  27.4674   14  0.920   3
  7  Kaalfontein    Gh15  593  Bottom  1614  26.0833  27.4664   23  1.760  11
  8  Kaalfontein    Gh15  593  Bottom  1611  26.0847  27.4661   23  1.820   9
  9  Kaalfontein    Gh15  593  Bottom  1611  26.0828  27.4663   11  1.090   6
 10  Kaalfontein    Gh15  593  Bottom  1612  26.0826  27.4661   23  1.400  14
 11  Kaalfontein    Gh15  593  Bottom  1607  26.0808  27.4650    2  1.050  10
 12  Kaalfontein    Gh15  593  Bottom  1603  26.0815  27.4652   18  1.140  10
 13  Kaalfontein    Gh15  593  Mid     1618  26.0804  27.4674   33  1.060  14
 14  Kaalfontein    Gh15  593  Mid     1618  26.0802  27.4672   36  1.020  16
 15  Kaalfontein    Gh15  593  Mid     1617  26.0818  27.4682   67  0.380  14
 16  Kaalfontein    Gh15  593  Mid     1612  26.0813  27.4678   40  1.180  13
 17  Kaalfontein    Gh15  593  Mid     1609  26.0830  27.4675    8  2.330   2
 18  Kaalfontein    Gh15  593  Mid     1620  26.0826  27.4683    6  1.900   3
 19  Kaalfontein    Gh15  593  Mid     1608  26.0834  27.4669   19  1.670   8
 20  Kaalfontein    Gh15  593  Mid     1622  26.0832  27.4675   18  2.010   8
 21  Kaalfontein    Gh15  593  Mid     1611  26.0822  27.4662   11  1.800   6
 22  Kaalfontein    Gh15  593  Mid     1610  26.0819  27.4659   14  1.420   7
 23  Kaalfontein    Gh15  593  Mid     1608  26.0808  27.4650   26  1.100   7
 24  Kaalfontein    Gh15  593  Mid     1616  26.0818  27.4663   25  1.870  17
 25  Kaalfontein    Gh15  593  Top     1622  26.0899  27.4673   23  1.170  15
 26  Kaalfontein    Gh15  593  Top     1619  26.0804  27.4677   29  1.030  11
 27  Kaalfontein    Gh15  593  Top     1620  26.0809  27.4679   65  0.590  19
 28  Kaalfontein    Gh15  593  Top     1617  26.0814  27.4683   27  0.450   7
 29  Kaalfontein    Gh15  593  Top     1618  26.0830  27.4682   23  1.170  13
 30  Kaalfontein    Gh15  593  Top     1616  26.0832  27.4679   31  1.820  12
 31  Kaalfontein    Gh15  593  Top     1612  26.0834  27.4675   18  2.960  10
 32  Kaalfontein    Gh15  593  Top     1613  26.0836  27.4672    6  3.050   3
 33  Kaalfontein    Gh15  593  Top     1612  26.0826  27.4670   12  2.170   5
 34  Kaalfontein    Gh15  593  Top     1614  26.0817  27.4662    9  1.420   6
 35  Kaalfontein    Gh15  593  Top     1609  26.0809  27.4655   20  1.660   9
 36  Kaalfontein    Gh15  593  Top     1623  26.0811  27.4658   27  1.300  17
 37  Schietfontein  GM11  654  Bottom  1455  25.7685  28.6793  112  0.482  32
 38  Schietfontein  GM11  654  Bottom  1450  25.7687  28.6797   93  0.373  15
 39  Schietfontein  GM11  654  Bottom  1462  25.7687  28.6801   61  0.904  21
 40  Schietfontein  GM11  654  Bottom  1465  25.7688  28.6805    7  2.637   1
 41  Schietfontein  GM11  654  Bottom  1458  25.7689  28.6809   26  0.682  13
 42  Schietfontein  GM11  654  Bottom  1454  25.7689  28.6813   54  0.791  25
 43  Schietfontein  GM11  654  Bottom  1447  25.7690  28.6817   34  0.931  14
 44  Schietfontein  GM11  654  Bottom  1454  25.7691  28.6820   43  0.939  18
 45  Schietfontein  GM11  654  Bottom  1458  25.7692  28.6826   74  1.016  21
 46  Schietfontein  GM11  654  Bottom  1454  25.7692  28.6829   89  0.000   0
 47  Schietfontein  GM11  654  Bottom  1453  25.7696  28.6832   60  0.666  13
 48  Schietfontein  GM11  654  Bottom  1453  25.7697  28.6836   55  0.556  15
 49  Schietfontein  GM11  654  Mid     1461  25.7691  28.6792   75  0.617  27
 50  Schietfontein  GM11  654  Mid     1454  25.7693  28.6795   43  0.986  15
 51  Schietfontein  GM11  654  Mid        .  25.7694  28.6799   45  0.842  18
 52  Schietfontein  GM11  654  Mid     1449  25.7695  28.6806   31  1.456  11
 53  Schietfontein  GM11  654  Mid     1461  25.7695  28.6807   40  1.572  17
 54  Schietfontein  GM11  654  Mid     1461  25.7696  28.6811   25  1.285  13
 55  Schietfontein  GM11  654  Mid     1461  25.7696  28.6815   26  1.887   8
 56  Schietfontein  GM11  654  Mid     1450  25.7697  28.6818   19  1.156  10
 57  Schietfontein  GM11  654  Mid     1464  25.7698  28.6823   15  1.458   9
 58  Schietfontein  GM11  654  Mid     1456  25.7698  28.6826   53  0.000   0
 59  Schietfontein  GM11  654  Mid     1456  25.7699  28.6831   25  0.925  12
 60  Schietfontein  GM11  654  Mid     1460  25.7701  28.6834   46  0.954  16
 61  Schietfontein  GM11  654  Top        .    .        .       35  1.074  18
 62  Schietfontein  GM11  654  Top     1456  25.7699  28.6794   20  1.098   4
 63  Schietfontein  GM11  654  Top     1465  25.7699  28.6798    7  2.069   6
 64  Schietfontein  GM11  654  Top        .    .        .       24  1.177  13
 65  Schietfontein  GM11  654  Top     1462  25.7701  28.6806   15  1.446   7
 66  Schietfontein  GM11  654  Top     1465  25.7702  28.6809   25  1.296  14
 67  Schietfontein  GM11  654  Top     1459  25.7703  28.6814   17  1.161   6
 68  Schietfontein  GM11  654  Top     1453  25.7703  28.6817   19  1.410   4
 69  Schietfontein  GM11  654  Top     1463  25.7704  28.6824   20  1.413  12
 70  Schietfontein  GM11  654  Top     1459  25.7698  28.6826   59  0.826  17
 71  Schietfontein  GM11  654  Top     1454  25.7702  28.6830   20  1.347   4
 72  Schietfontein  GM11  654  Top     1457  25.7703  28.6834   39  1.067  15


       B                                                       S    S
       u                                                       a    i
       l                                                T      n    l
       k                                         O      o      d    t             p
       D                                         r      t      P    P             H
       e                                         g      a      e    e             H
       n                        C        M       _      l      r    r             2
       s        K       N       a        g       C      N      s    s     P       O
       5        5       a       5        5       5      5      5    5     5       5
       D        D       5       D        D       D      D      D    D     D       D
       e        e       D       e        e       e      e      e    e     e       e
  O    p        p       p       p        p       p      p      p    p     p       p
  b    t        t       t       t        t       t      t      t    t     t       t
  s    h        h       h       h        h       h      h      h    h     h       h

  1  1.019   163.49   4.22   139.55    51.52   1.35   0.064   66    8   2.600   4.90
  2  0.999    68.13   7.33    85.47    22.39   1.39   0.064   66    8   5.870   4.56
  3  1.122    59.81   3.64   118.82    30.49   1.54   0.065   66    8   6.370   4.93
  4  0.937   199.84   3.58   277.60      .     1.98   0.084   66    8   4.490   5.16
  5   .      187.27   3.45   218.73    91.72   1.79   0.076   62    8   7.840   5.21
  6  0.995    96.88   3.91   105.43    40.85   1.46   0.062   62    8   3.270   4.69
  7  1.000    55.57   8.70    53.04    18.12   1.38   0.057   62    8   3.260   4.64
  8  1.016    81.71   4.86    81.48    23.35   1.34   0.057   62    8   3.090   4.73
  9  0.986   173.04   3.32   145.50    61.54   1.64   0.074   62   10   4.990   5.06
 10  0.984   123.65   8.53   172.76    68.77   1.70   0.077   62   10   4.500   4.90
 11  1.012    39.01   2.47    37.67    16.06   1.12   0.045   62   10   2.250   4.42
 12  1.037    71.02   5.12    81.99    32.25   1.31   0.061   62   10   3.610   4.69
 13  1.033   104.05   3.41    88.63    32.16   1.36   0.060   66    8   3.110   4.65
 14  1.019   114.45   1.75   120.92    45.06   1.38   0.060   66    8   2.510   4.86
 15  1.043   109.00   2.87   169.26    56.68   1.59   0.070   66    8   3.860   5.05
 16  1.046   129.79   4.78   178.40    67.18   1.49   0.060   66    8   6.220   5.06
 17  0.989   124.05    .     137.48    60.11   1.41   0.058   62    8   3.520   4.72
 18   .      178.64    .     196.97    85.49   1.74   0.075   62    8   4.290   4.89
 19  1.087    87.98   4.13    87.39    29.36   1.25   0.058   62    8   3.010   4.73
 20  1.058    50.41   4.65    42.14    12.84   1.18   0.056   62    8   3.070   4.65
 21  0.968   134.94   4.37   188.84    80.58   1.91   0.087   62   10   3.500   4.89
 22  0.953   123.25   3.03    89.96    36.69   1.52   0.066   62   10   3.940   4.63
 23  1.045    77.75   2.25    88.90    29.51   0.96   0.048   62   10   3.000   4.72
 24  0.951   160.16   2.09   271.77    87.96   1.65   0.084   62   10   1.263   5.28
 25  0.990   104.63    .     150.58    47.64   1.65   0.068   66    8   6.760   4.83
 26  1.048   126.51   3.28   150.19    47.97   1.53   0.062   66    8   3.180   4.98
 27  0.985   133.83   4.97   241.33    73.01   1.93   0.077   66    8   6.870   5.17
 28  0.977   194.71   2.13      .        .     2.37   0.081   66    8   6.000   5.40
 29  1.048   116.86   3.08   109.29    48.09   1.40   0.058   62    8   2.240   4.80
 30   .       62.05   2.74    57.39    23.40   1.24   0.049   62    8   3.830   4.64
 31  1.000    77.19   2.61    68.81    29.34   1.15   0.050   62    8   2.250   4.69
 32  0.949   150.45   2.08   282.53   104.42   1.88   0.076   62    8   6.600   5.20
 33  1.019   138.40   3.13   243.73    92.30   1.93   0.077   62   10   4.750   5.18
 34  0.995   101.79   4.45   131.47    52.53   1.40   0.062   62   10   4.440   4.85
 35  1.050    91.29   2.16    80.92    33.59   1.48   0.058   62   10   5.040   4.65
 36  1.037   129.94   2.03    98.93    37.88   1.39   0.065   62   10   3.510   4.90
 37  1.180    49.40   0.00     6.80    15.45   0.67   0.048   86    4   0.010   4.86
 38  1.170    51.18   1.90    27.50    14.68   0.86   0.057   86    4   0.220   5.00
 39  1.160    71.22   1.44    35.67    32.89   1.05   0.067   86    4   0.630   5.24
 40  1.170    56.03   0.00     0.00    16.07   0.76   0.054   86    4   0.160   4.90
 41  1.140    49.40   0.73     0.00    16.35   0.55   0.062   86    4   0.890   5.13
 42  1.160    54.38   0.00     0.00    14.36   1.04   0.059   86    4   2.310   4.94
 43  1.120   167.59   0.00     0.00    15.60   1.12   0.070   86    4   4.410   5.28
 44  1.170    41.41   0.08     0.00    15.96   0.69   0.055   86    4   2.140   4.87
 45  1.200    54.61   0.00     0.00     8.46   0.58   0.046   86    4   0.420   4.83
 46  1.200    40.72   3.27     0.00    17.84   0.81   0.057   86    4   1.000   4.89
 47  1.200    35.11   0.19     0.00    13.87   0.87   0.060   86    4   0.040   4.92
 48  1.130    29.55   0.00     0.00    12.30   1.01   0.061   86    4   0.030   4.83
 49  1.140    52.35   0.00    14.82    29.21   0.98   0.058   86    2   1.390   4.93
 50  1.180    86.69   0.00    24.93    31.00   0.86   0.052   86    2   0.320   5.32
 51  1.180    76.89   0.85     0.00    23.59   0.75   0.051   86    2   0.460   5.04
 52  1.150    74.47   1.20     0.00    29.11   0.76   0.051   86    2   1.860   5.21
 53  1.180    52.68   0.06     0.00    17.13   0.53   0.044   86    2   0.660   4.97
 54  1.200    70.39   1.94     0.00     9.73   0.66   0.049   86    2   1.460   5.12
 55  1.180    44.79   0.00     0.00     7.46   0.74   0.051   86    2   3.440   4.69
 56  1.220    66.02   4.53     0.00     4.36   0.50   0.039   86    2   0.340   4.87
 57  1.200    33.29   0.00     0.00     9.31   0.60   0.048   86    2   1.700   4.65
 58  1.180    21.82   0.20     0.00     7.29   0.57   0.046   86    2   0.020   4.60
 59  1.200    26.92    .      16.96    11.50   0.73   0.052   86    2   0.540   4.64
 60  1.160    43.10   0.00     0.00     9.77   0.75   0.056   86    2   2.140   4.96
 61  1.160    64.10   0.00    17.09    21.83   0.84   0.054   86    2   1.239   4.88
 62  1.170    44.32   1.51     0.00    13.47   0.60   0.045   86    2   1.450   4.67
 63  1.170    73.67   0.00     0.00    20.07   0.70   0.049   86    2   1.120   4.94
 64  1.050    95.29   1.96   153.23    52.31   1.55    .      86    2   1.620   5.30
 65  1.210    30.32   0.44     5.00     3.50   0.53   0.044   86    2   1.410   4.64
 66  1.200    35.40   4.19     0.00     8.50   0.83   0.053   86    2   0.880   4.69
 67  1.180    41.50   0.00     0.00     9.71   0.62   0.048   88    2   0.900   4.80
 68  1.210    25.30   0.00     0.00     8.47   0.73   0.048   88    2   1.100   4.67
 69  1.200    41.13   1.75     0.00    11.78   0.71   0.055   88    2   1.820   4.84
 70  1.180    29.07   0.96     0.00    13.31   0.72   0.051   88    2   0.240   4.79
 71  1.180    26.19   0.28     0.00     9.85   0.75   0.052   88    2   0.780   4.70
 72  1.180    41.10   0.00     0.00     9.32   0.54   0.054   88    2   1.570   4.72

  Gilbert Pule: Experiment 2 data for statistical analysisNo Outliers 5cm only.SAS  2
                             Project number = 000971-Y5   08:36 Friday, March 9, 2018

                                  The GLM Procedure

                              Class Level Information

            Class                   Levels    Values

            Farm                         2    Kaalfontein Schietfontein

            GrassLandCommunity           2    GM11 Gh15

            Rainfall                     2    593 654

            SlopePosition                3    Bottom Mid Top


                            Data for Analysis of Altitude

                       Number of Observations Read          72
                       Number of Observations Used          69


                       Data for Analysis of Longitude Latitude

                       Number of Observations Read          72
                       Number of Observations Used          70


                           Data for Analysis of SplumDens
                        SplumMeanConCov IndivIntercep K5Depth
                             Org_C5Depth SandPers5Depth
                         SiltPers5Depth P5Depth pHH2O5Depth

                       Number of Observations Read          72
                       Number of Observations Used          72


                         Data for Analysis of BulkDens5Depth

                       Number of Observations Read          72
                       Number of Observations Used          69


                            Data for Analysis of Na5Dpth

                       Number of Observations Read          72
                       Number of Observations Used          68


                            Data for Analysis of Ca5Depth

                       Number of Observations Read          72
                       Number of Observations Used          71


                            Data for Analysis of Mg5Depth

                       Number of Observations Read          72
                       Number of Observations Used          70


                          Data for Analysis of TotalN5Depth

                       Number of Observations Read          72
                       Number of Observations Used          71


NOTE: Variables in each group are consistent with respect to the presence or absence
      of missing values.

  Gilbert Pule: Experiment 2 data for statistical analysisNo Outliers 5cm only.SAS  3
                             Project number = 000971-Y5   08:36 Friday, March 9, 2018

                                  The GLM Procedure

                           Dependent Variable: Altitude

                                         Sum of
 Source                      DF         Squares     Mean Square    F Value    Pr > F

 Model                        5     424758.6691      84951.7338    3914.41    <.0001

 Error                       63       1367.2439         21.7023

 Corrected Total             68     426125.9130


                R-Square     Coeff Var      Root MSE    Altitude Mean

                0.996791      0.302667      4.658571         1539.174


 Source                      DF       Type I SS     Mean Square    F Value    Pr > F

 Farm                         1     424582.6302     424582.6302    19564.0    <.0001
 SlopePosition                2        174.5427         87.2714       4.02    0.0227
 Farm*SlopePosition           2          1.4962          0.7481       0.03    0.9661

  Gilbert Pule: Experiment 2 data for statistical analysisNo Outliers 5cm only.SAS  4
                             Project number = 000971-Y5   08:36 Friday, March 9, 2018

                                  The GLM Procedure

               Level of                 -----------Altitude----------
               Farm               N             Mean          Std Dev

               Kaalfontein       36       1614.27778       4.70021952
               Schietfontein     33       1457.24242       4.90554726


               Level of                 -----------Altitude----------
               SlopePosition      N             Mean          Std Dev

               Bottom            24       1533.87500       80.4445867
               Mid               23       1539.21739       80.0982105
               Top               22       1544.90909       80.1016670


      Level of          Level of                 -----------Altitude----------
      Farm              SlopePosition      N             Mean          Std Dev

      Kaalfontein       Bottom            12       1612.50000       4.35889894
      Kaalfontein       Mid               12       1614.08333       4.96273996
      Kaalfontein       Top               12       1616.25000       4.35107092
      Schietfontein     Bottom            12       1455.25000       4.91981153
      Schietfontein     Mid               11       1457.54545       4.92673597
      Schietfontein     Top               10       1459.30000       4.34741302

  Gilbert Pule: Experiment 2 data for statistical analysisNo Outliers 5cm only.SAS  5
                             Project number = 000971-Y5   08:36 Friday, March 9, 2018

                                  The GLM Procedure

                             t Tests (LSD) for Altitude

       NOTE: This test controls the Type I comparisonwise error rate, not the
                             experimentwise error rate.


                        Alpha                            0.05
                        Error Degrees of Freedom           63
                        Error Mean Square            21.70228
                        Critical Value of t           1.99834
                        Least Significant Difference   2.2436
                        Harmonic Mean of Cell Sizes  34.43478

                           NOTE: Cell sizes are not equal.


             Means with the same letter are not significantly different.


              t Grouping          Mean      N    Farm

                       A      1614.278     36    Kaalfontein

                       B      1457.242     33    Schietfontein

  Gilbert Pule: Experiment 2 data for statistical analysisNo Outliers 5cm only.SAS  6
                             Project number = 000971-Y5   08:36 Friday, March 9, 2018

                                  The GLM Procedure

                             t Tests (LSD) for Altitude

       NOTE: This test controls the Type I comparisonwise error rate, not the
                             experimentwise error rate.


                        Alpha                            0.05
                        Error Degrees of Freedom           63
                        Error Mean Square            21.70228
                        Critical Value of t           1.99834
                        Least Significant Difference   2.7469
                        Harmonic Mean of Cell Sizes    22.971

                           NOTE: Cell sizes are not equal.


             Means with the same letter are not significantly different.


                                                   Slope
                t Grouping          Mean      N    Position

                         A      1544.909     22    Top

                         B      1539.217     23    Mid

                         C      1533.875     24    Bottom

  Gilbert Pule: Experiment 2 data for statistical analysisNo Outliers 5cm only.SAS  7
                             Project number = 000971-Y5   08:36 Friday, March 9, 2018

                                  The GLM Procedure

                          Dependent Variable: Longitude

                                         Sum of
 Source                      DF         Squares     Mean Square    F Value    Pr > F

 Model                        5      1.71641004      0.34328201    59595.2    <.0001

 Error                       64      0.00036865      0.00000576

 Corrected Total             69      1.71677869


               R-Square     Coeff Var      Root MSE    Longitude Mean

               0.999785      0.009256      0.002400          25.93067


 Source                      DF       Type I SS     Mean Square    F Value    Pr > F

 Farm                         1      1.71637520      1.71637520     297970    <.0001
 SlopePosition                2      0.00000822      0.00000411       0.71    0.4937
 Farm*SlopePosition           2      0.00002661      0.00001331       2.31    0.1075

  Gilbert Pule: Experiment 2 data for statistical analysisNo Outliers 5cm only.SAS  8
                             Project number = 000971-Y5   08:36 Friday, March 9, 2018

                                  The GLM Procedure

                           Dependent Variable: Latitude

                                         Sum of
 Source                      DF         Squares     Mean Square    F Value    Pr > F

 Model                        5     25.79075174      5.15815035    3659825    <.0001

 Error                       64      0.00009020      0.00000141

 Corrected Total             69     25.79084195


                R-Square     Coeff Var      Root MSE    Latitude Mean

                0.999997      0.004231      0.001187         28.05684


 Source                      DF       Type I SS     Mean Square    F Value    Pr > F

 Farm                         1     25.79074933     25.79074933     1.83E7    <.0001
 SlopePosition                2      0.00000130      0.00000065       0.46    0.6317
 Farm*SlopePosition           2      0.00000111      0.00000056       0.39    0.6760

  Gilbert Pule: Experiment 2 data for statistical analysisNo Outliers 5cm only.SAS  9
                             Project number = 000971-Y5   08:36 Friday, March 9, 2018

                                  The GLM Procedure

 Level of               ----------Longitude---------    ----------Latitude----------
 Farm              N            Mean         Std Dev            Mean         Std Dev

 Kaalfontein      36      26.0828506      0.00335588      27.4669514      0.00097073
 Schietfontein    34      25.7695476      0.00053149      28.6814312      0.00134429


 Level of               ----------Longitude---------    ----------Latitude----------
 SlopePosition     N            Mean         Std Dev            Mean         Std Dev

 Bottom           24      25.9265175      0.16092259      28.0740496      0.62048501
 Mid              24      25.9257329      0.15949875      28.0741588      0.62021343
 Top              22      25.9406014      0.15929659      28.0191777      0.61887007


 Level of      Level of         --------Longitude-------- ---------Latitude--------
 Farm          SlopePosition  N         Mean      Std Dev         Mean      Std Dev

 Kaalfontein   Bottom        12   26.0840142   0.00507767   27.4666300   0.00098021
 Kaalfontein   Mid           12   26.0818717   0.00105859   27.4670050   0.00099145
 Kaalfontein   Top           12   26.0826658   0.00252396   27.4672192   0.00092761
 Schietfontein Bottom        12   25.7690208   0.00036340   28.6814692   0.00141627
 Schietfontein Mid           12   25.7695942   0.00027665   28.6813125   0.00138389
 Schietfontein Top           10   25.7701240   0.00019699   28.6815280   0.00134264

  Gilbert Pule: Experiment 2 data for statistical analysisNo Outliers 5cm only.SAS 10
                             Project number = 000971-Y5   08:36 Friday, March 9, 2018

                                  The GLM Procedure

                             t Tests (LSD) for Longitude

       NOTE: This test controls the Type I comparisonwise error rate, not the
                             experimentwise error rate.


                        Alpha                            0.05
                        Error Degrees of Freedom           64
                        Error Mean Square             5.76E-6
                        Critical Value of t           1.99773
                        Least Significant Difference   0.0011
                        Harmonic Mean of Cell Sizes  34.97143

                           NOTE: Cell sizes are not equal.


             Means with the same letter are not significantly different.


              t Grouping          Mean      N    Farm

                       A     26.082851     36    Kaalfontein

                       B     25.769548     34    Schietfontein

  Gilbert Pule: Experiment 2 data for statistical analysisNo Outliers 5cm only.SAS 11
                             Project number = 000971-Y5   08:36 Friday, March 9, 2018

                                  The GLM Procedure

                             t Tests (LSD) for Latitude

       NOTE: This test controls the Type I comparisonwise error rate, not the
                             experimentwise error rate.


                        Alpha                            0.05
                        Error Degrees of Freedom           64
                        Error Mean Square            1.409E-6
                        Critical Value of t           1.99773
                        Least Significant Difference   0.0006
                        Harmonic Mean of Cell Sizes  34.97143

                           NOTE: Cell sizes are not equal.


             Means with the same letter are not significantly different.


              t Grouping          Mean      N    Farm

                       A     28.681431     34    Schietfontein

                       B     27.466951     36    Kaalfontein

  Gilbert Pule: Experiment 2 data for statistical analysisNo Outliers 5cm only.SAS 12
                             Project number = 000971-Y5   08:36 Friday, March 9, 2018

                                  The GLM Procedure

                             t Tests (LSD) for Longitude

       NOTE: This test controls the Type I comparisonwise error rate, not the
                             experimentwise error rate.


                        Alpha                            0.05
                        Error Degrees of Freedom           64
                        Error Mean Square             5.76E-6
                        Critical Value of t           1.99773
                        Least Significant Difference   0.0014
                        Harmonic Mean of Cell Sizes  23.29412

                           NOTE: Cell sizes are not equal.


             Means with the same letter are not significantly different.


                                                   Slope
                t Grouping          Mean      N    Position

                         A     25.940601     22    Top

                         B     25.926518     24    Bottom
                         B
                         B     25.925733     24    Mid

  Gilbert Pule: Experiment 2 data for statistical analysisNo Outliers 5cm only.SAS 13
                             Project number = 000971-Y5   08:36 Friday, March 9, 2018

                                  The GLM Procedure

                             t Tests (LSD) for Latitude

       NOTE: This test controls the Type I comparisonwise error rate, not the
                             experimentwise error rate.


                        Alpha                            0.05
                        Error Degrees of Freedom           64
                        Error Mean Square            1.409E-6
                        Critical Value of t           1.99773
                        Least Significant Difference   0.0007
                        Harmonic Mean of Cell Sizes  23.29412

                           NOTE: Cell sizes are not equal.


             Means with the same letter are not significantly different.


                                                   Slope
                t Grouping          Mean      N    Position

                         A     28.074159     24    Mid
                         A
                         A     28.074050     24    Bottom

                         B     28.019178     22    Top

  Gilbert Pule: Experiment 2 data for statistical analysisNo Outliers 5cm only.SAS 14
                             Project number = 000971-Y5   08:36 Friday, March 9, 2018

                                  The GLM Procedure

                          Dependent Variable: SplumDens

                                         Sum of
 Source                      DF         Squares     Mean Square    F Value    Pr > F

 Model                        5     12702.56944      2540.51389       7.64    <.0001

 Error                       66     21945.08333       332.50126

 Corrected Total             71     34647.65278


               R-Square     Coeff Var      Root MSE    SplumDens Mean

               0.366621      57.55776      18.23462          31.68056


 Source                      DF       Type I SS     Mean Square    F Value    Pr > F

 Farm                         1     5356.125000     5356.125000      16.11    0.0002
 SlopePosition                2     2638.361111     1319.180556       3.97    0.0236
 Farm*SlopePosition           2     4708.083333     2354.041667       7.08    0.0016

  Gilbert Pule: Experiment 2 data for statistical analysisNo Outliers 5cm only.SAS 15
                             Project number = 000971-Y5   08:36 Friday, March 9, 2018

                                  The GLM Procedure

                       Dependent Variable: SplumMeanConCov

                                         Sum of
 Source                      DF         Squares     Mean Square    F Value    Pr > F

 Model                        5      4.34661129      0.86932226       2.65    0.0301

 Error                       66     21.61064058      0.32743395

 Corrected Total             71     25.95725188


            R-Square     Coeff Var      Root MSE    SplumMeanConCov Mean

            0.167453      46.43845      0.572218                1.232208


 Source                      DF       Type I SS     Mean Square    F Value    Pr > F

 Farm                         1      1.90808112      1.90808112       5.83    0.0186
 SlopePosition                2      2.40637758      1.20318879       3.67    0.0307
 Farm*SlopePosition           2      0.03215258      0.01607629       0.05    0.9521

  Gilbert Pule: Experiment 2 data for statistical analysisNo Outliers 5cm only.SAS 16
                             Project number = 000971-Y5   08:36 Friday, March 9, 2018

                                  The GLM Procedure

                        Dependent Variable: IndivIntercep

                                         Sum of
 Source                      DF         Squares     Mean Square    F Value    Pr > F

 Model                        5      388.777778       77.755556       2.13    0.0728

 Error                       66     2410.500000       36.522727

 Corrected Total             71     2799.277778


             R-Square     Coeff Var      Root MSE    IndivIntercep Mean

             0.138885      53.45517      6.043404              11.30556


 Source                      DF       Type I SS     Mean Square    F Value    Pr > F

 Farm                         1     180.5000000     180.5000000       4.94    0.0296
 SlopePosition                2      50.0277778      25.0138889       0.68    0.5077
 Farm*SlopePosition           2     158.2500000      79.1250000       2.17    0.1227

  Gilbert Pule: Experiment 2 data for statistical analysisNo Outliers 5cm only.SAS 17
                             Project number = 000971-Y5   08:36 Friday, March 9, 2018

                                  The GLM Procedure

                           Dependent Variable: K5Depth

                                         Sum of
 Source                      DF         Squares     Mean Square    F Value    Pr > F

 Model                        5      71473.0866      14294.6173      10.83    <.0001

 Error                       66      87074.6859       1319.3134

 Corrected Total             71     158547.7725


                R-Square     Coeff Var      Root MSE    K5Depth Mean

                0.450798      43.30577      36.32235        83.87417


 Source                      DF       Type I SS     Mean Square    F Value    Pr > F

 Farm                         1     69946.72694     69946.72694      53.02    <.0001
 SlopePosition                2       101.82563        50.91282       0.04    0.9622
 Farm*SlopePosition           2      1424.53404       712.26702       0.54    0.5854

  Gilbert Pule: Experiment 2 data for statistical analysisNo Outliers 5cm only.SAS 18
                             Project number = 000971-Y5   08:36 Friday, March 9, 2018

                                  The GLM Procedure

                         Dependent Variable: Org_C5Depth

                                         Sum of
 Source                      DF         Squares     Mean Square    F Value    Pr > F

 Model                        5     10.56347917      2.11269583      33.74    <.0001

 Error                       66      4.13260833      0.06261528

 Corrected Total             71     14.69608750


              R-Square     Coeff Var      Root MSE    Org_C5Depth Mean

              0.718795      21.87807      0.250230            1.143750


 Source                      DF       Type I SS     Mean Square    F Value    Pr > F

 Farm                         1     10.29823472     10.29823472     164.47    <.0001
 SlopePosition                2      0.16043333      0.08021667       1.28    0.2845
 Farm*SlopePosition           2      0.10481111      0.05240556       0.84    0.4376

  Gilbert Pule: Experiment 2 data for statistical analysisNo Outliers 5cm only.SAS 19
                             Project number = 000971-Y5   08:36 Friday, March 9, 2018

                                  The GLM Procedure

                        Dependent Variable: SandPers5Depth

                                         Sum of
 Source                      DF         Squares     Mean Square    F Value    Pr > F

 Model                        5     9530.000000     1906.000000     898.54    <.0001

 Error                       66      140.000000        2.121212

 Corrected Total             71     9670.000000


             R-Square     Coeff Var      Root MSE    SandPers5Depth Mean

             0.985522      1.946243      1.456438               74.83333


 Source                      DF       Type I SS     Mean Square    F Value    Pr > F

 Farm                         1     9522.000000     9522.000000    4488.94    <.0001
 SlopePosition                2        4.000000        2.000000       0.94    0.3947
 Farm*SlopePosition           2        4.000000        2.000000       0.94    0.3947

  Gilbert Pule: Experiment 2 data for statistical analysisNo Outliers 5cm only.SAS 20
                             Project number = 000971-Y5   08:36 Friday, March 9, 2018

                                  The GLM Procedure

                        Dependent Variable: SiltPers5Depth

                                         Sum of
 Source                      DF         Squares     Mean Square    F Value    Pr > F

 Model                        5     680.0000000     136.0000000     280.50    <.0001

 Error                       66      32.0000000       0.4848485

 Corrected Total             71     712.0000000


             R-Square     Coeff Var      Root MSE    SiltPers5Depth Mean

             0.955056      12.28783      0.696311               5.666667


 Source                      DF       Type I SS     Mean Square    F Value    Pr > F

 Farm                         1     648.0000000     648.0000000    1336.50    <.0001
 SlopePosition                2      16.0000000       8.0000000      16.50    <.0001
 Farm*SlopePosition           2      16.0000000       8.0000000      16.50    <.0001

  Gilbert Pule: Experiment 2 data for statistical analysisNo Outliers 5cm only.SAS 21
                             Project number = 000971-Y5   08:36 Friday, March 9, 2018

                                  The GLM Procedure

                           Dependent Variable: P5Depth

                                         Sum of
 Source                      DF         Squares     Mean Square    F Value    Pr > F

 Model                        5     171.9289174      34.3857835      20.77    <.0001

 Error                       66     109.2618992       1.6554833

 Corrected Total             71     281.1908166


                R-Square     Coeff Var      Root MSE    P5Depth Mean

                0.611431      48.85468      1.286656        2.633639


 Source                      DF       Type I SS     Mean Square    F Value    Pr > F

 Farm                         1     162.5524702     162.5524702      98.19    <.0001
 SlopePosition                2       4.1582487       2.0791243       1.26    0.2915
 Farm*SlopePosition           2       5.2181985       2.6090993       1.58    0.2145

  Gilbert Pule: Experiment 2 data for statistical analysisNo Outliers 5cm only.SAS 22
                             Project number = 000971-Y5   08:36 Friday, March 9, 2018

                                  The GLM Procedure

                         Dependent Variable: pHH2O5Depth

                                         Sum of
 Source                      DF         Squares     Mean Square    F Value    Pr > F

 Model                        5      0.28921111      0.05784222       1.26    0.2896

 Error                       66      3.01810000      0.04572879

 Corrected Total             71      3.30731111


              R-Square     Coeff Var      Root MSE    pHH2O5Depth Mean

              0.087446      4.378537      0.213843            4.883889


 Source                      DF       Type I SS     Mean Square    F Value    Pr > F

 Farm                         1      0.01445000      0.01445000       0.32    0.5759
 SlopePosition                2      0.00923611      0.00461806       0.10    0.9041
 Farm*SlopePosition           2      0.26552500      0.13276250       2.90    0.0619

  Gilbert Pule: Experiment 2 data for statistical analysisNo Outliers 5cm only.SAS 23
                             Project number = 000971-Y5   08:36 Friday, March 9, 2018

                                  The GLM Procedure

 Level of               ----------SplumDens---------    -------SplumMeanConCov------
 Farm              N            Mean         Std Dev            Mean         Std Dev

 Kaalfontein      36      23.0555556      14.2867460      1.39500000      0.64581510
 Schietfontein    36      40.3055556      25.1553112      1.06941667      0.51965568

 Level of               --------IndivIntercep-------    -----------K5Depth----------
 Farm              N            Mean         Std Dev            Mean         Std Dev

 Kaalfontein      36       9.7222222      4.60193196      115.042778      42.5562015
 Schietfontein    36      12.8888889      7.32423678       52.705556      26.8407928

 Level of               ---------Org_C5Depth--------    -------SandPers5Depth-------
 Farm              N            Mean         Std Dev            Mean         Std Dev

 Kaalfontein      36      1.52194444      0.28723131      63.3333333      1.91236577
 Schietfontein    36      0.76555556      0.20772846      86.3333333      0.75592895

 Level of               -------SiltPers5Depth-------    -----------P5Depth----------
 Farm              N            Mean         Std Dev            Mean         Std Dev

 Kaalfontein      36      8.66666667      0.95618289      4.13619444      1.56901432
 Schietfontein    36      2.66666667      0.95618289      1.13108333      0.96325547

                Level of               ---------pHH2O5Depth---------
                Farm              N            Mean          Std Dev

                Kaalfontein      36      4.86972222       0.23071920
                Schietfontein    36      4.89805556       0.20211481


 Level of               ----------SplumDens---------    -------SplumMeanConCov------
 SlopePosition     N            Mean         Std Dev            Mean         Std Dev

 Bottom           24      39.3750000      29.6703812      0.98612500      0.57687135
 Mid              24      31.0833333      17.6756198      1.28658333      0.54763062
 Top              24      24.5833333      14.1049380      1.42391667      0.62551675

 Level of               --------IndivIntercep-------    -----------K5Depth----------
 SlopePosition     N            Mean         Std Dev            Mean         Std Dev

 Bottom           24      12.3333333      7.64236855      84.1675000      53.6082625
 Mid              24      11.2916667      5.98170520      85.1616667      42.3465312
 Top              24      10.2916667      5.03448254      82.2933333      47.1368190

 Level of               ---------Org_C5Depth--------    -------SandPers5Depth-------
 SlopePosition     N            Mean         Std Dev            Mean         Std Dev

 Bottom           24      1.16708333      0.40081959      74.6666667      11.6569318
 Mid              24      1.07791667      0.43359949      74.6666667      11.6569318
 Top              24      1.18625000      0.53227822      75.1666667      12.1857603

 Level of               -------SiltPers5Depth-------    -----------P5Depth----------
 SlopePosition     N            Mean         Std Dev            Mean         Std Dev

 Bottom           24      6.33333333      2.47889644      2.68333333      2.25091221
 Mid              24      5.33333333      3.47245893      2.31762500      1.56425998
 Top              24      5.33333333      3.47245893      2.89995833      2.12870364

                Level of               ---------pHH2O5Depth---------
                SlopePosition     N            Mean          Std Dev

                Bottom           24      4.89916667       0.21449469
                Mid              24      4.88041667       0.21786472
                Top              24      4.87208333       0.22343133


 Level of      Level of         --------SplumDens-------- -----SplumMeanConCov-----
 Farm          SlopePosition  N         Mean      Std Dev         Mean      Std Dev

 Kaalfontein   Bottom        12   19.7500000   10.4196056   1.14083333   0.48690970
 Kaalfontein   Mid           12   25.2500000   17.1258709   1.47833333   0.54825066
 Kaalfontein   Top           12   24.1666667   15.1347483   1.56583333   0.82432847
 Schietfontein Bottom        12   59.0000000   29.8633250   0.83141667   0.63758272
 Schietfontein Mid           12   36.9166667   16.9032452   1.09483333   0.49624807
 Schietfontein Top           12   25.0000000   13.6581644   1.28200000   0.30765654

 Level of      Level of         ------IndivIntercep------ ---------K5Depth---------
 Farm          SlopePosition  N         Mean      Std Dev         Mean      Std Dev

 Kaalfontein   Bottom        12    9.0000000   4.06761042   109.951667   57.0722282
 Kaalfontein   Mid           12    9.5833333   5.03548018   116.205833   34.7511354
 Kaalfontein   Top           12   10.5833333   4.90747729   118.970833   35.3681319
 Schietfontein Bottom        12   15.6666667   9.01849951    58.383333   36.0719786
 Schietfontein Mid           12   13.0000000   6.56436662    54.117500   20.9554459
 Schietfontein Top           12   10.0000000   5.35978290    45.615833   21.4424064

 Level of      Level of         -------Org_C5Depth------- ------SandPers5Depth-----
 Farm          SlopePosition  N         Mean      Std Dev         Mean      Std Dev

 Kaalfontein   Bottom        12   1.50000000   0.23977262   63.3333333   1.96946386
 Kaalfontein   Mid           12   1.45333333   0.25748198   63.3333333   1.96946386
 Kaalfontein   Top           12   1.61250000   0.35229443   63.3333333   1.96946386
 Schietfontein Bottom        12   0.83416667   0.19133304   86.0000000   0.00000000
 Schietfontein Mid           12   0.70250000   0.13896533   86.0000000   0.00000000
 Schietfontein Top           12   0.76000000   0.26809089   87.0000000   1.04446594

 Level of      Level of         ------SiltPers5Depth----- ---------P5Depth---------
 Farm          SlopePosition  N         Mean      Std Dev         Mean      Std Dev

 Kaalfontein   Bottom        12   8.66666667   0.98473193   4.34500000   1.67832438
 Kaalfontein   Mid           12   8.66666667   0.98473193   3.44108333   1.17224784
 Kaalfontein   Top           12   8.66666667   0.98473193   4.62250000   1.67613259
 Schietfontein Bottom        12   4.00000000   0.00000000   1.02166667   1.32391179
 Schietfontein Mid           12   2.00000000   0.00000000   1.19416667   0.99413241
 Schietfontein Top           12   2.00000000   0.00000000   1.17741667   0.43760722

            Level of      Level of         ---------pHH2O5Depth---------
            Farm          SlopePosition  N         Mean          Std Dev

            Kaalfontein   Bottom        12   4.82416667       0.24343968
            Kaalfontein   Mid           12   4.84416667       0.20192070
            Kaalfontein   Top           12   4.94083333       0.24666718
            Schietfontein Bottom        12   4.97416667       0.15704419
            Schietfontein Mid           12   4.91666667       0.23580937
            Schietfontein Top           12   4.80333333       0.18227518

  Gilbert Pule: Experiment 2 data for statistical analysisNo Outliers 5cm only.SAS 24
                             Project number = 000971-Y5   08:36 Friday, March 9, 2018

                                  The GLM Procedure

                             t Tests (LSD) for SplumDens

       NOTE: This test controls the Type I comparisonwise error rate, not the
                             experimentwise error rate.


                        Alpha                            0.05
                        Error Degrees of Freedom           66
                        Error Mean Square            332.5013
                        Critical Value of t           1.99656
                        Least Significant Difference   8.5811


             Means with the same letter are not significantly different.


              t Grouping          Mean      N    Farm

                       A        40.306     36    Schietfontein

                       B        23.056     36    Kaalfontein

  Gilbert Pule: Experiment 2 data for statistical analysisNo Outliers 5cm only.SAS 25
                             Project number = 000971-Y5   08:36 Friday, March 9, 2018

                                  The GLM Procedure

                          t Tests (LSD) for SplumMeanConCov

       NOTE: This test controls the Type I comparisonwise error rate, not the
                             experimentwise error rate.


                        Alpha                            0.05
                        Error Degrees of Freedom           66
                        Error Mean Square            0.327434
                        Critical Value of t           1.99656
                        Least Significant Difference   0.2693


             Means with the same letter are not significantly different.


              t Grouping          Mean      N    Farm

                       A        1.3950     36    Kaalfontein

                       B        1.0694     36    Schietfontein

  Gilbert Pule: Experiment 2 data for statistical analysisNo Outliers 5cm only.SAS 26
                             Project number = 000971-Y5   08:36 Friday, March 9, 2018

                                  The GLM Procedure

                           t Tests (LSD) for IndivIntercep

       NOTE: This test controls the Type I comparisonwise error rate, not the
                             experimentwise error rate.


                        Alpha                            0.05
                        Error Degrees of Freedom           66
                        Error Mean Square            36.52273
                        Critical Value of t           1.99656
                        Least Significant Difference    2.844


             Means with the same letter are not significantly different.


              t Grouping          Mean      N    Farm

                       A        12.889     36    Schietfontein

                       B         9.722     36    Kaalfontein

  Gilbert Pule: Experiment 2 data for statistical analysisNo Outliers 5cm only.SAS 27
                             Project number = 000971-Y5   08:36 Friday, March 9, 2018

                                  The GLM Procedure

                              t Tests (LSD) for K5Depth

       NOTE: This test controls the Type I comparisonwise error rate, not the
                             experimentwise error rate.


                        Alpha                            0.05
                        Error Degrees of Freedom           66
                        Error Mean Square            1319.313
                        Critical Value of t           1.99656
                        Least Significant Difference   17.093


             Means with the same letter are not significantly different.


              t Grouping          Mean      N    Farm

                       A       115.043     36    Kaalfontein

                       B        52.706     36    Schietfontein

  Gilbert Pule: Experiment 2 data for statistical analysisNo Outliers 5cm only.SAS 28
                             Project number = 000971-Y5   08:36 Friday, March 9, 2018

                                  The GLM Procedure

                            t Tests (LSD) for Org_C5Depth

       NOTE: This test controls the Type I comparisonwise error rate, not the
                             experimentwise error rate.


                        Alpha                            0.05
                        Error Degrees of Freedom           66
                        Error Mean Square            0.062615
                        Critical Value of t           1.99656
                        Least Significant Difference   0.1178


             Means with the same letter are not significantly different.


              t Grouping          Mean      N    Farm

                       A       1.52194     36    Kaalfontein

                       B       0.76556     36    Schietfontein

  Gilbert Pule: Experiment 2 data for statistical analysisNo Outliers 5cm only.SAS 29
                             Project number = 000971-Y5   08:36 Friday, March 9, 2018

                                  The GLM Procedure

                          t Tests (LSD) for SandPers5Depth

       NOTE: This test controls the Type I comparisonwise error rate, not the
                             experimentwise error rate.


                        Alpha                            0.05
                        Error Degrees of Freedom           66
                        Error Mean Square            2.121212
                        Critical Value of t           1.99656
                        Least Significant Difference   0.6854


             Means with the same letter are not significantly different.


              t Grouping          Mean      N    Farm

                       A       86.3333     36    Schietfontein

                       B       63.3333     36    Kaalfontein

  Gilbert Pule: Experiment 2 data for statistical analysisNo Outliers 5cm only.SAS 30
                             Project number = 000971-Y5   08:36 Friday, March 9, 2018

                                  The GLM Procedure

                          t Tests (LSD) for SiltPers5Depth

       NOTE: This test controls the Type I comparisonwise error rate, not the
                             experimentwise error rate.


                        Alpha                            0.05
                        Error Degrees of Freedom           66
                        Error Mean Square            0.484848
                        Critical Value of t           1.99656
                        Least Significant Difference   0.3277


             Means with the same letter are not significantly different.


              t Grouping          Mean      N    Farm

                       A        8.6667     36    Kaalfontein

                       B        2.6667     36    Schietfontein

  Gilbert Pule: Experiment 2 data for statistical analysisNo Outliers 5cm only.SAS 31
                             Project number = 000971-Y5   08:36 Friday, March 9, 2018

                                  The GLM Procedure

                              t Tests (LSD) for P5Depth

       NOTE: This test controls the Type I comparisonwise error rate, not the
                             experimentwise error rate.


                        Alpha                            0.05
                        Error Degrees of Freedom           66
                        Error Mean Square            1.655483
                        Critical Value of t           1.99656
                        Least Significant Difference   0.6055


             Means with the same letter are not significantly different.


              t Grouping          Mean      N    Farm

                       A        4.1362     36    Kaalfontein

                       B        1.1311     36    Schietfontein

  Gilbert Pule: Experiment 2 data for statistical analysisNo Outliers 5cm only.SAS 32
                             Project number = 000971-Y5   08:36 Friday, March 9, 2018

                                  The GLM Procedure

                            t Tests (LSD) for pHH2O5Depth

       NOTE: This test controls the Type I comparisonwise error rate, not the
                             experimentwise error rate.


                        Alpha                            0.05
                        Error Degrees of Freedom           66
                        Error Mean Square            0.045729
                        Critical Value of t           1.99656
                        Least Significant Difference   0.1006


             Means with the same letter are not significantly different.


              t Grouping          Mean      N    Farm

                       A       4.89806     36    Schietfontein
                       A
                       A       4.86972     36    Kaalfontein

  Gilbert Pule: Experiment 2 data for statistical analysisNo Outliers 5cm only.SAS 33
                             Project number = 000971-Y5   08:36 Friday, March 9, 2018

                                  The GLM Procedure

                             t Tests (LSD) for SplumDens

       NOTE: This test controls the Type I comparisonwise error rate, not the
                             experimentwise error rate.


                        Alpha                            0.05
                        Error Degrees of Freedom           66
                        Error Mean Square            332.5013
                        Critical Value of t           1.99656
                        Least Significant Difference    10.51


             Means with the same letter are not significantly different.


                                                      Slope
                   t Grouping          Mean      N    Position

                            A        39.375     24    Bottom
                            A
                       B    A        31.083     24    Mid
                       B
                       B             24.583     24    Top

  Gilbert Pule: Experiment 2 data for statistical analysisNo Outliers 5cm only.SAS 34
                             Project number = 000971-Y5   08:36 Friday, March 9, 2018

                                  The GLM Procedure

                          t Tests (LSD) for SplumMeanConCov

       NOTE: This test controls the Type I comparisonwise error rate, not the
                             experimentwise error rate.


                        Alpha                            0.05
                        Error Degrees of Freedom           66
                        Error Mean Square            0.327434
                        Critical Value of t           1.99656
                        Least Significant Difference   0.3298


             Means with the same letter are not significantly different.


                                                      Slope
                   t Grouping          Mean      N    Position

                            A        1.4239     24    Top
                            A
                       B    A        1.2866     24    Mid
                       B
                       B             0.9861     24    Bottom

  Gilbert Pule: Experiment 2 data for statistical analysisNo Outliers 5cm only.SAS 35
                             Project number = 000971-Y5   08:36 Friday, March 9, 2018

                                  The GLM Procedure

                           t Tests (LSD) for IndivIntercep

       NOTE: This test controls the Type I comparisonwise error rate, not the
                             experimentwise error rate.


                        Alpha                            0.05
                        Error Degrees of Freedom           66
                        Error Mean Square            36.52273
                        Critical Value of t           1.99656
                        Least Significant Difference   3.4832


             Means with the same letter are not significantly different.


                                                   Slope
                t Grouping          Mean      N    Position

                         A        12.333     24    Bottom
                         A
                         A        11.292     24    Mid
                         A
                         A        10.292     24    Top

  Gilbert Pule: Experiment 2 data for statistical analysisNo Outliers 5cm only.SAS 36
                             Project number = 000971-Y5   08:36 Friday, March 9, 2018

                                  The GLM Procedure

                              t Tests (LSD) for K5Depth

       NOTE: This test controls the Type I comparisonwise error rate, not the
                             experimentwise error rate.


                        Alpha                            0.05
                        Error Degrees of Freedom           66
                        Error Mean Square            1319.313
                        Critical Value of t           1.99656
                        Least Significant Difference   20.935


             Means with the same letter are not significantly different.


                                                   Slope
                t Grouping          Mean      N    Position

                         A         85.16     24    Mid
                         A
                         A         84.17     24    Bottom
                         A
                         A         82.29     24    Top

  Gilbert Pule: Experiment 2 data for statistical analysisNo Outliers 5cm only.SAS 37
                             Project number = 000971-Y5   08:36 Friday, March 9, 2018

                                  The GLM Procedure

                            t Tests (LSD) for Org_C5Depth

       NOTE: This test controls the Type I comparisonwise error rate, not the
                             experimentwise error rate.


                        Alpha                            0.05
                        Error Degrees of Freedom           66
                        Error Mean Square            0.062615
                        Critical Value of t           1.99656
                        Least Significant Difference   0.1442


             Means with the same letter are not significantly different.


                                                   Slope
                t Grouping          Mean      N    Position

                         A       1.18625     24    Top
                         A
                         A       1.16708     24    Bottom
                         A
                         A       1.07792     24    Mid

  Gilbert Pule: Experiment 2 data for statistical analysisNo Outliers 5cm only.SAS 38
                             Project number = 000971-Y5   08:36 Friday, March 9, 2018

                                  The GLM Procedure

                          t Tests (LSD) for SandPers5Depth

       NOTE: This test controls the Type I comparisonwise error rate, not the
                             experimentwise error rate.


                        Alpha                            0.05
                        Error Degrees of Freedom           66
                        Error Mean Square            2.121212
                        Critical Value of t           1.99656
                        Least Significant Difference   0.8394


             Means with the same letter are not significantly different.


                                                   Slope
                t Grouping          Mean      N    Position

                         A       75.1667     24    Top
                         A
                         A       74.6667     24    Mid
                         A
                         A       74.6667     24    Bottom

  Gilbert Pule: Experiment 2 data for statistical analysisNo Outliers 5cm only.SAS 39
                             Project number = 000971-Y5   08:36 Friday, March 9, 2018

                                  The GLM Procedure

                          t Tests (LSD) for SiltPers5Depth

       NOTE: This test controls the Type I comparisonwise error rate, not the
                             experimentwise error rate.


                        Alpha                            0.05
                        Error Degrees of Freedom           66
                        Error Mean Square            0.484848
                        Critical Value of t           1.99656
                        Least Significant Difference   0.4013


             Means with the same letter are not significantly different.


                                                   Slope
                t Grouping          Mean      N    Position

                         A        6.3333     24    Bottom

                         B        5.3333     24    Mid
                         B
                         B        5.3333     24    Top

  Gilbert Pule: Experiment 2 data for statistical analysisNo Outliers 5cm only.SAS 40
                             Project number = 000971-Y5   08:36 Friday, March 9, 2018

                                  The GLM Procedure

                              t Tests (LSD) for P5Depth

       NOTE: This test controls the Type I comparisonwise error rate, not the
                             experimentwise error rate.


                        Alpha                            0.05
                        Error Degrees of Freedom           66
                        Error Mean Square            1.655483
                        Critical Value of t           1.99656
                        Least Significant Difference   0.7416


             Means with the same letter are not significantly different.


                                                   Slope
                t Grouping          Mean      N    Position

                         A        2.9000     24    Top
                         A
                         A        2.6833     24    Bottom
                         A
                         A        2.3176     24    Mid

  Gilbert Pule: Experiment 2 data for statistical analysisNo Outliers 5cm only.SAS 41
                             Project number = 000971-Y5   08:36 Friday, March 9, 2018

                                  The GLM Procedure

                            t Tests (LSD) for pHH2O5Depth

       NOTE: This test controls the Type I comparisonwise error rate, not the
                             experimentwise error rate.


                        Alpha                            0.05
                        Error Degrees of Freedom           66
                        Error Mean Square            0.045729
                        Critical Value of t           1.99656
                        Least Significant Difference   0.1233


             Means with the same letter are not significantly different.


                                                   Slope
                t Grouping          Mean      N    Position

                         A       4.89917     24    Bottom
                         A
                         A       4.88042     24    Mid
                         A
                         A       4.87208     24    Top

  Gilbert Pule: Experiment 2 data for statistical analysisNo Outliers 5cm only.SAS 42
                             Project number = 000971-Y5   08:36 Friday, March 9, 2018

                                  The GLM Procedure

                        Dependent Variable: BulkDens5Depth

                                         Sum of
 Source                      DF         Squares     Mean Square    F Value    Pr > F

 Model                        5      0.45275940      0.09055188      66.42    <.0001

 Error                       63      0.08589182      0.00136336

 Corrected Total             68      0.53865122


             R-Square     Coeff Var      Root MSE    BulkDens5Depth Mean

             0.840543      3.367484      0.036924               1.096478


 Source                      DF       Type I SS     Mean Square    F Value    Pr > F

 Farm                         1      0.45106469      0.45106469     330.85    <.0001
 SlopePosition                2      0.00147626      0.00073813       0.54    0.5846
 Farm*SlopePosition           2      0.00021845      0.00010922       0.08    0.9231

  Gilbert Pule: Experiment 2 data for statistical analysisNo Outliers 5cm only.SAS 43
                             Project number = 000971-Y5   08:36 Friday, March 9, 2018

                                  The GLM Procedure

               Level of                 --------BulkDens5Depth-------
               Farm               N             Mean          Std Dev

               Kaalfontein       33       1.01203030       0.04074730
               Schietfontein     36       1.17388889       0.03137586


               Level of                 --------BulkDens5Depth-------
               SlopePosition      N             Mean          Std Dev

               Bottom            23       1.09160870       0.08781216
               Mid               23       1.10269565       0.09036815
               Top               23       1.09513043       0.09241030


      Level of          Level of                 --------BulkDens5Depth-------
      Farm              SlopePosition      N             Mean          Std Dev

      Kaalfontein       Bottom            11       1.00972727       0.04518870
      Kaalfontein       Mid               11       1.01745455       0.04558150
      Kaalfontein       Top               11       1.00890909       0.03383033
      Schietfontein     Bottom            12       1.16666667       0.02674232
      Schietfontein     Mid               12       1.18083333       0.02274696
      Schietfontein     Top               12       1.17416667       0.04231018

  Gilbert Pule: Experiment 2 data for statistical analysisNo Outliers 5cm only.SAS 44
                             Project number = 000971-Y5   08:36 Friday, March 9, 2018

                                  The GLM Procedure

                          t Tests (LSD) for BulkDens5Depth

       NOTE: This test controls the Type I comparisonwise error rate, not the
                             experimentwise error rate.


                        Alpha                            0.05
                        Error Degrees of Freedom           63
                        Error Mean Square            0.001363
                        Critical Value of t           1.99834
                        Least Significant Difference   0.0178
                        Harmonic Mean of Cell Sizes  34.43478

                           NOTE: Cell sizes are not equal.


             Means with the same letter are not significantly different.


              t Grouping          Mean      N    Farm

                       A      1.173889     36    Schietfontein

                       B      1.012030     33    Kaalfontein

  Gilbert Pule: Experiment 2 data for statistical analysisNo Outliers 5cm only.SAS 45
                             Project number = 000971-Y5   08:36 Friday, March 9, 2018

                                  The GLM Procedure

                          t Tests (LSD) for BulkDens5Depth

       NOTE: This test controls the Type I comparisonwise error rate, not the
                             experimentwise error rate.


                        Alpha                            0.05
                        Error Degrees of Freedom           63
                        Error Mean Square            0.001363
                        Critical Value of t           1.99834
                        Least Significant Difference   0.0218


             Means with the same letter are not significantly different.


                                                   Slope
                t Grouping          Mean      N    Position

                         A       1.10270     23    Mid
                         A
                         A       1.09513     23    Top
                         A
                         A       1.09161     23    Bottom

  Gilbert Pule: Experiment 2 data for statistical analysisNo Outliers 5cm only.SAS 46
                             Project number = 000971-Y5   08:36 Friday, March 9, 2018

                                  The GLM Procedure

                           Dependent Variable: Na5Dpth

                                         Sum of
 Source                      DF         Squares     Mean Square    F Value    Pr > F

 Model                        5     179.0531742      35.8106348      18.70    <.0001

 Error                       62     118.7512729       1.9153431

 Corrected Total             67     297.8044471


                R-Square     Coeff Var      Root MSE    Na5Dpth Mean

                0.601244      61.67053      1.383959        2.244118


 Source                      DF       Type I SS     Mean Square    F Value    Pr > F

 Farm                         1     153.5177485     153.5177485      80.15    <.0001
 SlopePosition                2       9.1320750       4.5660375       2.38    0.1006
 Farm*SlopePosition           2      16.4033507       8.2016753       4.28    0.0181

  Gilbert Pule: Experiment 2 data for statistical analysisNo Outliers 5cm only.SAS 47
                             Project number = 000971-Y5   08:36 Friday, March 9, 2018

                                  The GLM Procedure

               Level of                 -----------Na5Dpth-----------
               Farm               N             Mean          Std Dev

               Kaalfontein       33       3.79151515       1.71833881
               Schietfontein     35       0.78514286       1.21025853


               Level of                 -----------Na5Dpth-----------
               SlopePosition      N             Mean          Std Dev

               Bottom            24       2.78083333       2.73115029
               Mid               21       2.00523810       1.79183877
               Top               23       1.90217391       1.52596305


      Level of          Level of                 -----------Na5Dpth-----------
      Farm              SlopePosition      N             Mean          Std Dev

      Kaalfontein       Bottom            12       4.92750000       2.10710972
      Kaalfontein       Mid               10       3.33300000       1.10929457
      Kaalfontein       Top               11       2.96909091       0.97693863
      Schietfontein     Bottom            12       0.63416667       1.04993037
      Schietfontein     Mid               11       0.79818182       1.39579954
      Schietfontein     Top               12       0.92416667       1.26795797

  Gilbert Pule: Experiment 2 data for statistical analysisNo Outliers 5cm only.SAS 48
                             Project number = 000971-Y5   08:36 Friday, March 9, 2018

                                  The GLM Procedure

                              t Tests (LSD) for Na5Dpth

       NOTE: This test controls the Type I comparisonwise error rate, not the
                             experimentwise error rate.


                        Alpha                            0.05
                        Error Degrees of Freedom           62
                        Error Mean Square            1.915343
                        Critical Value of t           1.99897
                        Least Significant Difference   0.6713
                        Harmonic Mean of Cell Sizes  33.97059

                           NOTE: Cell sizes are not equal.


             Means with the same letter are not significantly different.


              t Grouping          Mean      N    Farm

                       A        3.7915     33    Kaalfontein

                       B        0.7851     35    Schietfontein

  Gilbert Pule: Experiment 2 data for statistical analysisNo Outliers 5cm only.SAS 49
                             Project number = 000971-Y5   08:36 Friday, March 9, 2018

                                  The GLM Procedure

                              t Tests (LSD) for Na5Dpth

       NOTE: This test controls the Type I comparisonwise error rate, not the
                             experimentwise error rate.


                        Alpha                            0.05
                        Error Degrees of Freedom           62
                        Error Mean Square            1.915343
                        Critical Value of t           1.99897
                        Least Significant Difference    0.823
                        Harmonic Mean of Cell Sizes  22.59649

                           NOTE: Cell sizes are not equal.


             Means with the same letter are not significantly different.


                                                      Slope
                   t Grouping          Mean      N    Position

                            A        2.7808     24    Bottom
                            A
                       B    A        2.0052     21    Mid
                       B
                       B             1.9022     23    Top

  Gilbert Pule: Experiment 2 data for statistical analysisNo Outliers 5cm only.SAS 50
                             Project number = 000971-Y5   08:36 Friday, March 9, 2018

                                  The GLM Procedure

                           Dependent Variable: Ca5Depth

                                         Sum of
 Source                      DF         Squares     Mean Square    F Value    Pr > F

 Model                        5     296506.8404      59301.3681      21.16    <.0001

 Error                       65     182145.7799       2802.2428

 Corrected Total             70     478652.6202


                R-Square     Coeff Var      Root MSE    Ca5Depth Mean

                0.619461      73.75524      52.93621         71.77282


 Source                      DF       Type I SS     Mean Square    F Value    Pr > F

 Farm                         1     293393.9165     293393.9165     104.70    <.0001
 SlopePosition                2       2507.4531       1253.7266       0.45    0.6412
 Farm*SlopePosition           2        605.4708        302.7354       0.11    0.8978

  Gilbert Pule: Experiment 2 data for statistical analysisNo Outliers 5cm only.SAS 51
                             Project number = 000971-Y5   08:36 Friday, March 9, 2018

                                  The GLM Procedure

               Level of                 -----------Ca5Depth----------
               Farm               N             Mean          Std Dev

               Kaalfontein       35       136.967714       68.7690144
               Schietfontein     36         8.388889       26.4395128


               Level of                 -----------Ca5Depth----------
               SlopePosition      N             Mean          Std Dev

               Bottom            24       66.1670833       78.7202839
               Mid               24       71.5570833       81.7784480
               Top               23       77.8473913       90.6298328


      Level of          Level of                 -----------Ca5Depth----------
      Farm              SlopePosition      N             Mean          Std Dev

      Kaalfontein       Bottom            12       126.503333       69.7334810
      Kaalfontein       Mid               12       138.388333       64.4999467
      Kaalfontein       Top               11       146.833636       76.8960696
      Schietfontein     Bottom            12         5.830833       12.3098464
      Schietfontein     Mid               12         4.725833        8.8460412
      Schietfontein     Top               12        14.610000       43.9370167

  Gilbert Pule: Experiment 2 data for statistical analysisNo Outliers 5cm only.SAS 52
                             Project number = 000971-Y5   08:36 Friday, March 9, 2018

                                  The GLM Procedure

                             t Tests (LSD) for Ca5Depth

       NOTE: This test controls the Type I comparisonwise error rate, not the
                             experimentwise error rate.


                        Alpha                            0.05
                        Error Degrees of Freedom           65
                        Error Mean Square            2802.243
                        Critical Value of t           1.99714
                        Least Significant Difference   25.096
                        Harmonic Mean of Cell Sizes  35.49296

                           NOTE: Cell sizes are not equal.


             Means with the same letter are not significantly different.


              t Grouping          Mean      N    Farm

                       A        136.97     35    Kaalfontein

                       B          8.39     36    Schietfontein

  Gilbert Pule: Experiment 2 data for statistical analysisNo Outliers 5cm only.SAS 53
                             Project number = 000971-Y5   08:36 Friday, March 9, 2018

                                  The GLM Procedure

                             t Tests (LSD) for Ca5Depth

       NOTE: This test controls the Type I comparisonwise error rate, not the
                             experimentwise error rate.


                        Alpha                            0.05
                        Error Degrees of Freedom           65
                        Error Mean Square            2802.243
                        Critical Value of t           1.99714
                        Least Significant Difference   30.739
                        Harmonic Mean of Cell Sizes  23.65714

                           NOTE: Cell sizes are not equal.


             Means with the same letter are not significantly different.


                                                   Slope
                t Grouping          Mean      N    Position

                         A         77.85     23    Top
                         A
                         A         71.56     24    Mid
                         A
                         A         66.17     24    Bottom

  Gilbert Pule: Experiment 2 data for statistical analysisNo Outliers 5cm only.SAS 54
                             Project number = 000971-Y5   08:36 Friday, March 9, 2018

                                  The GLM Procedure

                           Dependent Variable: Mg5Depth

                                         Sum of
 Source                      DF         Squares     Mean Square    F Value    Pr > F

 Model                        5     20508.67550      4101.73510      11.64    <.0001

 Error                       64     22554.00868       352.40639

 Corrected Total             69     43062.68418


                R-Square     Coeff Var      Root MSE    Mg5Depth Mean

                0.476252      58.76214      18.77249         31.94657


 Source                      DF       Type I SS     Mean Square    F Value    Pr > F

 Farm                         1     19549.38724     19549.38724      55.47    <.0001
 SlopePosition                2       397.64445       198.82222       0.56    0.5716
 Farm*SlopePosition           2       561.64381       280.82190       0.80    0.4552

  Gilbert Pule: Experiment 2 data for statistical analysisNo Outliers 5cm only.SAS 55
                             Project number = 000971-Y5   08:36 Friday, March 9, 2018

                                  The GLM Procedure

               Level of                 -----------Mg5Depth----------
               Farm               N             Mean          Std Dev

               Kaalfontein       34       49.1426471       24.8012395
               Schietfontein     36       15.7058333        9.5841358


               Level of                 -----------Mg5Depth----------
               SlopePosition      N             Mean          Std Dev

               Bottom            23       28.2995652       21.2495847
               Mid               24       33.8783333       26.1174344
               Top               23       33.5778261       27.7842745


      Level of          Level of                 -----------Mg5Depth----------
      Farm              SlopePosition      N             Mean          Std Dev

      Kaalfontein       Bottom            11       41.5509091       24.2142778
      Kaalfontein       Mid               12       51.9683333       24.8224121
      Kaalfontein       Top               11       53.6518182       25.8919110
      Schietfontein     Bottom            12       16.1525000        5.7877663
      Schietfontein     Mid               12       15.7883333        9.8032498
      Schietfontein     Top               12       15.1766667       12.7332445

  Gilbert Pule: Experiment 2 data for statistical analysisNo Outliers 5cm only.SAS 56
                             Project number = 000971-Y5   08:36 Friday, March 9, 2018

                                  The GLM Procedure

                             t Tests (LSD) for Mg5Depth

       NOTE: This test controls the Type I comparisonwise error rate, not the
                             experimentwise error rate.


                        Alpha                            0.05
                        Error Degrees of Freedom           64
                        Error Mean Square            352.4064
                        Critical Value of t           1.99773
                        Least Significant Difference   8.9684
                        Harmonic Mean of Cell Sizes  34.97143

                           NOTE: Cell sizes are not equal.


             Means with the same letter are not significantly different.


              t Grouping          Mean      N    Farm

                       A        49.143     34    Kaalfontein

                       B        15.706     36    Schietfontein

  Gilbert Pule: Experiment 2 data for statistical analysisNo Outliers 5cm only.SAS 57
                             Project number = 000971-Y5   08:36 Friday, March 9, 2018

                                  The GLM Procedure

                             t Tests (LSD) for Mg5Depth

       NOTE: This test controls the Type I comparisonwise error rate, not the
                             experimentwise error rate.


                        Alpha                            0.05
                        Error Degrees of Freedom           64
                        Error Mean Square            352.4064
                        Critical Value of t           1.99773
                        Least Significant Difference   10.982
                        Harmonic Mean of Cell Sizes  23.32394

                           NOTE: Cell sizes are not equal.


             Means with the same letter are not significantly different.


                                                   Slope
                t Grouping          Mean      N    Position

                         A        33.878     24    Mid
                         A
                         A        33.578     23    Top
                         A
                         A        28.300     23    Bottom

  Gilbert Pule: Experiment 2 data for statistical analysisNo Outliers 5cm only.SAS 58
                             Project number = 000971-Y5   08:36 Friday, March 9, 2018

                                  The GLM Procedure

                         Dependent Variable: TotalN5Depth

                                         Sum of
 Source                      DF         Squares     Mean Square    F Value    Pr > F

 Model                        5      0.00330775      0.00066155       8.60    <.0001

 Error                       65      0.00499735      0.00007688

 Corrected Total             70      0.00830510


              R-Square     Coeff Var      Root MSE    TotalN5Depth Mean

              0.398279      14.83312      0.008768             0.059113


 Source                      DF       Type I SS     Mean Square    F Value    Pr > F

 Farm                         1      0.00280077      0.00280077      36.43    <.0001
 SlopePosition                2      0.00027088      0.00013544       1.76    0.1799
 Farm*SlopePosition           2      0.00023610      0.00011805       1.54    0.2231

  Gilbert Pule: Experiment 2 data for statistical analysisNo Outliers 5cm only.SAS 59
                             Project number = 000971-Y5   08:36 Friday, March 9, 2018

                                  The GLM Procedure

               Level of                 ---------TotalN5Depth--------
               Farm               N             Mean          Std Dev

               Kaalfontein       36       0.06530556       0.01074861
               Schietfontein     35       0.05274286       0.00655449


               Level of                 ---------TotalN5Depth--------
               SlopePosition      N             Mean          Std Dev

               Bottom            24       0.06175000       0.00960638
               Mid               24       0.05745833       0.01184677
               Top               23       0.05808696       0.01107377


      Level of          Level of                 ---------TotalN5Depth--------
      Farm              SlopePosition      N             Mean          Std Dev

      Kaalfontein       Bottom            12       0.06550000       0.01069834
      Kaalfontein       Mid               12       0.06516667       0.01173056
      Kaalfontein       Top               12       0.06525000       0.01074604
      Schietfontein     Bottom            12       0.05800000       0.00691507
      Schietfontein     Mid               12       0.04975000       0.00511904
      Schietfontein     Top               11       0.05027273       0.00374409

  Gilbert Pule: Experiment 2 data for statistical analysisNo Outliers 5cm only.SAS 60
                             Project number = 000971-Y5   08:36 Friday, March 9, 2018

                                  The GLM Procedure

                           t Tests (LSD) for TotalN5Depth

       NOTE: This test controls the Type I comparisonwise error rate, not the
                             experimentwise error rate.


                        Alpha                            0.05
                        Error Degrees of Freedom           65
                        Error Mean Square            0.000077
                        Critical Value of t           1.99714
                        Least Significant Difference   0.0042
                        Harmonic Mean of Cell Sizes  35.49296

                           NOTE: Cell sizes are not equal.


             Means with the same letter are not significantly different.


              t Grouping          Mean      N    Farm

                       A      0.065306     36    Kaalfontein

                       B      0.052743     35    Schietfontein

  Gilbert Pule: Experiment 2 data for statistical analysisNo Outliers 5cm only.SAS 61
                             Project number = 000971-Y5   08:36 Friday, March 9, 2018

                                  The GLM Procedure

                           t Tests (LSD) for TotalN5Depth

       NOTE: This test controls the Type I comparisonwise error rate, not the
                             experimentwise error rate.


                        Alpha                            0.05
                        Error Degrees of Freedom           65
                        Error Mean Square            0.000077
                        Critical Value of t           1.99714
                        Least Significant Difference   0.0051
                        Harmonic Mean of Cell Sizes  23.65714

                           NOTE: Cell sizes are not equal.


             Means with the same letter are not significantly different.


                                                   Slope
                t Grouping          Mean      N    Position

                         A      0.061750     24    Bottom
                         A
                         A      0.058087     23    Top
                         A
                         A      0.057458     24    Mid

  Gilbert Pule: Experiment 2 data for statistical analysisNo Outliers 5cm only.SAS 62
                             Project number = 000971-Y5   08:36 Friday, March 9, 2018

                              The UNIVARIATE Procedure
                                Variable:  rAltitude

                                       Moments

           N                          69    Sum Weights                 69
           Mean               -0.0007259    Sum Observations    -0.0500852
           Std Deviation      1.01775249    Variance            1.03582013
           Skewness            -0.092108    Kurtosis            -0.6544332
           Uncorrected SS     70.4358054    Corrected SS        70.4357691
           Coeff Variation    -140210.95    Std Error Mean        0.122523


                             Basic Statistical Measures

                   Location                    Variability

               Mean     -0.00073     Std Deviation            1.01775
               Median   -0.05561     Variance                 1.03582
               Mode      0.77527     Range                    4.44920
                                     Interquartile Range      1.50119


                             Tests for Location: Mu0=0

                  Test           -Statistic-    -----p Value------

                  Student's t    t  -0.00592    Pr > |t|    0.9953
                  Sign           M      -1.5    Pr >= |M|   0.8099
                  Signed Rank    S        25    Pr >= |S|   0.8824


                                Tests for Normality

             Test                  --Statistic---    -----p Value------

             Shapiro-Wilk          W     0.985016    Pr < W      0.5797
             Kolmogorov-Smirnov    D     0.082442    Pr > D     >0.1500
             Cramer-von Mises      W-Sq  0.052955    Pr > W-Sq  >0.2500
             Anderson-Darling      A-Sq  0.326397    Pr > A-Sq  >0.2500


                              Quantiles (Definition 5)

                              Level           Quantile

                              100% Max       2.2557987
                              99%            2.2557987
                              95%            1.5293683
                              90%            1.3347374
                              75% Q3         0.7752737
                              50% Median    -0.0556055
                              25% Q1        -0.7259192
                              10%           -1.3734634
                              5%            -1.7251868
                              1%            -2.1934057
                              0% Min        -2.1934057


                                Extreme Observations

                     ------Lowest-----        -----Highest-----

                        Value      Obs           Value      Obs

                     -2.19341       12         1.47071        5
                     -1.96722       52         1.52937       36
                     -1.88689       43         1.52937       39
                     -1.72519       56         1.80655       20
                     -1.64744       35         2.25580       40


                                    Missing Values

                                            -----Percent Of-----
                     Missing                             Missing
                       Value       Count     All Obs         Obs

                           .           3        4.17      100.00


                  Stem Leaf                     #             Boxplot
                    22 6                        1                |
                    20                                           |
                    18 1                        1                |
                    16                                           |
                    14 7733                     4                |
                    12 40003                    5                |
                    10                                           |
                     8 4488                     4                |
                     6 111158888                9             +-----+
                     4 356                      3             |     |
                     2 339                      3             |     |
                     0 117                      3             |     |
                    -0 17766                    5             *--+--*
                    -2 5533888                  7             |     |
                    -4 20006                    5             |     |
                    -6 439                      3             +-----+
                    -8 5510                     4                |
                   -10 84                       2                |
                   -12 7740                     4                |
                   -14 4                        1                |
                   -16 35                       2                |
                   -18 79                       2                |
                   -20 9                        1                |
                       ----+----+----+----+
                   Multiply Stem.Leaf by 10**-1


                                   Normal Probability Plot
                 2.3+                                               ++*
                    |                                             ++
                    |                                           ++*
                    |                                         ++
                    |                                       *** *
                    |                                   ****
                    |                                   ++
                    |                                 ***
                    |                             *****
                    |                            **+
                    |                           **
                 0.1+                          *
                    |                        **
                    |                     ****
                    |                   ***
                    |                 ***
                    |                **
                    |              +*
                    |            ***
                    |          +*
                    |        +**
                    |     *+*
                -2.1+ *  ++
                     +----+----+----+----+----+----+----+----+----+----+
                         -2        -1         0        +1        +2


  Gilbert Pule: Experiment 2 data for statistical analysisNo Outliers 5cm only.SAS 63
                             Project number = 000971-Y5   08:36 Friday, March 9, 2018

                              The UNIVARIATE Procedure
                                Variable:  rLongitude

                                       Moments

           N                          70    Sum Weights                 70
           Mean               0.07517031    Sum Observations    5.26192187
           Std Deviation       1.5356454    Variance             2.3582068
           Skewness            6.4505509    Kurtosis            48.0230827
           Uncorrected SS     163.111809    Corrected SS        162.716269
           Coeff Variation    2042.88815    Std Error Mean      0.18354473


                             Basic Statistical Measures

                   Location                    Variability

               Mean      0.07517     Std Deviation            1.53565
               Median   -0.02663     Variance                 2.35821
               Mode     -0.77474     Range                   13.08965
                                     Interquartile Range      0.41042

       Note: The mode displayed is the smallest of 2 modes with a count of 2.


                             Tests for Location: Mu0=0

                  Test           -Statistic-    -----p Value------

                  Student's t    t  0.409548    Pr > |t|    0.6834
                  Sign           M        -4    Pr >= |M|   0.4030
                  Signed Rank    S    -234.5    Pr >= |S|   0.1717


                                Tests for Normality

             Test                  --Statistic---    -----p Value------

             Shapiro-Wilk          W      0.38692    Pr < W     <0.0001
             Kolmogorov-Smirnov    D     0.335798    Pr > D     <0.0100
             Cramer-von Mises      W-Sq  2.303616    Pr > W-Sq  <0.0050
             Anderson-Darling      A-Sq  11.94664    Pr > A-Sq  <0.0050


                              Quantiles (Definition 5)

                              Level            Quantile

                              100% Max       11.6348512
                              99%            11.6348512
                              95%             0.6403774
                              90%             0.3527893
                              75% Q3          0.1018347
                              50% Median     -0.0266261
                              25% Q1         -0.3085903
                              10%            -0.7747377
                              5%             -1.0825239
                              1%             -1.4548030
                              0% Min         -1.4548030


                                Extreme Observations

                    ------Lowest-----        ------Highest------

                       Value      Obs             Value      Obs

                    -1.45480        2          0.553288       20
                    -1.40046       11          0.640377       19
                    -1.12251        3          1.203493        6
                    -1.08252       12          3.369923       25
                    -1.00352       26         11.634851        1


                                    Missing Values

                                            -----Percent Of-----
                     Missing                             Missing
                       Value       Count     All Obs         Obs

                           .           2        2.78      100.00


           Stem Leaf                                   #             Boxplot
             11 6                                      1                *
             10
              9
              8
              7
              6
              5
              4
              3 4                                      1                *
              2
              1 2                                      1                0
              0 0000001111111111222333334566          28             +--+--+
             -0 9887776665543322211111111100000000    34             *-----*
             -1 54110                                  5                0
                ----+----+----+----+----+----+----


                                   Normal Probability Plot
                11.5+                                                 *
                    |
                    |
                    |
                    |
                    |
                    |
                    |
                    |                                             *+++++
                    |                                      +++++++
                    |                                ++++++     *
                    |                         ++***************
                    |           ****************
                -1.5+ *   * * ** ++++++
                     +----+----+----+----+----+----+----+----+----+----+
                         -2        -1         0        +1        +2


  Gilbert Pule: Experiment 2 data for statistical analysisNo Outliers 5cm only.SAS 64
                             Project number = 000971-Y5   08:36 Friday, March 9, 2018

                              The UNIVARIATE Procedure
                                Variable:  rLatitude

                                       Moments

           N                          70    Sum Weights                 70
           Mean               -0.0015813    Sum Observations    -0.1106932
           Std Deviation      1.01674858    Variance            1.03377767
           Skewness           -0.2032267    Kurtosis            -0.8265197
           Uncorrected SS     71.3308342    Corrected SS        71.3306592
           Coeff Variation     -64296.99    Std Error Mean       0.1215247


                             Basic Statistical Measures

                   Location                    Variability

               Mean     -0.00158     Std Deviation            1.01675
               Median    0.11167     Variance                 1.03378
               Mode       .          Range                    3.83457
                                     Interquartile Range      1.51268


                             Tests for Location: Mu0=0

                  Test           -Statistic-    -----p Value------

                  Student's t    t  -0.01301    Pr > |t|    0.9897
                  Sign           M         2    Pr >= |M|   0.7202
                  Signed Rank    S      17.5    Pr >= |S|   0.9193


                                Tests for Normality

             Test                  --Statistic---    -----p Value------

             Shapiro-Wilk          W     0.972591    Pr < W      0.1277
             Kolmogorov-Smirnov    D     0.073348    Pr > D     >0.1500
             Cramer-von Mises      W-Sq  0.061517    Pr > W-Sq  >0.2500
             Anderson-Darling      A-Sq  0.445995    Pr > A-Sq  >0.2500


                               Quantiles (Definition 5)

                               Level          Quantile

                               100% Max       1.875308
                               99%            1.875308
                               95%            1.619144
                               90%            1.237225
                               75% Q3         0.780731
                               50% Median     0.111671
                               25% Q1        -0.731953
                               10%           -1.511795
                               5%            -1.822551
                               1%            -1.959259
                               0% Min        -1.959259


                                Extreme Observations

                     ------Lowest-----        -----Highest-----

                        Value      Obs           Value      Obs

                     -1.95926       37         1.52052       47
                     -1.93881       62         1.61914       59
                     -1.91483       49         1.63892       72
                     -1.82255       23         1.82491       60
                     -1.57745       50         1.87531       48


                                    Missing Values

                                            -----Percent Of-----
                     Missing                             Missing
                       Value       Count     All Obs         Obs

                           .           2        2.78      100.00


                  Stem Leaf                     #             Boxplot
                    18 28                       2                |
                    16 24                       2                |
                    14 2                        1                |
                    12 83                       2                |
                    10 041269                   6                |
                     8 4778                     4                |
                     6 39168                    5             +-----+
                     4 2233449                  7             |     |
                     2 73                       2             |     |
                     0 084778                   6             *-----*
                    -0 977317                   6             |  +  |
                    -2 50                       2             |     |
                    -4 22093                    5             |     |
                    -6 363                      3             +-----+
                    -8 5872                     4                |
                   -10                                           |
                   -12 0741                     4                |
                   -14 87115                    5                |
                   -16                                           |
                   -18 6412                     4                |
                       ----+----+----+----+
                   Multiply Stem.Leaf by 10**-1


                                   Normal Probability Plot
                 1.9+                                           ++*   *
                    |                                         *+*
                    |                                       +*
                    |                                     +**
                    |                                  ****
                    |                                ***
                    |                              ***
                    |                           ****
                    |                           *+
                    |                        ***
                    |                      ***+
                    |                      *+
                    |                    **
                    |                  **
                    |                ***
                    |              ++
                    |            ++***
                    |          ****
                    |        ++
                -1.9+ *   *+* *
                     +----+----+----+----+----+----+----+----+----+----+
                         -2        -1         0        +1        +2


  Gilbert Pule: Experiment 2 data for statistical analysisNo Outliers 5cm only.SAS 65
                             Project number = 000971-Y5   08:36 Friday, March 9, 2018

                              The UNIVARIATE Procedure
                                Variable:  rSplumDens

                                       Moments

           N                          72    Sum Weights                 72
           Mean               0.00441632    Sum Observations    0.31797473
           Std Deviation      1.03619357    Variance            1.07369711
           Skewness           0.56773498    Kurtosis            1.99962945
           Uncorrected SS     76.2338989    Corrected SS        76.2324947
           Coeff Variation    23462.8509    Std Error Mean      0.12211658


                             Basic Statistical Measures

                   Location                    Variability

               Mean      0.00442     Std Deviation            1.03619
               Median   -0.06632     Variance                 1.07370
               Mode     -0.28440     Range                    6.42512
                                     Interquartile Range      1.08267


                             Tests for Location: Mu0=0

                  Test           -Statistic-    -----p Value------

                  Student's t    t  0.036165    Pr > |t|    0.9713
                  Sign           M      -3.5    Pr >= |M|   0.4767
                  Signed Rank    S    -128.5    Pr >= |S|   0.4654


                                Tests for Normality

             Test                  --Statistic---    -----p Value------

             Shapiro-Wilk          W     0.939125    Pr < W      0.0017
             Kolmogorov-Smirnov    D     0.111459    Pr > D      0.0248
             Cramer-von Mises      W-Sq  0.221514    Pr > W-Sq  <0.0050
             Anderson-Darling      A-Sq  1.491302    Pr > A-Sq  <0.0050


                              Quantiles (Definition 5)

                              Level           Quantile

                              100% Max       3.2480115
                              99%            3.2480115
                              95%            2.2473264
                              90%            0.9201715
                              75% Q3         0.4507162
                              50% Median    -0.0663199
                              25% Q1        -0.6319515
                              10%           -1.0266742
                              5%            -1.2609702
                              1%            -3.1771054
                              0% Min        -3.1771054


                                Extreme Observations

                     ------Lowest-----        -----Highest-----

                        Value      Obs           Value      Obs

                     -3.17711       40         1.99073       70
                     -1.92878       41         2.24733       49
                     -1.44370       43         2.42374       27
                     -1.26097       57         2.48325       15
                     -1.10446       18         3.24801       37


                  Stem Leaf                     #             Boxplot
                     3 2                        1                0
                     2 5                        1                0
                     2 0024                     4                0
                     1 7                        1                |
                     1                                           |
                     0 55666778899             11             +-----+
                     0 001122222233344         15             |  +  |
                    -0 44433333332211110       17             *-----*
                    -0 99877766655             11             +-----+
                    -1 431000000                9                |
                    -1 9                        1                |
                    -2
                    -2
                    -3 2                        1                0
                       ----+----+----+----+


                                   Normal Probability Plot
                3.25+                                                 *
                    |                                                  +
                    |                                         * * *++++
                    |                                      ***++++
                    |                                   +++++
                    |                              ++*******
                    |                         +*******
                    |                   ********
                    |              ******+
                    |       * *****++
                    |     *+++++
                    | +++++
                    |+
               -3.25+ *
                     +----+----+----+----+----+----+----+----+----+----+
                         -2        -1         0        +1        +2


  Gilbert Pule: Experiment 2 data for statistical analysisNo Outliers 5cm only.SAS 66
                             Project number = 000971-Y5   08:36 Friday, March 9, 2018

                              The UNIVARIATE Procedure
                             Variable:  rSplumMeanConCov

                                       Moments

           N                          72    Sum Weights                 72
           Mean               0.00503411    Sum Observations    0.36245576
           Std Deviation      1.03708123    Variance            1.07553747
           Skewness           0.69423368    Kurtosis            2.15423374
           Uncorrected SS     76.3649853    Corrected SS        76.3631607
           Coeff Variation     20601.093    Std Error Mean      0.12222119


                             Basic Statistical Measures

                   Location                    Variability

               Mean      0.00503     Std Deviation            1.03708
               Median   -0.09888     Variance                 1.07554
               Mode     -0.71987     Range                    5.71627
                                     Interquartile Range      0.98644

       Note: The mode displayed is the smallest of 2 modes with a count of 2.


                             Tests for Location: Mu0=0

                  Test           -Statistic-    -----p Value------

                  Student's t    t  0.041189    Pr > |t|    0.9673
                  Sign           M        -4    Pr >= |M|   0.4096
                  Signed Rank    S       -81    Pr >= |S|   0.6526


                                Tests for Normality

             Test                  --Statistic---    -----p Value------

             Shapiro-Wilk          W     0.940729    Pr < W      0.0021
             Kolmogorov-Smirnov    D     0.106724    Pr > D      0.0416
             Cramer-von Mises      W-Sq  0.192688    Pr > W-Sq   0.0065
             Anderson-Darling      A-Sq  1.220308    Pr > A-Sq  <0.0050


                              Quantiles (Definition 5)

                              Level           Quantile

                              100% Max       3.5783282
                              99%            3.5783282
                              95%            1.5717623
                              90%            1.1325783
                              75% Q3         0.4657042
                              50% Median    -0.0988804
                              25% Q1        -0.5207367
                              10%           -0.8705845
                              5%            -2.0460618
                              1%            -2.1379432
                              0% Min        -2.1379432


                                Extreme Observations

                     ------Lowest-----        -----Highest-----

                        Value      Obs           Value      Obs

                     -2.13794        5         1.45822       55
                     -2.08791       28         1.57176       17
                     -2.05303       15         2.65924       31
                     -2.04606       58         2.85165       32
                     -1.81172       27         3.57833       40


                  Stem Leaf                     #             Boxplot
                     3 6                        1                *
                     3
                     2 79                       2                0
                     2
                     1 56                       2                |
                     1 01124                    5                |
                     0 556777889                9             +-----+
                     0 0111222223333           13             |  +  |
                    -0 4443333332222111100     19             *-----*
                    -0 98888777765555          14             +-----+
                    -1 0                        1                |
                    -1 85                       2                |
                    -2 1110                     4                0
                       ----+----+----+----+


                                   Normal Probability Plot
                3.75+                                                 *
                    |
                    |                                           * *    +
                    |                                             +++++
                    |                                        +*+++
                    |                                   ++****
                0.75+                              ++*****
                    |                         ++******
                    |                  *********
                    |            *******++
                    |           +++++
                    |      ++++**
               -2.25+ *+++* * *
                     +----+----+----+----+----+----+----+----+----+----+
                         -2        -1         0        +1        +2


  Gilbert Pule: Experiment 2 data for statistical analysisNo Outliers 5cm only.SAS 67
                             Project number = 000971-Y5   08:36 Friday, March 9, 2018

                              The UNIVARIATE Procedure
                              Variable:  rIndivIntercep

                                       Moments

           N                          72    Sum Weights                 72
           Mean               -0.0006948    Sum Observations     -0.050025
           Std Deviation       1.0298273    Variance            1.06054426
           Skewness           -0.0896214    Kurtosis            1.14891569
           Uncorrected SS     75.2986776    Corrected SS        75.2986428
           Coeff Variation    -148221.11    Std Error Mean      0.12136631


                             Basic Statistical Measures

                   Location                    Variability

               Mean     -0.00069     Std Deviation            1.02983
               Median    0.00000     Variance                 1.06054
               Mode     -1.03757     Range                    5.83751
                                     Interquartile Range      1.30493


                             Tests for Location: Mu0=0

                  Test           -Statistic-    -----p Value------

                  Student's t    t  -0.00572    Pr > |t|    0.9954
                  Sign           M         0    Pr >= |M|   1.0000
                  Signed Rank    S      20.5    Pr >= |S|   0.9055


                                Tests for Normality

             Test                  --Statistic---    -----p Value------

             Shapiro-Wilk          W     0.978966    Pr < W      0.2701
             Kolmogorov-Smirnov    D     0.060476    Pr > D     >0.1500
             Cramer-von Mises      W-Sq  0.042517    Pr > W-Sq  >0.2500
             Anderson-Darling      A-Sq  0.398411    Pr > A-Sq  >0.2500


                               Quantiles (Definition 5)

                               Level          Quantile

                               100% Max       2.987529
                               99%            2.987529
                               95%            1.467281
                               90%            1.110942
                               75% Q3         0.688550
                               50% Median     0.000000
                               25% Q1        -0.616382
                               10%           -1.037566
                               5%            -1.572713
                               1%            -2.849985
                               0% Min        -2.849985


                                Extreme Observations

                     ------Lowest-----        -----Highest-----

                        Value      Obs           Value      Obs

                     -2.84999       46         1.39242       61
                     -2.64770       40         1.46728       27
                     -2.32016       58         1.63331       42
                     -1.57271        5         2.51533       49
                     -1.31790       32         2.98753       37


                  Stem Leaf                     #             Boxplot
                     3 0                        1                0
                     2 5                        1                |
                     2                                           |
                     1 56                       2                |
                     1 11234                    5                |
                     0 55677788899999          14             +-----+
                     0 00122223333344          14             *-----*
                    -0 44333332111             11             |  +  |
                    -0 987776655555            12             +-----+
                    -1 33100000                 8                |
                    -1 6                        1                |
                    -2 3                        1                |
                    -2 86                       2                0
                       ----+----+----+----+


                                   Normal Probability Plot
                3.25+
                    |                                             *   *+
                    |                                             +++++
                    |                                        +++*+
                    |                                   ++*****
                    |                              *******
                0.25+                         *****
                    |                    *****+
                    |               ******
                    |          ******
                    |      +++*+
                    | +++++ *
               -2.75++*   *
                     +----+----+----+----+----+----+----+----+----+----+
                         -2        -1         0        +1        +2


  Gilbert Pule: Experiment 2 data for statistical analysisNo Outliers 5cm only.SAS 68
                             Project number = 000971-Y5   08:36 Friday, March 9, 2018

                              The UNIVARIATE Procedure
                             Variable:  rBulkDens5Depth

                                       Moments

           N                          69    Sum Weights                 69
           Mean               -0.0033993    Sum Observations      -0.23455
           Std Deviation      1.04604406    Variance            1.09420817
           Skewness           -0.4360441    Kurtosis            3.17299216
           Uncorrected SS     74.4069527    Corrected SS        74.4061554
           Coeff Variation    -30772.555    Std Error Mean      0.12592891


                             Basic Statistical Measures

                   Location                    Variability

               Mean     -0.00340     Std Deviation            1.04604
               Median    0.06404     Variance                 1.09421
               Mode     -0.02338     Range                    7.34024
                                     Interquartile Range      1.26207


                             Tests for Location: Mu0=0

                  Test           -Statistic-    -----p Value------

                  Student's t    t  -0.02699    Pr > |t|    0.9785
                  Sign           M       1.5    Pr >= |M|   0.8099
                  Signed Rank    S      65.5    Pr >= |S|   0.6983


                                Tests for Normality

             Test                  --Statistic---    -----p Value------

             Shapiro-Wilk          W     0.943957    Pr < W      0.0038
             Kolmogorov-Smirnov    D     0.101877    Pr > D      0.0759
             Cramer-von Mises      W-Sq  0.139732    Pr > W-Sq   0.0332
             Anderson-Darling      A-Sq  0.946031    Pr > A-Sq   0.0172


                              Quantiles (Definition 5)

                              Level           Quantile

                              100% Max       3.4547880
                              99%            3.4547880
                              95%            1.1547261
                              90%            1.1099500
                              75% Q3         0.7280196
                              50% Median     0.0640439
                              25% Q1        -0.5340520
                              10%           -1.3280423
                              5%            -1.8665591
                              1%            -3.8854504
                              0% Min        -3.8854504


                                Extreme Observations

                     ------Lowest-----        -----Highest-----

                        Value      Obs           Value      Obs

                     -3.88545       64         1.11246       29
                     -2.12249        4         1.15473       20
                     -1.92789       24         1.17060       35
                     -1.86656       22         2.02335       19
                     -1.72833       32         3.45479        3


                                    Missing Values

                                            -----Percent Of-----
                     Missing                             Missing
                       Value       Count     All Obs         Obs

                           .           3        4.17      100.00


                  Stem Leaf                     #             Boxplot
                     3 5                        1                0
                     3
                     2
                     2 0                        1                |
                     1                                           |
                     1 0011122                  7                |
                     0 5557778888999           13             +-----+
                     0 01111222223344          14             *-----*
                    -0 444333221100000         15             |  +  |
                    -0 998877765                9             +-----+
                    -1 4320                     4                |
                    -1 997                      3                |
                    -2 1                        1                |
                    -2
                    -3
                    -3 9                        1                0
                       ----+----+----+----+


                                   Normal Probability Plot
                3.75+
                    |                                                 *
                    |                                                 ++
                2.25+                                             *+++
                    |                                        +++++
                    |                                   ++***** *
                0.75+                              *******
                    |                         *****
                    |                   ******+
               -0.75+              *****++
                    |           ***++
                    |      +*+**
               -2.25+  +++*
                    |++
                    |
               -3.75+ *
                     +----+----+----+----+----+----+----+----+----+----+
                         -2        -1         0        +1        +2


  Gilbert Pule: Experiment 2 data for statistical analysisNo Outliers 5cm only.SAS 69
                             Project number = 000971-Y5   08:36 Friday, March 9, 2018

                              The UNIVARIATE Procedure
                                 Variable:  rK5Depth

                                       Moments

           N                          72    Sum Weights                 72
           Mean                0.0064426    Sum Observations    0.46386723
           Std Deviation      1.03180101    Variance            1.06461333
           Skewness           0.86544402    Kurtosis              1.409419
           Uncorrected SS     75.5905351    Corrected SS        75.5875466
           Coeff Variation     16015.288    Std Error Mean      0.12159892


                             Basic Statistical Measures

                   Location                    Variability

               Mean      0.00644     Std Deviation            1.03180
               Median   -0.12275     Variance                 1.06461
               Mode     -0.25649     Range                    5.47051
                                     Interquartile Range      1.01334


                             Tests for Location: Mu0=0

                  Test           -Statistic-    -----p Value------

                  Student's t    t  0.052982    Pr > |t|    0.9579
                  Sign           M        -9    Pr >= |M|   0.0444
                  Signed Rank    S      -136    Pr >= |S|   0.4492


                                Tests for Normality

             Test                  --Statistic---    -----p Value------

             Shapiro-Wilk          W     0.947666    Pr < W      0.0047
             Kolmogorov-Smirnov    D     0.141784    Pr > D     <0.0100
             Cramer-von Mises      W-Sq  0.227393    Pr > W-Sq  <0.0050
             Anderson-Darling      A-Sq  1.282861    Pr > A-Sq  <0.0050


                               Quantiles (Definition 5)

                               Level          Quantile

                               100% Max       3.379056
                               99%            3.379056
                               95%            2.243472
                               90%            1.439973
                               75% Q3         0.444879
                               50% Median    -0.122746
                               25% Q1        -0.568460
                               10%           -1.121685
                               5%            -1.581452
                               1%            -2.091456
                               0% Min        -2.091456


                                Extreme Observations

                     ------Lowest-----        -----Highest-----

                        Value      Obs           Value      Obs

                     -2.09146       11         1.84698        9
                     -1.93069       20         2.24347       28
                     -1.65834       30         2.29399        5
                     -1.58145        7         2.70573        4
                     -1.45396        3         3.37906       43


                 Stem Leaf                       #             Boxplot
                    3 4                          1                0
                    2 7                          1                0
                    2 23                         2                0
                    1 688                        3                |
                    1 34                         2                |
                    0 555667899                  9                |
                    0 222334444                  9             +--+--+
                   -0 4443333332211111111100    22             *-----*
                   -0 98888876665555            14             +-----+
                   -1 2211                       4                |
                   -1 9765                       4                |
                   -2 1                          1                0
                      ----+----+----+----+--


                                   Normal Probability Plot
                3.25+                                                 *
                    |                                             *    +
                    |                                         * * +++++
                    |                                      ***++++
                    |                                   ++**+
                    |                              ++*****
                    |                         +++*****
                    |                   **********
                    |              ******+
                    |          ****++
                    |     *+*+*+
               -2.25+ *++++
                     +----+----+----+----+----+----+----+----+----+----+
                         -2        -1         0        +1        +2


  Gilbert Pule: Experiment 2 data for statistical analysisNo Outliers 5cm only.SAS 70
                             Project number = 000971-Y5   08:36 Friday, March 9, 2018

                              The UNIVARIATE Procedure
                                 Variable:  rNa5Dpth

                                       Moments

           N                          68    Sum Weights                 68
           Mean               0.01096621    Sum Observations    0.74570255
           Std Deviation      1.03543019    Variance            1.07211569
           Skewness           1.35666332    Kurtosis            1.69303803
           Uncorrected SS     71.8399286    Corrected SS         71.831751
           Coeff Variation    9442.00249    Std Error Mean      0.12556435


                             Basic Statistical Measures

                   Location                    Variability

               Mean      0.01097     Std Deviation            1.03543
               Median   -0.38913     Variance                 1.07212
               Mode     -0.47561     Range                    4.92187
                                     Interquartile Range      1.14300


                             Tests for Location: Mu0=0

                  Test           -Statistic-    -----p Value------

                  Student's t    t  0.087335    Pr > |t|    0.9307
                  Sign           M        -7    Pr >= |M|   0.1143
                  Signed Rank    S      -174    Pr >= |S|   0.2910


                                Tests for Normality

             Test                  --Statistic---    -----p Value------

             Shapiro-Wilk          W       0.8646    Pr < W     <0.0001
             Kolmogorov-Smirnov    D     0.174173    Pr > D     <0.0100
             Cramer-von Mises      W-Sq  0.578107    Pr > W-Sq  <0.0050
             Anderson-Darling      A-Sq  3.288924    Pr > A-Sq  <0.0050


                               Quantiles (Definition 5)

                               Level          Quantile

                               100% Max       3.028967
                               99%            3.028967
                               95%            2.574104
                               90%            1.532765
                               75% Q3         0.521577
                               50% Median    -0.389128
                               25% Q1        -0.621425
                               10%           -0.945927
                               5%            -1.117292
                               1%            -1.892902
                               0% Min        -1.892902


                                Extreme Observations

                     ------Lowest-----        -----Highest-----

                        Value      Obs           Value      Obs

                     -1.89290       11         2.03929       46
                     -1.21789        9         2.57410       66
                     -1.21020       14         2.87350       10
                     -1.11729        5         3.00576       56
                     -1.01724        4         3.02897        7


                                    Missing Values

                                            -----Percent Of-----
                     Missing                             Missing
                       Value       Count     All Obs         Obs

                           .           4        5.56      100.00


                  Stem Leaf                     #             Boxplot
                    30 13                       2                0
                    28 7                        1                0
                    26
                    24 7                        1                0
                    22
                    20 4                        1                |
                    18 5                        1                |
                    16                                           |
                    14 3                        1                |
                    12                                           |
                    10 002                      3                |
                     8 65                       2                |
                     6 01289                    5                |
                     4 4                        1             +-----+
                     2 30                       2             |     |
                     0 3467824                  7             |  +  |
                    -0 75                       2             |     |
                    -2 65373                    5             *-----*
                    -4 63888888852             11             |     |
                    -6 719999973100000         15             +-----+
                    -8 752                      3                |
                   -10 22                       2                |
                   -12 21                       2                |
                   -14                                           |
                   -16                                           |
                   -18 9                        1                |
                       ----+----+----+----+
                   Multiply Stem.Leaf by 10**-1


                                   Normal Probability Plot
                 3.1+                                             *   *
                    |                                           *
                    |
                    |                                         *       ++
                    |                                               ++
                 2.1+                                        *    ++
                    |                                       *   ++
                    |                                         ++
                    |                                      *++
                    |                                     ++
                 1.1+                                   +**
                    |                                 ++*
                    |                               +***
                    |                             ++*
                    |                           ++  *
                 0.1+                         ++ ***
                    |                       ++  *
                    |                      +  ***
                    |                    +*****
                    |              ********
                -0.9+           ***  ++
                    |         **   ++
                    |     * *    ++
                    |          ++
                    |        ++
                -1.9+ *    ++
                     +----+----+----+----+----+----+----+----+----+----+
                         -2        -1         0        +1        +2


  Gilbert Pule: Experiment 2 data for statistical analysisNo Outliers 5cm only.SAS 71
                             Project number = 000971-Y5   08:36 Friday, March 9, 2018

                              The UNIVARIATE Procedure
                                Variable:  rCa5Depth

                                       Moments

           N                          71    Sum Weights                 71
           Mean               0.00891414    Sum Observations    0.63290404
           Std Deviation      1.03871018    Variance            1.07891884
           Skewness           1.15455462    Kurtosis             2.0840238
           Uncorrected SS     75.5299607    Corrected SS        75.5243189
           Coeff Variation    11652.3862    Std Error Mean      0.12327222


                             Basic Statistical Measures

                   Location                    Variability

               Mean      0.00891     Std Deviation            1.03871
               Median   -0.11417     Variance                 1.07892
               Mode     -0.28622     Range                    5.12282
                                     Interquartile Range      0.54189

       Note: The mode displayed is the smallest of 3 modes with a count of 9.


                             Tests for Location: Mu0=0

                  Test           -Statistic-    -----p Value------

                  Student's t    t  0.072313    Pr > |t|    0.9426
                  Sign           M     -12.5    Pr >= |M|   0.0041
                  Signed Rank    S      -284    Pr >= |S|   0.1038


                                Tests for Normality

             Test                  --Statistic---    -----p Value------

             Shapiro-Wilk          W     0.872512    Pr < W     <0.0001
             Kolmogorov-Smirnov    D     0.207545    Pr > D     <0.0100
             Cramer-von Mises      W-Sq  0.707242    Pr > W-Sq  <0.0050
             Anderson-Darling      A-Sq  3.618593    Pr > A-Sq  <0.0050


                               Quantiles (Definition 5)

                               Level          Quantile

                               100% Max       3.183886
                               99%            3.183886
                               95%            2.762716
                               90%            1.158900
                               75% Q3         0.239631
                               50% Median    -0.114169
                               25% Q1        -0.302260
                               10%           -0.981487
                               5%            -1.562920
                               1%            -1.938934
                               0% Min        -1.938934


                                Extreme Observations

                     ------Lowest-----        -----Highest-----

                        Value      Obs           Value      Obs

                     -1.93893       20         1.96137       33
                     -1.80252       30         2.76272       24
                     -1.78182       11         2.82972       32
                     -1.56292       31         2.88503       64
                     -1.46211        7         3.18389        4


                                    Missing Values

                                            -----Percent Of-----
                     Missing                             Missing
                       Value       Count     All Obs         Obs

                           .           1        1.39      100.00


                  Stem Leaf                      #             Boxplot
                    30 8                         1                *
                    28 39                        2                *
                    26 6                         1                *
                    24
                    22
                    20
                    18 516                       3                0
                    16
                    14
                    12
                    10 06                        2                0
                     8 1                         1                |
                     6 19                        2                |
                     4 029                       3                |
                     2 0467                      4             +-----+
                     0 2577                      4             |  +  |
                    -0 951111111119999999992    21             *-----*
                    -2 40999999999              11             +-----+
                    -4 1                         1                |
                    -6 4                         1                |
                    -8 8855981                   7                |
                   -10 1                         1                |
                   -12 1                         1                0
                   -14 66                        2                0
                   -16 8                         1                0
                   -18 40                        2                0
                       ----+----+----+----+-
                   Multiply Stem.Leaf by 10**-1


                                   Normal Probability Plot
                 3.1+                                                 *
                    |                                           * *
                    |                                         *        +
                    |                                                 +
                    |                                               ++
                 2.1+                                             ++
                    |                                      ***  ++
                    |                                         ++
                    |                                       ++
                    |                                     ++
                 1.1+                                   ++*
                    |                                 ++ **
                    |                               ++  *
                    |                             ++   *
                    |                           ++   **
                 0.1+                         ++   **
                    |                      ********
                    |                  *****
                    |                 *  ++
                    |                 *++
                -0.9+             ****+
                    |            * ++
                    |           *++
                    |         **+
                    |       *++
                -1.9+ *   *++
                     +----+----+----+----+----+----+----+----+----+----+
                         -2        -1         0        +1        +2


  Gilbert Pule: Experiment 2 data for statistical analysisNo Outliers 5cm only.SAS 72
                             Project number = 000971-Y5   08:36 Friday, March 9, 2018

                              The UNIVARIATE Procedure
                                Variable:  rMg5Depth

                                       Moments

           N                          70    Sum Weights                 70
           Mean               0.00692658    Sum Observations    0.48486079
           Std Deviation      1.03226092    Variance            1.06556261
           Skewness           0.88959384    Kurtosis            1.16255018
           Uncorrected SS     73.5271782    Corrected SS        73.5238198
           Coeff Variation    14902.8889    Std Error Mean      0.12337878


                             Basic Statistical Measures

                   Location                    Variability

               Mean      0.00693     Std Deviation            1.03226
               Median   -0.15678     Variance                 1.06556
               Mode       .          Range                    5.25432
                                     Interquartile Range      0.90125


                             Tests for Location: Mu0=0

                  Test           -Statistic-    -----p Value------

                  Student's t    t  0.056141    Pr > |t|    0.9554
                  Sign           M       -11    Pr >= |M|   0.0115
                  Signed Rank    S    -190.5    Pr >= |S|   0.2680


                                Tests for Normality

             Test                  --Statistic---    -----p Value------

             Shapiro-Wilk          W     0.932795    Pr < W      0.0010
             Kolmogorov-Smirnov    D     0.169892    Pr > D     <0.0100
             Cramer-von Mises      W-Sq  0.354067    Pr > W-Sq  <0.0050
             Anderson-Darling      A-Sq  1.844877    Pr > A-Sq  <0.0050


                               Quantiles (Definition 5)

                               Level          Quantile

                               100% Max       3.009659
                               99%            3.009659
                               95%            2.121802
                               90%            1.574226
                               75% Q3         0.431301
                               50% Median    -0.156784
                               25% Q1        -0.469944
                               10%           -1.189137
                               5%            -1.367489
                               1%            -2.244663
                               0% Min        -2.244663


                                Extreme Observations

                     ------Lowest-----        -----Highest-----

                        Value      Obs           Value      Obs

                     -2.24466       20         2.05213       24
                     -1.71562       30         2.12180       64
                     -1.43593       11         2.22489       33
                     -1.36749       31         2.96914        5
                     -1.31655        7         3.00966       32


                                    Missing Values

                                            -----Percent Of-----
                     Missing                             Missing
                       Value       Count     All Obs         Obs

                           .           2        2.78      100.00


               Stem Leaf                            #             Boxplot
                  3 00                              2                0
                  2
                  2 112                             3                0
                  1 569                             3                0
                  1 11                              2                |
                  0 5677889                         7                |
                  0 0113344                         7             +--+--+
                 -0 444443333333322211111100000    27             *-----*
                 -0 98666555                        8             +-----+
                 -1 443331110                       9                |
                 -1 7                               1                |
                 -2 2                               1                0
                    ----+----+----+----+----+--


                                   Normal Probability Plot
                3.25+                                                 *
                    |                                             *    +
                    |                                        ** * +++++
                    |                                     ***+++++
                    |                                   +**++
                    |                              +++***
                    |                         ++++****
                    |                  ************
                    |               ****++
                    |       * *******
                    |     *+++++
               -2.25+ *++++
                     +----+----+----+----+----+----+----+----+----+----+
                         -2        -1         0        +1        +2


  Gilbert Pule: Experiment 2 data for statistical analysisNo Outliers 5cm only.SAS 73
                             Project number = 000971-Y5   08:36 Friday, March 9, 2018

                              The UNIVARIATE Procedure
                               Variable:  rOrg_C5Depth

                                       Moments

           N                          72    Sum Weights                 72
           Mean               0.00747661    Sum Observations    0.53831595
           Std Deviation      1.03728891    Variance            1.07596829
           Skewness           1.01935403    Kurtosis            2.25596482
           Uncorrected SS      76.397773    Corrected SS        76.3937482
           Coeff Variation    13873.7857    Std Error Mean      0.12224567


                             Basic Statistical Measures

                   Location                    Variability

               Mean      0.00748     Std Deviation            1.03729
               Median   -0.16573     Variance                 1.07597
               Mode     -0.88553     Range                    5.69312
                                     Interquartile Range      1.23948

       Note: The mode displayed is the smallest of 4 modes with a count of 2.


                             Tests for Location: Mu0=0

                  Test           -Statistic-    -----p Value------

                  Student's t    t  0.061161    Pr > |t|    0.9514
                  Sign           M        -4    Pr >= |M|   0.4096
                  Signed Rank    S      -117    Pr >= |S|   0.5152


                                Tests for Normality

             Test                  --Statistic---    -----p Value------

             Shapiro-Wilk          W     0.938903    Pr < W      0.0017
             Kolmogorov-Smirnov    D     0.114838    Pr > D      0.0194
             Cramer-von Mises      W-Sq  0.166996    Pr > W-Sq   0.0149
             Anderson-Darling      A-Sq  1.050266    Pr > A-Sq   0.0089


                               Quantiles (Definition 5)

                               Level          Quantile

                               100% Max       3.580610
                               99%            3.580610
                               95%            1.945960
                               90%            1.200545
                               75% Q3         0.574468
                               50% Median    -0.165726
                               25% Q1        -0.665015
                               10%           -0.959446
                               5%            -1.572057
                               1%            -2.112508
                               0% Min        -2.112508


                                Extreme Observations

                     ------Lowest-----        -----Highest-----

                        Value      Obs           Value      Obs

                     -2.11251       23         1.33303       33
                     -1.97229       31         1.94596       21
                     -1.60495       11         2.05166        4
                     -1.57206       30         3.40634       28
                     -1.18985       41         3.58061       64


                  Stem Leaf                     #             Boxplot
                     3 6                        1                0
                     3 4                        1                0
                     2
                     2 1                        1                |
                     1 9                        1                |
                     1 1222233                  7                |
                     0 66778899                 8             +-----+
                     0 1112222222333           13             |  +  |
                    -0 44333222222110          14             *-----*
                    -0 999988877776666655      18             +-----+
                    -1 2110                     4                |
                    -1 66                       2                |
                    -2 10                       2                |
                       ----+----+----+----+


                                   Normal Probability Plot
                3.75+                                                 *
                    |                                             *
                    |                                                  +
                    |                                           * +++++
                    |                                        +*+++
                    |                                   ******
                0.75+                              ++****
                    |                         ++*****
                    |                     ******
                    |            **********
                    |          ***+++
                    |     *+*+*+
               -2.25+ *++++
                     +----+----+----+----+----+----+----+----+----+----+
                         -2        -1         0        +1        +2


  Gilbert Pule: Experiment 2 data for statistical analysisNo Outliers 5cm only.SAS 74
                             Project number = 000971-Y5   08:36 Friday, March 9, 2018

                              The UNIVARIATE Procedure
                              Variable:  rTotalN5Depth

                                       Moments

           N                          71    Sum Weights                 71
           Mean               0.00184919    Sum Observations    0.13129228
           Std Deviation      1.02498583    Variance            1.05059595
           Skewness           0.24165023    Kurtosis            0.46930746
           Uncorrected SS     73.5419596    Corrected SS        73.5417168
           Coeff Variation    55428.9965    Std Error Mean      0.12164344


                             Basic Statistical Measures

                   Location                    Variability

               Mean      0.00185     Std Deviation            1.02499
               Median   -0.08865     Variance                 1.05060
               Mode     -0.61248     Range                    5.26895
                                     Interquartile Range      1.08611

       Note: The mode displayed is the smallest of 2 modes with a count of 3.


                             Tests for Location: Mu0=0

                  Test           -Statistic-    -----p Value------

                  Student's t    t  0.015202    Pr > |t|    0.9879
                  Sign           M      -2.5    Pr >= |M|   0.6353
                  Signed Rank    S       -57    Pr >= |S|   0.7465


                                Tests for Normality

             Test                  --Statistic---    -----p Value------

             Shapiro-Wilk          W     0.983434    Pr < W      0.4731
             Kolmogorov-Smirnov    D     0.083549    Pr > D     >0.1500
             Cramer-von Mises      W-Sq   0.08515    Pr > W-Sq   0.1809
             Anderson-Darling      A-Sq  0.492568    Pr > A-Sq   0.2192


                              Quantiles (Definition 5)

                              Level           Quantile

                              100% Max       2.7264362
                              99%            2.7264362
                              95%            1.9141863
                              90%            1.3793471
                              75% Q3         0.4736246
                              50% Median    -0.0886548
                              25% Q1        -0.6124829
                              10%           -1.0935686
                              5%            -1.8501122
                              1%            -2.5425126
                              0% Min        -2.5425126


                                Extreme Observations

                     ------Lowest-----        -----Highest-----

                        Value      Obs           Value      Obs

                     -2.54251       11         1.44122       43
                     -2.09768       23         1.91419       28
                     -1.97861       30         2.27325        4
                     -1.85011       31         2.31762       24
                     -1.44122       45         2.72644       21


                                    Missing Values

                                            -----Percent Of-----
                     Missing                             Missing
                       Value       Count     All Obs         Obs

                           .           1        1.39      100.00


                  Stem Leaf                     #             Boxplot
                     2 7                        1                0
                     2 33                       2                0
                     1 9                        1                |
                     1 0012334444              10                |
                     0 5667                     4             +-----+
                     0 111111223333444         15             |  +  |
                    -0 4444433222211110        16             *-----*
                    -0 999977666655            12             +-----+
                    -1 432100                   6                |
                    -1 9                        1                |
                    -2 10                       2                |
                    -2 5                        1                0
                       ----+----+----+----+


                                   Normal Probability Plot
                2.75+                                                 *+
                    |                                           * *++++
                    |                                        +*+++
                    |                                  *******
                    |                              ++***
                    |                         +*******
                    |                    *******
                    |               *****+
                    |          *****+
                    |      +*+*+
                    | ++++*
               -2.75++*
                     +----+----+----+----+----+----+----+----+----+----+
                         -2        -1         0        +1        +2


  Gilbert Pule: Experiment 2 data for statistical analysisNo Outliers 5cm only.SAS 75
                             Project number = 000971-Y5   08:36 Friday, March 9, 2018

                              The UNIVARIATE Procedure
                             Variable:  rSandPers5Depth

                                       Moments

           N                          72    Sum Weights                 72
           Mean               0.00693139    Sum Observations    0.49905986
           Std Deviation      1.01947475    Variance            1.03932876
           Skewness           0.91432432    Kurtosis            -0.3538945
           Uncorrected SS      73.795801    Corrected SS        73.7923419
           Coeff Variation    14708.0918    Std Error Mean      0.12014625


                             Basic Statistical Measures

                   Location                    Variability

               Mean     0.006931     Std Deviation            1.01947
               Median   0.000000     Variance                 1.03933
               Mode     0.000000     Range                    2.90825
                                     Interquartile Range      1.31279


                             Tests for Location: Mu0=0

                  Test           -Statistic-    -----p Value------

                  Student's t    t  0.057691    Pr > |t|    0.9542
                  Sign           M        -6    Pr >= |M|   0.1114
                  Signed Rank    S       -39    Pr >= |S|   0.6913


                                Tests for Normality

             Test                  --Statistic---    -----p Value------

             Shapiro-Wilk          W     0.795319    Pr < W     <0.0001
             Kolmogorov-Smirnov    D     0.252712    Pr > D     <0.0100
             Cramer-von Mises      W-Sq  0.811803    Pr > W-Sq  <0.0050
             Anderson-Darling      A-Sq  5.383562    Pr > A-Sq  <0.0050


                               Quantiles (Definition 5)

                               Level          Quantile

                               100% Max       1.952694
                               99%            1.952694
                               95%            1.952694
                               90%            1.952694
                               75% Q3         0.357236
                               50% Median     0.000000
                               25% Q1        -0.955553
                               10%           -0.955553
                               5%            -0.955553
                               1%            -0.955553
                               0% Min        -0.955553


                                 Extreme Observations

                     ------Lowest------        -----Highest-----

                         Value      Obs           Value      Obs

                     -0.955553       36         1.95269        4
                     -0.955553       35         1.95269       13
                     -0.955553       34         1.95269       14
                     -0.955553       33         1.95269       15
                     -0.955553       32         1.95269       16


                Stem Leaf                         #             Boxplot
                  18 555555555555                12                |
                  16                                               |
                  14                                               |
                  12                                               |
                  10                                               |
                   8                                               |
                   6 111111                       6                |
                   4                                               |
                   2                                            +-----+
                   0 000000000000000000000000    24             *--+--*
                  -0                                            |     |
                  -2                                            |     |
                  -4                                            |     |
                  -6 111111                       6             |     |
                  -8 666666666666666666666666    24             +-----+
                     ----+----+----+----+----
                 Multiply Stem.Leaf by 10**-1


                                   Normal Probability Plot
                 1.9+                                   ******* *+*   *
                    |                                         ++
                    |                                       ++
                    |                                     ++
                    |                                   ++
                    |                                 ++
                    |                               +***
                 0.5+                             ++
                    |                           ++
                    |                       **********
                    |                       ++
                    |                      +
                    |                    ++
                    |                  ++ ***
                -0.9+ *   * * *************
                     +----+----+----+----+----+----+----+----+----+----+
                         -2        -1         0        +1        +2


  Gilbert Pule: Experiment 2 data for statistical analysisNo Outliers 5cm only.SAS 76
                             Project number = 000971-Y5   08:36 Friday, March 9, 2018

                              The UNIVARIATE Procedure
                             Variable:  rSiltPers5Depth

                                       Moments

           N                          72    Sum Weights                 72
           Mean               0.00796925    Sum Observations    0.57378619
           Std Deviation      1.02309946    Variance            1.04673251
           Skewness           1.04534348    Kurtosis            0.12186469
           Uncorrected SS     74.3225806    Corrected SS         74.318008
           Coeff Variation    12838.0854    Std Error Mean      0.12057343


                             Basic Statistical Measures

                   Location                    Variability

               Mean      0.00797     Std Deviation            1.02310
               Median   -0.00000     Variance                 1.04673
               Mode     -1.00000     Range                    3.04782
                                     Interquartile Range      1.00000


                             Tests for Location: Mu0=0

                  Test           -Statistic-    -----p Value------

                  Student's t    t  0.066095    Pr > |t|    0.9475
                  Sign           M       -18    Pr >= |M|   <.0001
                  Signed Rank    S      -261    Pr >= |S|   0.0527


                                Tests for Normality

             Test                  --Statistic---    -----p Value------

             Shapiro-Wilk          W     0.742196    Pr < W     <0.0001
             Kolmogorov-Smirnov    D     0.336441    Pr > D     <0.0100
             Cramer-von Mises      W-Sq  1.313013    Pr > W-Sq  <0.0050
             Anderson-Darling      A-Sq   7.66372    Pr > A-Sq  <0.0050


                              Quantiles (Definition 5)

                               Level         Quantile

                               100% Max       2.04782
                               99%            2.04782
                               95%            2.04782
                               90%            2.04782
                               75% Q3         0.00000
                               50% Median    -0.00000
                               25% Q1        -1.00000
                               10%           -1.00000
                               5%            -1.00000
                               1%            -1.00000
                               0% Min        -1.00000


                                 Extreme Observations

                       ----Lowest----        -----Highest-----

                       Value      Obs           Value      Obs

                          -1       20         2.04782       24
                          -1       19         2.04782       33
                          -1       18         2.04782       34
                          -1       17         2.04782       35
                          -1       16         2.04782       36


          Stem Leaf                                     #             Boxplot
            20 555555555555                            12                0
            18
            16
            14
            12
            10
             8
             6
             4
             2
             0 000000000000000000000000000000000000    36             +--+--+
            -0                                                        |     |
            -2                                                        |     |
            -4                                                        |     |
            -6                                                        |     |
            -8                                                        |     |
           -10 000000000000000000000000                24             +-----+
               ----+----+----+----+----+----+----+-
           Multiply Stem.Leaf by 10**-1


                                   Normal Probability Plot
                 2.1+                                   ******* * *+  *
                    |                                           ++
                    |                                         ++
                    |                                       ++
                    |                                     ++
                    |                                   ++
                    |                                 ++
                    |                               ++
                 0.5+                             ++
                    |                           ++
                    |                     **************
                    |                       ++
                    |                      +
                    |                    ++
                    |                  ++
                    | *   * * *************
                -1.1+              ++
                     +----+----+----+----+----+----+----+----+----+----+
                         -2        -1         0        +1        +2


  Gilbert Pule: Experiment 2 data for statistical analysisNo Outliers 5cm only.SAS 77
                             Project number = 000971-Y5   08:36 Friday, March 9, 2018

                              The UNIVARIATE Procedure
                                 Variable:  rP5Depth

                                       Moments

           N                          72    Sum Weights                 72
           Mean               0.00567462    Sum Observations    0.40857287
           Std Deviation      1.02861816    Variance            1.05805532
           Skewness           0.75988088    Kurtosis            0.95141794
           Uncorrected SS     75.1242463    Corrected SS        75.1219278
           Coeff Variation    18126.6339    Std Error Mean      0.12122381


                             Basic Statistical Measures

                   Location                    Variability

               Mean      0.00567     Std Deviation            1.02862
               Median   -0.08422     Variance                 1.05806
               Mode       .          Range                    4.98088
                                     Interquartile Range      1.09891


                             Tests for Location: Mu0=0

                  Test           -Statistic-    -----p Value------

                  Student's t    t  0.046811    Pr > |t|    0.9628
                  Sign           M        -3    Pr >= |M|   0.5560
                  Signed Rank    S      -125    Pr >= |S|   0.4869


                                Tests for Normality

             Test                  --Statistic---    -----p Value------

             Shapiro-Wilk          W      0.95167    Pr < W      0.0077
             Kolmogorov-Smirnov    D     0.111732    Pr > D      0.0244
             Cramer-von Mises      W-Sq  0.184259    Pr > W-Sq   0.0084
             Anderson-Darling      A-Sq  1.122007    Pr > A-Sq   0.0060


                              Quantiles (Definition 5)

                              Level           Quantile

                              100% Max       3.0047365
                              99%            3.0047365
                              95%            1.8580337
                              90%            1.6251045
                              75% Q3         0.4052216
                              50% Median    -0.0842237
                              25% Q1        -0.6936868
                              10%           -0.9524806
                              5%            -1.7259616
                              1%            -1.9761443
                              0% Min        -1.9761443


                                Extreme Observations

                     ------Lowest-----        -----Highest-----

                        Value      Obs           Value      Obs

                     -1.97614       29         1.85658       55
                     -1.96735       31         1.85803       27
                     -1.79775       24         2.33032       16
                     -1.72596       11         2.90095       43
                     -1.42763        1         3.00474        5


                  Stem Leaf                     #             Boxplot
                    30 0                        1                0
                    28 0                        1                0
                    26
                    24
                    22 3                        1                0
                    20
                    18 66                       2                |
                    16 376                      3                |
                    14                                           |
                    12 4                        1                |
                    10 52                       2                |
                     8 1                        1                |
                     6 97                       2                |
                     4 01224                    5             +-----+
                     2 122446                   6             |     |
                     0 55602269                 8             |  +  |
                    -0 51652                    5             *-----*
                    -2 65220742                 8             |     |
                    -4 99393                    5             |     |
                    -6 96510954                 8             +-----+
                    -8 508720                   6                |
                   -10 72                       2                |
                   -12                                           |
                   -14 3                        1                |
                   -16 3                        1                |
                   -18 870                      3                |
                       ----+----+----+----+
                   Multiply Stem.Leaf by 10**-1


                                   Normal Probability Plot
                 3.1+                                                 *
                    |                                             *
                    |
                    |                                                 ++
                    |                                           *   ++
                 2.1+                                             ++
                    |                                        ** ++
                    |                                      ** ++
                    |                                       ++
                    |                                     *+
                 1.1+                                   **
                    |                                 ++*
                    |                               ++ *
                    |                             ++ **
                    |                           ++***
                 0.1+                         +****
                    |                        ***
                    |                      ***
                    |                    **
                    |                ****
                -0.9+            *****+
                    |           ** ++
                    |            ++
                    |          *+
                    |       *+*
                -1.9+ *   *++
                     +----+----+----+----+----+----+----+----+----+----+
                         -2        -1         0        +1        +2


  Gilbert Pule: Experiment 2 data for statistical analysisNo Outliers 5cm only.SAS 78
                             Project number = 000971-Y5   08:36 Friday, March 9, 2018

                              The UNIVARIATE Procedure
                               Variable:  rpHH2O5Depth

                                       Moments

           N                          72    Sum Weights                 72
           Mean               0.00422864    Sum Observations    0.30446201
           Std Deviation      1.02008834    Variance            1.04058023
           Skewness           0.56332531    Kurtosis            -0.2372563
           Uncorrected SS     73.8824836    Corrected SS        73.8811961
           Coeff Variation    24123.3252    Std Error Mean      0.12021856


                             Basic Statistical Measures

                   Location                    Variability

               Mean      0.00423     Std Deviation            1.02009
               Median   -0.21214     Variance                 1.04058
               Mode     -0.94763     Range                    4.54206
                                     Interquartile Range      1.28452

       Note: The mode displayed is the smallest of 8 modes with a count of 2.


                             Tests for Location: Mu0=0

                  Test           -Statistic-    -----p Value------

                  Student's t    t  0.035175    Pr > |t|    0.9720
                  Sign           M        -4    Pr >= |M|   0.4096
                  Signed Rank    S     -91.5    Pr >= |S|   0.6111


                                Tests for Normality

             Test                  --Statistic---    -----p Value------

             Shapiro-Wilk          W     0.963057    Pr < W      0.0329
             Kolmogorov-Smirnov    D     0.113982    Pr > D      0.0208
             Cramer-von Mises      W-Sq  0.176578    Pr > W-Sq   0.0101
             Anderson-Darling      A-Sq  0.977708    Pr > A-Sq   0.0143


                               Quantiles (Definition 5)

                               Level          Quantile

                               100% Max       2.522486
                               99%            2.522486
                               95%            2.015150
                               90%            1.444463
                               75% Q3         0.632075
                               50% Median    -0.212136
                               25% Q1        -0.652449
                               10%           -1.229886
                               5%            -1.431762
                               1%            -2.019575
                               0% Min        -2.019575


                                Extreme Observations

                     ------Lowest-----        -----Highest-----

                        Value      Obs           Value      Obs

                     -2.01957       11         1.92262        5
                     -1.56352       58         2.01515       50
                     -1.48263       30         2.18902       24
                     -1.43176       35         2.31562       28
                     -1.35998       59         2.52249       64


                  Stem Leaf                     #             Boxplot
                    24 2                        1                |
                    22 2                        1                |
                    20 29                       2                |
                    18 2                        1                |
                    16 6                        1                |
                    14 41                       2                |
                    12 71                       2                |
                    10 16257                    5                |
                     8 9                        1                |
                     6 066                      3             +-----+
                     4 1                        1             |     |
                     2 1226777                  7             |     |
                     0 68389                    5             |  +  |
                    -0 762                      3             |     |
                    -2 6630                     4             *-----*
                    -4 5554106410              10             |     |
                    -6 009555500                9             +-----+
                    -8 5500                     4                |
                   -10 15                       2                |
                   -12 6103                     4                |
                   -14 683                      3                |
                   -16                                           |
                   -18                                           |
                   -20 2                        1                |
                       ----+----+----+----+
                   Multiply Stem.Leaf by 10**-1


                                   Normal Probability Plot
                 2.5+                                                 *+
                    |                                             * ++
                    |                                         * * ++
                    |                                        *  ++
                    |                                       * ++
                    |                                      *++
                    |                                    **+
                    |                                 ***+
                    |                                 *+
                    |                               +*
                    |                             ++**
                    |                           +****
                    |                         ++**
                    |                       ++**
                    |                      + **
                    |                    +****
                    |                *****
                    |               **+
                    |             **+
                    |          ***+
                    |     * * *++
                    |        ++
                    |      ++
                -2.1+ *  ++
                     +----+----+----+----+----+----+----+----+----+----+
                         -2        -1         0        +1        +2


  Gilbert Pule: Experiment 2 data for statistical analysisNo Outliers 5cm only.SAS 79
                             Project number = 000971-Y5   08:36 Friday, March 9, 2018

                                  The GLM Procedure

                              Class Level Information

            Class                   Levels    Values

            Farm                         2    Kaalfontein Schietfontein

            GrassLandCommunity           2    GM11 Gh15

            Rainfall                     2    593 654

            SlopePosition                3    Bottom Mid Top


                            Data for Analysis of Altitude

                       Number of Observations Read          72
                       Number of Observations Used          69


                       Data for Analysis of Longitude Latitude

                       Number of Observations Read          72
                       Number of Observations Used          70


                           Data for Analysis of SplumDens
                        SplumMeanConCov IndivIntercep K5Depth
                             Org_C5Depth SandPers5Depth
                             SiltPers5Depth pHH2O5Depth

                       Number of Observations Read          72
                       Number of Observations Used          72


                         Data for Analysis of BulkDens5Depth

                       Number of Observations Read          72
                       Number of Observations Used          69


                            Data for Analysis of Na5Dpth

                       Number of Observations Read          72
                       Number of Observations Used          68


                            Data for Analysis of Ca5Depth

                       Number of Observations Read          72
                       Number of Observations Used          71


                            Data for Analysis of Mg5Depth

                       Number of Observations Read          72
                       Number of Observations Used          70


                          Data for Analysis of TotalN5Depth

                       Number of Observations Read          72
                       Number of Observations Used          71


NOTE: Variables in each group are consistent with respect to the presence or absence
      of missing values.

  Gilbert Pule: Experiment 2 data for statistical analysisNo Outliers 5cm only.SAS 80
                             Project number = 000971-Y5   08:36 Friday, March 9, 2018

                                  The GLM Procedure

                           Dependent Variable: Altitude

                                         Sum of
 Source                      DF         Squares     Mean Square    F Value    Pr > F

 Model                        5     424758.6691      84951.7338    3914.41    <.0001

 Error                       63       1367.2439         21.7023

 Corrected Total             68     426125.9130


                R-Square     Coeff Var      Root MSE    Altitude Mean

                0.996791      0.302667      4.658571         1539.174


 Source                      DF       Type I SS     Mean Square    F Value    Pr > F

 GrassLandCommunity           1     424582.6302     424582.6302    19564.0    <.0001
 SlopePosition                2        174.5427         87.2714       4.02    0.0227
 GrassLand*SlopePosit         2          1.4962          0.7481       0.03    0.9661

  Gilbert Pule: Experiment 2 data for statistical analysisNo Outliers 5cm only.SAS 81
                             Project number = 000971-Y5   08:36 Friday, March 9, 2018

                                  The GLM Procedure

             Level of                      -----------Altitude----------
             GrassLandCommunity      N             Mean          Std Dev

             GM11                   33       1457.24242       4.90554726
             Gh15                   36       1614.27778       4.70021952


               Level of                 -----------Altitude----------
               SlopePosition      N             Mean          Std Dev

               Bottom            24       1533.87500       80.4445867
               Mid               23       1539.21739       80.0982105
               Top               22       1544.90909       80.1016670


    Level of               Level of                 -----------Altitude----------
    GrassLandCommunity     SlopePosition      N             Mean          Std Dev

    GM11                   Bottom            12       1455.25000       4.91981153
    GM11                   Mid               11       1457.54545       4.92673597
    GM11                   Top               10       1459.30000       4.34741302
    Gh15                   Bottom            12       1612.50000       4.35889894
    Gh15                   Mid               12       1614.08333       4.96273996
    Gh15                   Top               12       1616.25000       4.35107092

  Gilbert Pule: Experiment 2 data for statistical analysisNo Outliers 5cm only.SAS 82
                             Project number = 000971-Y5   08:36 Friday, March 9, 2018

                                  The GLM Procedure

                             t Tests (LSD) for Altitude

       NOTE: This test controls the Type I comparisonwise error rate, not the
                             experimentwise error rate.


                        Alpha                            0.05
                        Error Degrees of Freedom           63
                        Error Mean Square            21.70228
                        Critical Value of t           1.99834
                        Least Significant Difference   2.2436
                        Harmonic Mean of Cell Sizes  34.43478

                           NOTE: Cell sizes are not equal.


             Means with the same letter are not significantly different.


                                                   Grass
                                                   Land
                t Grouping          Mean      N    Community

                         A      1614.278     36    Gh15

                         B      1457.242     33    GM11

  Gilbert Pule: Experiment 2 data for statistical analysisNo Outliers 5cm only.SAS 83
                             Project number = 000971-Y5   08:36 Friday, March 9, 2018

                                  The GLM Procedure

                             t Tests (LSD) for Altitude

       NOTE: This test controls the Type I comparisonwise error rate, not the
                             experimentwise error rate.


                        Alpha                            0.05
                        Error Degrees of Freedom           63
                        Error Mean Square            21.70228
                        Critical Value of t           1.99834
                        Least Significant Difference   2.7469
                        Harmonic Mean of Cell Sizes    22.971

                           NOTE: Cell sizes are not equal.


             Means with the same letter are not significantly different.


                                                   Slope
                t Grouping          Mean      N    Position

                         A      1544.909     22    Top

                         B      1539.217     23    Mid

                         C      1533.875     24    Bottom

  Gilbert Pule: Experiment 2 data for statistical analysisNo Outliers 5cm only.SAS 84
                             Project number = 000971-Y5   08:36 Friday, March 9, 2018

                                  The GLM Procedure

                          Dependent Variable: Longitude

                                         Sum of
 Source                      DF         Squares     Mean Square    F Value    Pr > F

 Model                        5      1.71641004      0.34328201    59595.2    <.0001

 Error                       64      0.00036865      0.00000576

 Corrected Total             69      1.71677869


               R-Square     Coeff Var      Root MSE    Longitude Mean

               0.999785      0.009256      0.002400          25.93067


 Source                      DF       Type I SS     Mean Square    F Value    Pr > F

 GrassLandCommunity           1      1.71637520      1.71637520     297970    <.0001
 SlopePosition                2      0.00000822      0.00000411       0.71    0.4937
 GrassLand*SlopePosit         2      0.00002661      0.00001331       2.31    0.1075

  Gilbert Pule: Experiment 2 data for statistical analysisNo Outliers 5cm only.SAS 85
                             Project number = 000971-Y5   08:36 Friday, March 9, 2018

                                  The GLM Procedure

                           Dependent Variable: Latitude

                                         Sum of
 Source                      DF         Squares     Mean Square    F Value    Pr > F

 Model                        5     25.79075174      5.15815035    3659825    <.0001

 Error                       64      0.00009020      0.00000141

 Corrected Total             69     25.79084195


                R-Square     Coeff Var      Root MSE    Latitude Mean

                0.999997      0.004231      0.001187         28.05684


 Source                      DF       Type I SS     Mean Square    F Value    Pr > F

 GrassLandCommunity           1     25.79074933     25.79074933     1.83E7    <.0001
 SlopePosition                2      0.00000130      0.00000065       0.46    0.6317
 GrassLand*SlopePosit         2      0.00000111      0.00000056       0.39    0.6760

  Gilbert Pule: Experiment 2 data for statistical analysisNo Outliers 5cm only.SAS 86
                             Project number = 000971-Y5   08:36 Friday, March 9, 2018

                                  The GLM Procedure

 Level of                  ---------Longitude---------   ----------Latitude---------
 GrassLandCommunity    N           Mean        Std Dev           Mean        Std Dev

 GM11                 34     25.7695476     0.00053149     28.6814312     0.00134429
 Gh15                 36     26.0828506     0.00335588     27.4669514     0.00097073


 Level of               ----------Longitude---------    ----------Latitude----------
 SlopePosition     N            Mean         Std Dev            Mean         Std Dev

 Bottom           24      25.9265175      0.16092259      28.0740496      0.62048501
 Mid              24      25.9257329      0.15949875      28.0741588      0.62021343
 Top              22      25.9406014      0.15929659      28.0191777      0.61887007


    Level of               Level of                 ----------Longitude----------
    GrassLandCommunity     SlopePosition      N             Mean          Std Dev

    GM11                   Bottom            12       25.7690208       0.00036340
    GM11                   Mid               12       25.7695942       0.00027665
    GM11                   Top               10       25.7701240       0.00019699
    Gh15                   Bottom            12       26.0840142       0.00507767
    Gh15                   Mid               12       26.0818717       0.00105859
    Gh15                   Top               12       26.0826658       0.00252396

    Level of               Level of                 -----------Latitude----------
    GrassLandCommunity     SlopePosition      N             Mean          Std Dev

    GM11                   Bottom            12       28.6814692       0.00141627
    GM11                   Mid               12       28.6813125       0.00138389
    GM11                   Top               10       28.6815280       0.00134264
    Gh15                   Bottom            12       27.4666300       0.00098021
    Gh15                   Mid               12       27.4670050       0.00099145
    Gh15                   Top               12       27.4672192       0.00092761

  Gilbert Pule: Experiment 2 data for statistical analysisNo Outliers 5cm only.SAS 87
                             Project number = 000971-Y5   08:36 Friday, March 9, 2018

                                  The GLM Procedure

                             t Tests (LSD) for Longitude

       NOTE: This test controls the Type I comparisonwise error rate, not the
                             experimentwise error rate.


                        Alpha                            0.05
                        Error Degrees of Freedom           64
                        Error Mean Square             5.76E-6
                        Critical Value of t           1.99773
                        Least Significant Difference   0.0011
                        Harmonic Mean of Cell Sizes  34.97143

                           NOTE: Cell sizes are not equal.


             Means with the same letter are not significantly different.


                                                   Grass
                                                   Land
                t Grouping          Mean      N    Community

                         A     26.082851     36    Gh15

                         B     25.769548     34    GM11

  Gilbert Pule: Experiment 2 data for statistical analysisNo Outliers 5cm only.SAS 88
                             Project number = 000971-Y5   08:36 Friday, March 9, 2018

                                  The GLM Procedure

                             t Tests (LSD) for Latitude

       NOTE: This test controls the Type I comparisonwise error rate, not the
                             experimentwise error rate.


                        Alpha                            0.05
                        Error Degrees of Freedom           64
                        Error Mean Square            1.409E-6
                        Critical Value of t           1.99773
                        Least Significant Difference   0.0006
                        Harmonic Mean of Cell Sizes  34.97143

                           NOTE: Cell sizes are not equal.


             Means with the same letter are not significantly different.


                                                   Grass
                                                   Land
                t Grouping          Mean      N    Community

                         A     28.681431     34    GM11

                         B     27.466951     36    Gh15

  Gilbert Pule: Experiment 2 data for statistical analysisNo Outliers 5cm only.SAS 89
                             Project number = 000971-Y5   08:36 Friday, March 9, 2018

                                  The GLM Procedure

                             t Tests (LSD) for Longitude

       NOTE: This test controls the Type I comparisonwise error rate, not the
                             experimentwise error rate.


                        Alpha                            0.05
                        Error Degrees of Freedom           64
                        Error Mean Square             5.76E-6
                        Critical Value of t           1.99773
                        Least Significant Difference   0.0014
                        Harmonic Mean of Cell Sizes  23.29412

                           NOTE: Cell sizes are not equal.


             Means with the same letter are not significantly different.


                                                   Slope
                t Grouping          Mean      N    Position

                         A     25.940601     22    Top

                         B     25.926518     24    Bottom
                         B
                         B     25.925733     24    Mid

  Gilbert Pule: Experiment 2 data for statistical analysisNo Outliers 5cm only.SAS 90
                             Project number = 000971-Y5   08:36 Friday, March 9, 2018

                                  The GLM Procedure

                             t Tests (LSD) for Latitude

       NOTE: This test controls the Type I comparisonwise error rate, not the
                             experimentwise error rate.


                        Alpha                            0.05
                        Error Degrees of Freedom           64
                        Error Mean Square            1.409E-6
                        Critical Value of t           1.99773
                        Least Significant Difference   0.0007
                        Harmonic Mean of Cell Sizes  23.29412

                           NOTE: Cell sizes are not equal.


             Means with the same letter are not significantly different.


                                                   Slope
                t Grouping          Mean      N    Position

                         A     28.074159     24    Mid
                         A
                         A     28.074050     24    Bottom

                         B     28.019178     22    Top

  Gilbert Pule: Experiment 2 data for statistical analysisNo Outliers 5cm only.SAS 91
                             Project number = 000971-Y5   08:36 Friday, March 9, 2018

                                  The GLM Procedure

                          Dependent Variable: SplumDens

                                         Sum of
 Source                      DF         Squares     Mean Square    F Value    Pr > F

 Model                        5     12702.56944      2540.51389       7.64    <.0001

 Error                       66     21945.08333       332.50126

 Corrected Total             71     34647.65278


               R-Square     Coeff Var      Root MSE    SplumDens Mean

               0.366621      57.55776      18.23462          31.68056


 Source                      DF       Type I SS     Mean Square    F Value    Pr > F

 GrassLandCommunity           1     5356.125000     5356.125000      16.11    0.0002
 SlopePosition                2     2638.361111     1319.180556       3.97    0.0236
 GrassLand*SlopePosit         2     4708.083333     2354.041667       7.08    0.0016

  Gilbert Pule: Experiment 2 data for statistical analysisNo Outliers 5cm only.SAS 92
                             Project number = 000971-Y5   08:36 Friday, March 9, 2018

                                  The GLM Procedure

                       Dependent Variable: SplumMeanConCov

                                         Sum of
 Source                      DF         Squares     Mean Square    F Value    Pr > F

 Model                        5      4.34661129      0.86932226       2.65    0.0301

 Error                       66     21.61064058      0.32743395

 Corrected Total             71     25.95725188


            R-Square     Coeff Var      Root MSE    SplumMeanConCov Mean

            0.167453      46.43845      0.572218                1.232208


 Source                      DF       Type I SS     Mean Square    F Value    Pr > F

 GrassLandCommunity           1      1.90808112      1.90808112       5.83    0.0186
 SlopePosition                2      2.40637758      1.20318879       3.67    0.0307
 GrassLand*SlopePosit         2      0.03215258      0.01607629       0.05    0.9521

  Gilbert Pule: Experiment 2 data for statistical analysisNo Outliers 5cm only.SAS 93
                             Project number = 000971-Y5   08:36 Friday, March 9, 2018

                                  The GLM Procedure

                        Dependent Variable: IndivIntercep

                                         Sum of
 Source                      DF         Squares     Mean Square    F Value    Pr > F

 Model                        5      388.777778       77.755556       2.13    0.0728

 Error                       66     2410.500000       36.522727

 Corrected Total             71     2799.277778


             R-Square     Coeff Var      Root MSE    IndivIntercep Mean

             0.138885      53.45517      6.043404              11.30556


 Source                      DF       Type I SS     Mean Square    F Value    Pr > F

 GrassLandCommunity           1     180.5000000     180.5000000       4.94    0.0296
 SlopePosition                2      50.0277778      25.0138889       0.68    0.5077
 GrassLand*SlopePosit         2     158.2500000      79.1250000       2.17    0.1227

  Gilbert Pule: Experiment 2 data for statistical analysisNo Outliers 5cm only.SAS 94
                             Project number = 000971-Y5   08:36 Friday, March 9, 2018

                                  The GLM Procedure

                           Dependent Variable: K5Depth

                                         Sum of
 Source                      DF         Squares     Mean Square    F Value    Pr > F

 Model                        5      71473.0866      14294.6173      10.83    <.0001

 Error                       66      87074.6859       1319.3134

 Corrected Total             71     158547.7725


                R-Square     Coeff Var      Root MSE    K5Depth Mean

                0.450798      43.30577      36.32235        83.87417


 Source                      DF       Type I SS     Mean Square    F Value    Pr > F

 GrassLandCommunity           1     69946.72694     69946.72694      53.02    <.0001
 SlopePosition                2       101.82563        50.91282       0.04    0.9622
 GrassLand*SlopePosit         2      1424.53404       712.26702       0.54    0.5854

  Gilbert Pule: Experiment 2 data for statistical analysisNo Outliers 5cm only.SAS 95
                             Project number = 000971-Y5   08:36 Friday, March 9, 2018

                                  The GLM Procedure

                         Dependent Variable: Org_C5Depth

                                         Sum of
 Source                      DF         Squares     Mean Square    F Value    Pr > F

 Model                        5     10.56347917      2.11269583      33.74    <.0001

 Error                       66      4.13260833      0.06261528

 Corrected Total             71     14.69608750


              R-Square     Coeff Var      Root MSE    Org_C5Depth Mean

              0.718795      21.87807      0.250230            1.143750


 Source                      DF       Type I SS     Mean Square    F Value    Pr > F

 GrassLandCommunity           1     10.29823472     10.29823472     164.47    <.0001
 SlopePosition                2      0.16043333      0.08021667       1.28    0.2845
 GrassLand*SlopePosit         2      0.10481111      0.05240556       0.84    0.4376

  Gilbert Pule: Experiment 2 data for statistical analysisNo Outliers 5cm only.SAS 96
                             Project number = 000971-Y5   08:36 Friday, March 9, 2018

                                  The GLM Procedure

                        Dependent Variable: SandPers5Depth

                                         Sum of
 Source                      DF         Squares     Mean Square    F Value    Pr > F

 Model                        5     9530.000000     1906.000000     898.54    <.0001

 Error                       66      140.000000        2.121212

 Corrected Total             71     9670.000000


             R-Square     Coeff Var      Root MSE    SandPers5Depth Mean

             0.985522      1.946243      1.456438               74.83333


 Source                      DF       Type I SS     Mean Square    F Value    Pr > F

 GrassLandCommunity           1     9522.000000     9522.000000    4488.94    <.0001
 SlopePosition                2        4.000000        2.000000       0.94    0.3947
 GrassLand*SlopePosit         2        4.000000        2.000000       0.94    0.3947

  Gilbert Pule: Experiment 2 data for statistical analysisNo Outliers 5cm only.SAS 97
                             Project number = 000971-Y5   08:36 Friday, March 9, 2018

                                  The GLM Procedure

                        Dependent Variable: SiltPers5Depth

                                         Sum of
 Source                      DF         Squares     Mean Square    F Value    Pr > F

 Model                        5     680.0000000     136.0000000     280.50    <.0001

 Error                       66      32.0000000       0.4848485

 Corrected Total             71     712.0000000


             R-Square     Coeff Var      Root MSE    SiltPers5Depth Mean

             0.955056      12.28783      0.696311               5.666667


 Source                      DF       Type I SS     Mean Square    F Value    Pr > F

 GrassLandCommunity           1     648.0000000     648.0000000    1336.50    <.0001
 SlopePosition                2      16.0000000       8.0000000      16.50    <.0001
 GrassLand*SlopePosit         2      16.0000000       8.0000000      16.50    <.0001

  Gilbert Pule: Experiment 2 data for statistical analysisNo Outliers 5cm only.SAS 98
                             Project number = 000971-Y5   08:36 Friday, March 9, 2018

                                  The GLM Procedure

                         Dependent Variable: pHH2O5Depth

                                         Sum of
 Source                      DF         Squares     Mean Square    F Value    Pr > F

 Model                        5      0.28921111      0.05784222       1.26    0.2896

 Error                       66      3.01810000      0.04572879

 Corrected Total             71      3.30731111


              R-Square     Coeff Var      Root MSE    pHH2O5Depth Mean

              0.087446      4.378537      0.213843            4.883889


 Source                      DF       Type I SS     Mean Square    F Value    Pr > F

 GrassLandCommunity           1      0.01445000      0.01445000       0.32    0.5759
 SlopePosition                2      0.00923611      0.00461806       0.10    0.9041
 GrassLand*SlopePosit         2      0.26552500      0.13276250       2.90    0.0619

  Gilbert Pule: Experiment 2 data for statistical analysisNo Outliers 5cm only.SAS 99
                             Project number = 000971-Y5   08:36 Friday, March 9, 2018

                                  The GLM Procedure

 Level of                  ---------SplumDens---------   ------SplumMeanConCov------
 GrassLandCommunity    N           Mean        Std Dev           Mean        Std Dev

 GM11                 36     40.3055556     25.1553112     1.06941667     0.51965568
 Gh15                 36     23.0555556     14.2867460     1.39500000     0.64581510

 Level of                  -------IndivIntercep-------   ----------K5Depth----------
 GrassLandCommunity    N           Mean        Std Dev           Mean        Std Dev

 GM11                 36     12.8888889     7.32423678      52.705556     26.8407928
 Gh15                 36      9.7222222     4.60193196     115.042778     42.5562015

 Level of                  --------Org_C5Depth--------   -------SandPers5Depth------
 GrassLandCommunity    N           Mean        Std Dev           Mean        Std Dev

 GM11                 36     0.76555556     0.20772846     86.3333333     0.75592895
 Gh15                 36     1.52194444     0.28723131     63.3333333     1.91236577

 Level of                  -------SiltPers5Depth------   --------pHH2O5Depth--------
 GrassLandCommunity    N           Mean        Std Dev           Mean        Std Dev

 GM11                 36     2.66666667     0.95618289     4.89805556     0.20211481
 Gh15                 36     8.66666667     0.95618289     4.86972222     0.23071920


 Level of               ----------SplumDens---------    -------SplumMeanConCov------
 SlopePosition     N            Mean         Std Dev            Mean         Std Dev

 Bottom           24      39.3750000      29.6703812      0.98612500      0.57687135
 Mid              24      31.0833333      17.6756198      1.28658333      0.54763062
 Top              24      24.5833333      14.1049380      1.42391667      0.62551675

 Level of               --------IndivIntercep-------    -----------K5Depth----------
 SlopePosition     N            Mean         Std Dev            Mean         Std Dev

 Bottom           24      12.3333333      7.64236855      84.1675000      53.6082625
 Mid              24      11.2916667      5.98170520      85.1616667      42.3465312
 Top              24      10.2916667      5.03448254      82.2933333      47.1368190

 Level of               ---------Org_C5Depth--------    -------SandPers5Depth-------
 SlopePosition     N            Mean         Std Dev            Mean         Std Dev

 Bottom           24      1.16708333      0.40081959      74.6666667      11.6569318
 Mid              24      1.07791667      0.43359949      74.6666667      11.6569318
 Top              24      1.18625000      0.53227822      75.1666667      12.1857603

 Level of               -------SiltPers5Depth-------    ---------pHH2O5Depth--------
 SlopePosition     N            Mean         Std Dev            Mean         Std Dev

 Bottom           24      6.33333333      2.47889644      4.89916667      0.21449469
 Mid              24      5.33333333      3.47245893      4.88041667      0.21786472
 Top              24      5.33333333      3.47245893      4.87208333      0.22343133


    Level of               Level of                 ----------SplumDens----------
    GrassLandCommunity     SlopePosition      N             Mean          Std Dev

    GM11                   Bottom            12       59.0000000       29.8633250
    GM11                   Mid               12       36.9166667       16.9032452
    GM11                   Top               12       25.0000000       13.6581644
    Gh15                   Bottom            12       19.7500000       10.4196056
    Gh15                   Mid               12       25.2500000       17.1258709
    Gh15                   Top               12       24.1666667       15.1347483

    Level of               Level of                 -------SplumMeanConCov-------
    GrassLandCommunity     SlopePosition      N             Mean          Std Dev

    GM11                   Bottom            12       0.83141667       0.63758272
    GM11                   Mid               12       1.09483333       0.49624807
    GM11                   Top               12       1.28200000       0.30765654
    Gh15                   Bottom            12       1.14083333       0.48690970
    Gh15                   Mid               12       1.47833333       0.54825066
    Gh15                   Top               12       1.56583333       0.82432847

    Level of               Level of                 --------IndivIntercep--------
    GrassLandCommunity     SlopePosition      N             Mean          Std Dev

    GM11                   Bottom            12       15.6666667       9.01849951
    GM11                   Mid               12       13.0000000       6.56436662
    GM11                   Top               12       10.0000000       5.35978290
    Gh15                   Bottom            12        9.0000000       4.06761042
    Gh15                   Mid               12        9.5833333       5.03548018
    Gh15                   Top               12       10.5833333       4.90747729

    Level of               Level of                 -----------K5Depth-----------
    GrassLandCommunity     SlopePosition      N             Mean          Std Dev

    GM11                   Bottom            12        58.383333       36.0719786
    GM11                   Mid               12        54.117500       20.9554459
    GM11                   Top               12        45.615833       21.4424064
    Gh15                   Bottom            12       109.951667       57.0722282
    Gh15                   Mid               12       116.205833       34.7511354
    Gh15                   Top               12       118.970833       35.3681319

    Level of               Level of                 ---------Org_C5Depth---------
    GrassLandCommunity     SlopePosition      N             Mean          Std Dev

    GM11                   Bottom            12       0.83416667       0.19133304
    GM11                   Mid               12       0.70250000       0.13896533
    GM11                   Top               12       0.76000000       0.26809089
    Gh15                   Bottom            12       1.50000000       0.23977262
    Gh15                   Mid               12       1.45333333       0.25748198
    Gh15                   Top               12       1.61250000       0.35229443

    Level of               Level of                 --------SandPers5Depth-------
    GrassLandCommunity     SlopePosition      N             Mean          Std Dev

    GM11                   Bottom            12       86.0000000       0.00000000
    GM11                   Mid               12       86.0000000       0.00000000
    GM11                   Top               12       87.0000000       1.04446594
    Gh15                   Bottom            12       63.3333333       1.96946386
    Gh15                   Mid               12       63.3333333       1.96946386
    Gh15                   Top               12       63.3333333       1.96946386

    Level of               Level of                 --------SiltPers5Depth-------
    GrassLandCommunity     SlopePosition      N             Mean          Std Dev

    GM11                   Bottom            12       4.00000000       0.00000000
    GM11                   Mid               12       2.00000000       0.00000000
    GM11                   Top               12       2.00000000       0.00000000
    Gh15                   Bottom            12       8.66666667       0.98473193
    Gh15                   Mid               12       8.66666667       0.98473193
    Gh15                   Top               12       8.66666667       0.98473193

    Level of               Level of                 ---------pHH2O5Depth---------
    GrassLandCommunity     SlopePosition      N             Mean          Std Dev

    GM11                   Bottom            12       4.97416667       0.15704419
    GM11                   Mid               12       4.91666667       0.23580937
    GM11                   Top               12       4.80333333       0.18227518
    Gh15                   Bottom            12       4.82416667       0.24343968
    Gh15                   Mid               12       4.84416667       0.20192070
    Gh15                   Top               12       4.94083333       0.24666718

  Gilbert Pule: Experiment 2 data for statistical analysisNo Outliers 5cm only.SA 100
                             Project number = 000971-Y5   08:36 Friday, March 9, 2018

                                  The GLM Procedure

                             t Tests (LSD) for SplumDens

       NOTE: This test controls the Type I comparisonwise error rate, not the
                             experimentwise error rate.


                        Alpha                            0.05
                        Error Degrees of Freedom           66
                        Error Mean Square            332.5013
                        Critical Value of t           1.99656
                        Least Significant Difference   8.5811


             Means with the same letter are not significantly different.


                                                   Grass
                                                   Land
                t Grouping          Mean      N    Community

                         A        40.306     36    GM11

                         B        23.056     36    Gh15

  Gilbert Pule: Experiment 2 data for statistical analysisNo Outliers 5cm only.SA 101
                             Project number = 000971-Y5   08:36 Friday, March 9, 2018

                                  The GLM Procedure

                          t Tests (LSD) for SplumMeanConCov

       NOTE: This test controls the Type I comparisonwise error rate, not the
                             experimentwise error rate.


                        Alpha                            0.05
                        Error Degrees of Freedom           66
                        Error Mean Square            0.327434
                        Critical Value of t           1.99656
                        Least Significant Difference   0.2693


             Means with the same letter are not significantly different.


                                                   Grass
                                                   Land
                t Grouping          Mean      N    Community

                         A        1.3950     36    Gh15

                         B        1.0694     36    GM11

  Gilbert Pule: Experiment 2 data for statistical analysisNo Outliers 5cm only.SA 102
                             Project number = 000971-Y5   08:36 Friday, March 9, 2018

                                  The GLM Procedure

                           t Tests (LSD) for IndivIntercep

       NOTE: This test controls the Type I comparisonwise error rate, not the
                             experimentwise error rate.


                        Alpha                            0.05
                        Error Degrees of Freedom           66
                        Error Mean Square            36.52273
                        Critical Value of t           1.99656
                        Least Significant Difference    2.844


             Means with the same letter are not significantly different.


                                                   Grass
                                                   Land
                t Grouping          Mean      N    Community

                         A        12.889     36    GM11

                         B         9.722     36    Gh15

  Gilbert Pule: Experiment 2 data for statistical analysisNo Outliers 5cm only.SA 103
                             Project number = 000971-Y5   08:36 Friday, March 9, 2018

                                  The GLM Procedure

                              t Tests (LSD) for K5Depth

       NOTE: This test controls the Type I comparisonwise error rate, not the
                             experimentwise error rate.


                        Alpha                            0.05
                        Error Degrees of Freedom           66
                        Error Mean Square            1319.313
                        Critical Value of t           1.99656
                        Least Significant Difference   17.093


             Means with the same letter are not significantly different.


                                                   Grass
                                                   Land
                t Grouping          Mean      N    Community

                         A       115.043     36    Gh15

                         B        52.706     36    GM11

  Gilbert Pule: Experiment 2 data for statistical analysisNo Outliers 5cm only.SA 104
                             Project number = 000971-Y5   08:36 Friday, March 9, 2018

                                  The GLM Procedure

                            t Tests (LSD) for Org_C5Depth

       NOTE: This test controls the Type I comparisonwise error rate, not the
                             experimentwise error rate.


                        Alpha                            0.05
                        Error Degrees of Freedom           66
                        Error Mean Square            0.062615
                        Critical Value of t           1.99656
                        Least Significant Difference   0.1178


             Means with the same letter are not significantly different.


                                                   Grass
                                                   Land
                t Grouping          Mean      N    Community

                         A       1.52194     36    Gh15

                         B       0.76556     36    GM11

  Gilbert Pule: Experiment 2 data for statistical analysisNo Outliers 5cm only.SA 105
                             Project number = 000971-Y5   08:36 Friday, March 9, 2018

                                  The GLM Procedure

                          t Tests (LSD) for SandPers5Depth

       NOTE: This test controls the Type I comparisonwise error rate, not the
                             experimentwise error rate.


                        Alpha                            0.05
                        Error Degrees of Freedom           66
                        Error Mean Square            2.121212
                        Critical Value of t           1.99656
                        Least Significant Difference   0.6854


             Means with the same letter are not significantly different.


                                                   Grass
                                                   Land
                t Grouping          Mean      N    Community

                         A       86.3333     36    GM11

                         B       63.3333     36    Gh15

  Gilbert Pule: Experiment 2 data for statistical analysisNo Outliers 5cm only.SA 106
                             Project number = 000971-Y5   08:36 Friday, March 9, 2018

                                  The GLM Procedure

                          t Tests (LSD) for SiltPers5Depth

       NOTE: This test controls the Type I comparisonwise error rate, not the
                             experimentwise error rate.


                        Alpha                            0.05
                        Error Degrees of Freedom           66
                        Error Mean Square            0.484848
                        Critical Value of t           1.99656
                        Least Significant Difference   0.3277


             Means with the same letter are not significantly different.


                                                   Grass
                                                   Land
                t Grouping          Mean      N    Community

                         A        8.6667     36    Gh15

                         B        2.6667     36    GM11

  Gilbert Pule: Experiment 2 data for statistical analysisNo Outliers 5cm only.SA 107
                             Project number = 000971-Y5   08:36 Friday, March 9, 2018

                                  The GLM Procedure

                            t Tests (LSD) for pHH2O5Depth

       NOTE: This test controls the Type I comparisonwise error rate, not the
                             experimentwise error rate.


                        Alpha                            0.05
                        Error Degrees of Freedom           66
                        Error Mean Square            0.045729
                        Critical Value of t           1.99656
                        Least Significant Difference   0.1006


             Means with the same letter are not significantly different.


                                                   Grass
                                                   Land
                t Grouping          Mean      N    Community

                         A       4.89806     36    GM11
                         A
                         A       4.86972     36    Gh15

  Gilbert Pule: Experiment 2 data for statistical analysisNo Outliers 5cm only.SA 108
                             Project number = 000971-Y5   08:36 Friday, March 9, 2018

                                  The GLM Procedure

                             t Tests (LSD) for SplumDens

       NOTE: This test controls the Type I comparisonwise error rate, not the
                             experimentwise error rate.


                        Alpha                            0.05
                        Error Degrees of Freedom           66
                        Error Mean Square            332.5013
                        Critical Value of t           1.99656
                        Least Significant Difference    10.51


             Means with the same letter are not significantly different.


                                                      Slope
                   t Grouping          Mean      N    Position

                            A        39.375     24    Bottom
                            A
                       B    A        31.083     24    Mid
                       B
                       B             24.583     24    Top

  Gilbert Pule: Experiment 2 data for statistical analysisNo Outliers 5cm only.SA 109
                             Project number = 000971-Y5   08:36 Friday, March 9, 2018

                                  The GLM Procedure

                          t Tests (LSD) for SplumMeanConCov

       NOTE: This test controls the Type I comparisonwise error rate, not the
                             experimentwise error rate.


                        Alpha                            0.05
                        Error Degrees of Freedom           66
                        Error Mean Square            0.327434
                        Critical Value of t           1.99656
                        Least Significant Difference   0.3298


             Means with the same letter are not significantly different.


                                                      Slope
                   t Grouping          Mean      N    Position

                            A        1.4239     24    Top
                            A
                       B    A        1.2866     24    Mid
                       B
                       B             0.9861     24    Bottom

  Gilbert Pule: Experiment 2 data for statistical analysisNo Outliers 5cm only.SA 110
                             Project number = 000971-Y5   08:36 Friday, March 9, 2018

                                  The GLM Procedure

                           t Tests (LSD) for IndivIntercep

       NOTE: This test controls the Type I comparisonwise error rate, not the
                             experimentwise error rate.


                        Alpha                            0.05
                        Error Degrees of Freedom           66
                        Error Mean Square            36.52273
                        Critical Value of t           1.99656
                        Least Significant Difference   3.4832


             Means with the same letter are not significantly different.


                                                   Slope
                t Grouping          Mean      N    Position

                         A        12.333     24    Bottom
                         A
                         A        11.292     24    Mid
                         A
                         A        10.292     24    Top

  Gilbert Pule: Experiment 2 data for statistical analysisNo Outliers 5cm only.SA 111
                             Project number = 000971-Y5   08:36 Friday, March 9, 2018

                                  The GLM Procedure

                              t Tests (LSD) for K5Depth

       NOTE: This test controls the Type I comparisonwise error rate, not the
                             experimentwise error rate.


                        Alpha                            0.05
                        Error Degrees of Freedom           66
                        Error Mean Square            1319.313
                        Critical Value of t           1.99656
                        Least Significant Difference   20.935


             Means with the same letter are not significantly different.


                                                   Slope
                t Grouping          Mean      N    Position

                         A         85.16     24    Mid
                         A
                         A         84.17     24    Bottom
                         A
                         A         82.29     24    Top

  Gilbert Pule: Experiment 2 data for statistical analysisNo Outliers 5cm only.SA 112
                             Project number = 000971-Y5   08:36 Friday, March 9, 2018

                                  The GLM Procedure

                            t Tests (LSD) for Org_C5Depth

       NOTE: This test controls the Type I comparisonwise error rate, not the
                             experimentwise error rate.


                        Alpha                            0.05
                        Error Degrees of Freedom           66
                        Error Mean Square            0.062615
                        Critical Value of t           1.99656
                        Least Significant Difference   0.1442


             Means with the same letter are not significantly different.


                                                   Slope
                t Grouping          Mean      N    Position

                         A       1.18625     24    Top
                         A
                         A       1.16708     24    Bottom
                         A
                         A       1.07792     24    Mid

  Gilbert Pule: Experiment 2 data for statistical analysisNo Outliers 5cm only.SA 113
                             Project number = 000971-Y5   08:36 Friday, March 9, 2018

                                  The GLM Procedure

                          t Tests (LSD) for SandPers5Depth

       NOTE: This test controls the Type I comparisonwise error rate, not the
                             experimentwise error rate.


                        Alpha                            0.05
                        Error Degrees of Freedom           66
                        Error Mean Square            2.121212
                        Critical Value of t           1.99656
                        Least Significant Difference   0.8394


             Means with the same letter are not significantly different.


                                                   Slope
                t Grouping          Mean      N    Position

                         A       75.1667     24    Top
                         A
                         A       74.6667     24    Mid
                         A
                         A       74.6667     24    Bottom

  Gilbert Pule: Experiment 2 data for statistical analysisNo Outliers 5cm only.SA 114
                             Project number = 000971-Y5   08:36 Friday, March 9, 2018

                                  The GLM Procedure

                          t Tests (LSD) for SiltPers5Depth

       NOTE: This test controls the Type I comparisonwise error rate, not the
                             experimentwise error rate.


                        Alpha                            0.05
                        Error Degrees of Freedom           66
                        Error Mean Square            0.484848
                        Critical Value of t           1.99656
                        Least Significant Difference   0.4013


             Means with the same letter are not significantly different.


                                                   Slope
                t Grouping          Mean      N    Position

                         A        6.3333     24    Bottom

                         B        5.3333     24    Mid
                         B
                         B        5.3333     24    Top

  Gilbert Pule: Experiment 2 data for statistical analysisNo Outliers 5cm only.SA 115
                             Project number = 000971-Y5   08:36 Friday, March 9, 2018

                                  The GLM Procedure

                            t Tests (LSD) for pHH2O5Depth

       NOTE: This test controls the Type I comparisonwise error rate, not the
                             experimentwise error rate.


                        Alpha                            0.05
                        Error Degrees of Freedom           66
                        Error Mean Square            0.045729
                        Critical Value of t           1.99656
                        Least Significant Difference   0.1233


             Means with the same letter are not significantly different.


                                                   Slope
                t Grouping          Mean      N    Position

                         A       4.89917     24    Bottom
                         A
                         A       4.88042     24    Mid
                         A
                         A       4.87208     24    Top

  Gilbert Pule: Experiment 2 data for statistical analysisNo Outliers 5cm only.SA 116
                             Project number = 000971-Y5   08:36 Friday, March 9, 2018

                                  The GLM Procedure

                        Dependent Variable: BulkDens5Depth

                                         Sum of
 Source                      DF         Squares     Mean Square    F Value    Pr > F

 Model                        5      0.45275940      0.09055188      66.42    <.0001

 Error                       63      0.08589182      0.00136336

 Corrected Total             68      0.53865122


             R-Square     Coeff Var      Root MSE    BulkDens5Depth Mean

             0.840543      3.367484      0.036924               1.096478


 Source                      DF       Type I SS     Mean Square    F Value    Pr > F

 GrassLandCommunity           1      0.45106469      0.45106469     330.85    <.0001
 SlopePosition                2      0.00147626      0.00073813       0.54    0.5846
 GrassLand*SlopePosit         2      0.00021845      0.00010922       0.08    0.9231

  Gilbert Pule: Experiment 2 data for statistical analysisNo Outliers 5cm only.SA 117
                             Project number = 000971-Y5   08:36 Friday, March 9, 2018

                                  The GLM Procedure

             Level of                      --------BulkDens5Depth-------
             GrassLandCommunity      N             Mean          Std Dev

             GM11                   36       1.17388889       0.03137586
             Gh15                   33       1.01203030       0.04074730


               Level of                 --------BulkDens5Depth-------
               SlopePosition      N             Mean          Std Dev

               Bottom            23       1.09160870       0.08781216
               Mid               23       1.10269565       0.09036815
               Top               23       1.09513043       0.09241030


    Level of               Level of                 --------BulkDens5Depth-------
    GrassLandCommunity     SlopePosition      N             Mean          Std Dev

    GM11                   Bottom            12       1.16666667       0.02674232
    GM11                   Mid               12       1.18083333       0.02274696
    GM11                   Top               12       1.17416667       0.04231018
    Gh15                   Bottom            11       1.00972727       0.04518870
    Gh15                   Mid               11       1.01745455       0.04558150
    Gh15                   Top               11       1.00890909       0.03383033

  Gilbert Pule: Experiment 2 data for statistical analysisNo Outliers 5cm only.SA 118
                             Project number = 000971-Y5   08:36 Friday, March 9, 2018

                                  The GLM Procedure

                          t Tests (LSD) for BulkDens5Depth

       NOTE: This test controls the Type I comparisonwise error rate, not the
                             experimentwise error rate.


                        Alpha                            0.05
                        Error Degrees of Freedom           63
                        Error Mean Square            0.001363
                        Critical Value of t           1.99834
                        Least Significant Difference   0.0178
                        Harmonic Mean of Cell Sizes  34.43478

                           NOTE: Cell sizes are not equal.


             Means with the same letter are not significantly different.


                                                   Grass
                                                   Land
                t Grouping          Mean      N    Community

                         A      1.173889     36    GM11

                         B      1.012030     33    Gh15

  Gilbert Pule: Experiment 2 data for statistical analysisNo Outliers 5cm only.SA 119
                             Project number = 000971-Y5   08:36 Friday, March 9, 2018

                                  The GLM Procedure

                          t Tests (LSD) for BulkDens5Depth

       NOTE: This test controls the Type I comparisonwise error rate, not the
                             experimentwise error rate.


                        Alpha                            0.05
                        Error Degrees of Freedom           63
                        Error Mean Square            0.001363
                        Critical Value of t           1.99834
                        Least Significant Difference   0.0218


             Means with the same letter are not significantly different.


                                                   Slope
                t Grouping          Mean      N    Position

                         A       1.10270     23    Mid
                         A
                         A       1.09513     23    Top
                         A
                         A       1.09161     23    Bottom

  Gilbert Pule: Experiment 2 data for statistical analysisNo Outliers 5cm only.SA 120
                             Project number = 000971-Y5   08:36 Friday, March 9, 2018

                                  The GLM Procedure

                           Dependent Variable: Na5Dpth

                                         Sum of
 Source                      DF         Squares     Mean Square    F Value    Pr > F

 Model                        5     179.0531742      35.8106348      18.70    <.0001

 Error                       62     118.7512729       1.9153431

 Corrected Total             67     297.8044471


                R-Square     Coeff Var      Root MSE    Na5Dpth Mean

                0.601244      61.67053      1.383959        2.244118


 Source                      DF       Type I SS     Mean Square    F Value    Pr > F

 GrassLandCommunity           1     153.5177485     153.5177485      80.15    <.0001
 SlopePosition                2       9.1320750       4.5660375       2.38    0.1006
 GrassLand*SlopePosit         2      16.4033507       8.2016753       4.28    0.0181

  Gilbert Pule: Experiment 2 data for statistical analysisNo Outliers 5cm only.SA 121
                             Project number = 000971-Y5   08:36 Friday, March 9, 2018

                                  The GLM Procedure

             Level of                      -----------Na5Dpth-----------
             GrassLandCommunity      N             Mean          Std Dev

             GM11                   35       0.78514286       1.21025853
             Gh15                   33       3.79151515       1.71833881


               Level of                 -----------Na5Dpth-----------
               SlopePosition      N             Mean          Std Dev

               Bottom            24       2.78083333       2.73115029
               Mid               21       2.00523810       1.79183877
               Top               23       1.90217391       1.52596305


    Level of               Level of                 -----------Na5Dpth-----------
    GrassLandCommunity     SlopePosition      N             Mean          Std Dev

    GM11                   Bottom            12       0.63416667       1.04993037
    GM11                   Mid               11       0.79818182       1.39579954
    GM11                   Top               12       0.92416667       1.26795797
    Gh15                   Bottom            12       4.92750000       2.10710972
    Gh15                   Mid               10       3.33300000       1.10929457
    Gh15                   Top               11       2.96909091       0.97693863

  Gilbert Pule: Experiment 2 data for statistical analysisNo Outliers 5cm only.SA 122
                             Project number = 000971-Y5   08:36 Friday, March 9, 2018

                                  The GLM Procedure

                              t Tests (LSD) for Na5Dpth

       NOTE: This test controls the Type I comparisonwise error rate, not the
                             experimentwise error rate.


                        Alpha                            0.05
                        Error Degrees of Freedom           62
                        Error Mean Square            1.915343
                        Critical Value of t           1.99897
                        Least Significant Difference   0.6713
                        Harmonic Mean of Cell Sizes  33.97059

                           NOTE: Cell sizes are not equal.


             Means with the same letter are not significantly different.


                                                   Grass
                                                   Land
                t Grouping          Mean      N    Community

                         A        3.7915     33    Gh15

                         B        0.7851     35    GM11

  Gilbert Pule: Experiment 2 data for statistical analysisNo Outliers 5cm only.SA 123
                             Project number = 000971-Y5   08:36 Friday, March 9, 2018

                                  The GLM Procedure

                              t Tests (LSD) for Na5Dpth

       NOTE: This test controls the Type I comparisonwise error rate, not the
                             experimentwise error rate.


                        Alpha                            0.05
                        Error Degrees of Freedom           62
                        Error Mean Square            1.915343
                        Critical Value of t           1.99897
                        Least Significant Difference    0.823
                        Harmonic Mean of Cell Sizes  22.59649

                           NOTE: Cell sizes are not equal.


             Means with the same letter are not significantly different.


                                                      Slope
                   t Grouping          Mean      N    Position

                            A        2.7808     24    Bottom
                            A
                       B    A        2.0052     21    Mid
                       B
                       B             1.9022     23    Top

  Gilbert Pule: Experiment 2 data for statistical analysisNo Outliers 5cm only.SA 124
                             Project number = 000971-Y5   08:36 Friday, March 9, 2018

                                  The GLM Procedure

                           Dependent Variable: Ca5Depth

                                         Sum of
 Source                      DF         Squares     Mean Square    F Value    Pr > F

 Model                        5     296506.8404      59301.3681      21.16    <.0001

 Error                       65     182145.7799       2802.2428

 Corrected Total             70     478652.6202


                R-Square     Coeff Var      Root MSE    Ca5Depth Mean

                0.619461      73.75524      52.93621         71.77282


 Source                      DF       Type I SS     Mean Square    F Value    Pr > F

 GrassLandCommunity           1     293393.9165     293393.9165     104.70    <.0001
 SlopePosition                2       2507.4531       1253.7266       0.45    0.6412
 GrassLand*SlopePosit         2        605.4708        302.7354       0.11    0.8978

  Gilbert Pule: Experiment 2 data for statistical analysisNo Outliers 5cm only.SA 125
                             Project number = 000971-Y5   08:36 Friday, March 9, 2018

                                  The GLM Procedure

             Level of                      -----------Ca5Depth----------
             GrassLandCommunity      N             Mean          Std Dev

             GM11                   36         8.388889       26.4395128
             Gh15                   35       136.967714       68.7690144


               Level of                 -----------Ca5Depth----------
               SlopePosition      N             Mean          Std Dev

               Bottom            24       66.1670833       78.7202839
               Mid               24       71.5570833       81.7784480
               Top               23       77.8473913       90.6298328


    Level of               Level of                 -----------Ca5Depth----------
    GrassLandCommunity     SlopePosition      N             Mean          Std Dev

    GM11                   Bottom            12         5.830833       12.3098464
    GM11                   Mid               12         4.725833        8.8460412
    GM11                   Top               12        14.610000       43.9370167
    Gh15                   Bottom            12       126.503333       69.7334810
    Gh15                   Mid               12       138.388333       64.4999467
    Gh15                   Top               11       146.833636       76.8960696

  Gilbert Pule: Experiment 2 data for statistical analysisNo Outliers 5cm only.SA 126
                             Project number = 000971-Y5   08:36 Friday, March 9, 2018

                                  The GLM Procedure

                             t Tests (LSD) for Ca5Depth

       NOTE: This test controls the Type I comparisonwise error rate, not the
                             experimentwise error rate.


                        Alpha                            0.05
                        Error Degrees of Freedom           65
                        Error Mean Square            2802.243
                        Critical Value of t           1.99714
                        Least Significant Difference   25.096
                        Harmonic Mean of Cell Sizes  35.49296

                           NOTE: Cell sizes are not equal.


             Means with the same letter are not significantly different.


                                                   Grass
                                                   Land
                t Grouping          Mean      N    Community

                         A        136.97     35    Gh15

                         B          8.39     36    GM11

  Gilbert Pule: Experiment 2 data for statistical analysisNo Outliers 5cm only.SA 127
                             Project number = 000971-Y5   08:36 Friday, March 9, 2018

                                  The GLM Procedure

                             t Tests (LSD) for Ca5Depth

       NOTE: This test controls the Type I comparisonwise error rate, not the
                             experimentwise error rate.


                        Alpha                            0.05
                        Error Degrees of Freedom           65
                        Error Mean Square            2802.243
                        Critical Value of t           1.99714
                        Least Significant Difference   30.739
                        Harmonic Mean of Cell Sizes  23.65714

                           NOTE: Cell sizes are not equal.


             Means with the same letter are not significantly different.


                                                   Slope
                t Grouping          Mean      N    Position

                         A         77.85     23    Top
                         A
                         A         71.56     24    Mid
                         A
                         A         66.17     24    Bottom

  Gilbert Pule: Experiment 2 data for statistical analysisNo Outliers 5cm only.SA 128
                             Project number = 000971-Y5   08:36 Friday, March 9, 2018

                                  The GLM Procedure

                           Dependent Variable: Mg5Depth

                                         Sum of
 Source                      DF         Squares     Mean Square    F Value    Pr > F

 Model                        5     20508.67550      4101.73510      11.64    <.0001

 Error                       64     22554.00868       352.40639

 Corrected Total             69     43062.68418


                R-Square     Coeff Var      Root MSE    Mg5Depth Mean

                0.476252      58.76214      18.77249         31.94657


 Source                      DF       Type I SS     Mean Square    F Value    Pr > F

 GrassLandCommunity           1     19549.38724     19549.38724      55.47    <.0001
 SlopePosition                2       397.64445       198.82222       0.56    0.5716
 GrassLand*SlopePosit         2       561.64381       280.82190       0.80    0.4552

  Gilbert Pule: Experiment 2 data for statistical analysisNo Outliers 5cm only.SA 129
                             Project number = 000971-Y5   08:36 Friday, March 9, 2018

                                  The GLM Procedure

             Level of                      -----------Mg5Depth----------
             GrassLandCommunity      N             Mean          Std Dev

             GM11                   36       15.7058333        9.5841358
             Gh15                   34       49.1426471       24.8012395


               Level of                 -----------Mg5Depth----------
               SlopePosition      N             Mean          Std Dev

               Bottom            23       28.2995652       21.2495847
               Mid               24       33.8783333       26.1174344
               Top               23       33.5778261       27.7842745


    Level of               Level of                 -----------Mg5Depth----------
    GrassLandCommunity     SlopePosition      N             Mean          Std Dev

    GM11                   Bottom            12       16.1525000        5.7877663
    GM11                   Mid               12       15.7883333        9.8032498
    GM11                   Top               12       15.1766667       12.7332445
    Gh15                   Bottom            11       41.5509091       24.2142778
    Gh15                   Mid               12       51.9683333       24.8224121
    Gh15                   Top               11       53.6518182       25.8919110

  Gilbert Pule: Experiment 2 data for statistical analysisNo Outliers 5cm only.SA 130
                             Project number = 000971-Y5   08:36 Friday, March 9, 2018

                                  The GLM Procedure

                             t Tests (LSD) for Mg5Depth

       NOTE: This test controls the Type I comparisonwise error rate, not the
                             experimentwise error rate.


                        Alpha                            0.05
                        Error Degrees of Freedom           64
                        Error Mean Square            352.4064
                        Critical Value of t           1.99773
                        Least Significant Difference   8.9684
                        Harmonic Mean of Cell Sizes  34.97143

                           NOTE: Cell sizes are not equal.


             Means with the same letter are not significantly different.


                                                   Grass
                                                   Land
                t Grouping          Mean      N    Community

                         A        49.143     34    Gh15

                         B        15.706     36    GM11

  Gilbert Pule: Experiment 2 data for statistical analysisNo Outliers 5cm only.SA 131
                             Project number = 000971-Y5   08:36 Friday, March 9, 2018

                                  The GLM Procedure

                             t Tests (LSD) for Mg5Depth

       NOTE: This test controls the Type I comparisonwise error rate, not the
                             experimentwise error rate.


                        Alpha                            0.05
                        Error Degrees of Freedom           64
                        Error Mean Square            352.4064
                        Critical Value of t           1.99773
                        Least Significant Difference   10.982
                        Harmonic Mean of Cell Sizes  23.32394

                           NOTE: Cell sizes are not equal.


             Means with the same letter are not significantly different.


                                                   Slope
                t Grouping          Mean      N    Position

                         A        33.878     24    Mid
                         A
                         A        33.578     23    Top
                         A
                         A        28.300     23    Bottom

  Gilbert Pule: Experiment 2 data for statistical analysisNo Outliers 5cm only.SA 132
                             Project number = 000971-Y5   08:36 Friday, March 9, 2018

                                  The GLM Procedure

                         Dependent Variable: TotalN5Depth

                                         Sum of
 Source                      DF         Squares     Mean Square    F Value    Pr > F

 Model                        5      0.00330775      0.00066155       8.60    <.0001

 Error                       65      0.00499735      0.00007688

 Corrected Total             70      0.00830510


              R-Square     Coeff Var      Root MSE    TotalN5Depth Mean

              0.398279      14.83312      0.008768             0.059113


 Source                      DF       Type I SS     Mean Square    F Value    Pr > F

 GrassLandCommunity           1      0.00280077      0.00280077      36.43    <.0001
 SlopePosition                2      0.00027088      0.00013544       1.76    0.1799
 GrassLand*SlopePosit         2      0.00023610      0.00011805       1.54    0.2231

  Gilbert Pule: Experiment 2 data for statistical analysisNo Outliers 5cm only.SA 133
                             Project number = 000971-Y5   08:36 Friday, March 9, 2018

                                  The GLM Procedure

             Level of                      ---------TotalN5Depth--------
             GrassLandCommunity      N             Mean          Std Dev

             GM11                   35       0.05274286       0.00655449
             Gh15                   36       0.06530556       0.01074861


               Level of                 ---------TotalN5Depth--------
               SlopePosition      N             Mean          Std Dev

               Bottom            24       0.06175000       0.00960638
               Mid               24       0.05745833       0.01184677
               Top               23       0.05808696       0.01107377


    Level of               Level of                 ---------TotalN5Depth--------
    GrassLandCommunity     SlopePosition      N             Mean          Std Dev

    GM11                   Bottom            12       0.05800000       0.00691507
    GM11                   Mid               12       0.04975000       0.00511904
    GM11                   Top               11       0.05027273       0.00374409
    Gh15                   Bottom            12       0.06550000       0.01069834
    Gh15                   Mid               12       0.06516667       0.01173056
    Gh15                   Top               12       0.06525000       0.01074604

  Gilbert Pule: Experiment 2 data for statistical analysisNo Outliers 5cm only.SA 134
                             Project number = 000971-Y5   08:36 Friday, March 9, 2018

                                  The GLM Procedure

                           t Tests (LSD) for TotalN5Depth

       NOTE: This test controls the Type I comparisonwise error rate, not the
                             experimentwise error rate.


                        Alpha                            0.05
                        Error Degrees of Freedom           65
                        Error Mean Square            0.000077
                        Critical Value of t           1.99714
                        Least Significant Difference   0.0042
                        Harmonic Mean of Cell Sizes  35.49296

                           NOTE: Cell sizes are not equal.


             Means with the same letter are not significantly different.


                                                   Grass
                                                   Land
                t Grouping          Mean      N    Community

                         A      0.065306     36    Gh15

                         B      0.052743     35    GM11

  Gilbert Pule: Experiment 2 data for statistical analysisNo Outliers 5cm only.SA 135
                             Project number = 000971-Y5   08:36 Friday, March 9, 2018

                                  The GLM Procedure

                           t Tests (LSD) for TotalN5Depth

       NOTE: This test controls the Type I comparisonwise error rate, not the
                             experimentwise error rate.


                        Alpha                            0.05
                        Error Degrees of Freedom           65
                        Error Mean Square            0.000077
                        Critical Value of t           1.99714
                        Least Significant Difference   0.0051
                        Harmonic Mean of Cell Sizes  23.65714

                           NOTE: Cell sizes are not equal.


             Means with the same letter are not significantly different.


                                                   Slope
                t Grouping          Mean      N    Position

                         A      0.061750     24    Bottom
                         A
                         A      0.058087     23    Top
                         A
                         A      0.057458     24    Mid

  Gilbert Pule: Experiment 2 data for statistical analysisNo Outliers 5cm only.SA 136
                             Project number = 000971-Y5   08:36 Friday, March 9, 2018

                                  The GLM Procedure

                              Class Level Information

            Class                   Levels    Values

            Farm                         2    Kaalfontein Schietfontein

            GrassLandCommunity           2    GM11 Gh15

            Rainfall                     2    593 654

            SlopePosition                3    Bottom Mid Top


                            Data for Analysis of Altitude

                       Number of Observations Read          72
                       Number of Observations Used          69


                       Data for Analysis of Longitude Latitude

                       Number of Observations Read          72
                       Number of Observations Used          70


                           Data for Analysis of SplumDens
                        SplumMeanConCov IndivIntercep K5Depth
                             Org_C5Depth SandPers5Depth
                         SiltPers5Depth P5Depth pHH2O5Depth

                       Number of Observations Read          72
                       Number of Observations Used          72


                         Data for Analysis of BulkDens5Depth

                       Number of Observations Read          72
                       Number of Observations Used          69


                            Data for Analysis of Na5Dpth

                       Number of Observations Read          72
                       Number of Observations Used          68


                            Data for Analysis of Ca5Depth

                       Number of Observations Read          72
                       Number of Observations Used          71


                            Data for Analysis of Mg5Depth

                       Number of Observations Read          72
                       Number of Observations Used          70


                          Data for Analysis of TotalN5Depth

                       Number of Observations Read          72
                       Number of Observations Used          71


NOTE: Variables in each group are consistent with respect to the presence or absence
      of missing values.

  Gilbert Pule: Experiment 2 data for statistical analysisNo Outliers 5cm only.SA 137
                             Project number = 000971-Y5   08:36 Friday, March 9, 2018

                                  The GLM Procedure

                           Dependent Variable: Altitude

                                         Sum of
 Source                      DF         Squares     Mean Square    F Value    Pr > F

 Model                        5     424758.6691      84951.7338    3914.41    <.0001

 Error                       63       1367.2439         21.7023

 Corrected Total             68     426125.9130


                R-Square     Coeff Var      Root MSE    Altitude Mean

                0.996791      0.302667      4.658571         1539.174


 Source                      DF       Type I SS     Mean Square    F Value    Pr > F

 Rainfall                     1     424582.6302     424582.6302    19564.0    <.0001
 SlopePosition                2        174.5427         87.2714       4.02    0.0227
 Rainfall*SlopePositi         2          1.4962          0.7481       0.03    0.9661

  Gilbert Pule: Experiment 2 data for statistical analysisNo Outliers 5cm only.SA 138
                             Project number = 000971-Y5   08:36 Friday, March 9, 2018

                                  The GLM Procedure

                  Level of            -----------Altitude----------
                  Rainfall      N             Mean          Std Dev

                  593          36       1614.27778       4.70021952
                  654          33       1457.24242       4.90554726


               Level of                 -----------Altitude----------
               SlopePosition      N             Mean          Std Dev

               Bottom            24       1533.87500       80.4445867
               Mid               23       1539.21739       80.0982105
               Top               22       1544.90909       80.1016670


         Level of     Level of                 -----------Altitude----------
         Rainfall     SlopePosition      N             Mean          Std Dev

         593          Bottom            12       1612.50000       4.35889894
         593          Mid               12       1614.08333       4.96273996
         593          Top               12       1616.25000       4.35107092
         654          Bottom            12       1455.25000       4.91981153
         654          Mid               11       1457.54545       4.92673597
         654          Top               10       1459.30000       4.34741302

  Gilbert Pule: Experiment 2 data for statistical analysisNo Outliers 5cm only.SA 139
                             Project number = 000971-Y5   08:36 Friday, March 9, 2018

                                  The GLM Procedure

                             t Tests (LSD) for Altitude

       NOTE: This test controls the Type I comparisonwise error rate, not the
                             experimentwise error rate.


                        Alpha                            0.05
                        Error Degrees of Freedom           63
                        Error Mean Square            21.70228
                        Critical Value of t           1.99834
                        Least Significant Difference   2.2436
                        Harmonic Mean of Cell Sizes  34.43478

                           NOTE: Cell sizes are not equal.


             Means with the same letter are not significantly different.


                t Grouping          Mean      N    Rainfall

                         A      1614.278     36    593

                         B      1457.242     33    654

  Gilbert Pule: Experiment 2 data for statistical analysisNo Outliers 5cm only.SA 140
                             Project number = 000971-Y5   08:36 Friday, March 9, 2018

                                  The GLM Procedure

                             t Tests (LSD) for Altitude

       NOTE: This test controls the Type I comparisonwise error rate, not the
                             experimentwise error rate.


                        Alpha                            0.05
                        Error Degrees of Freedom           63
                        Error Mean Square            21.70228
                        Critical Value of t           1.99834
                        Least Significant Difference   2.7469
                        Harmonic Mean of Cell Sizes    22.971

                           NOTE: Cell sizes are not equal.


             Means with the same letter are not significantly different.


                                                   Slope
                t Grouping          Mean      N    Position

                         A      1544.909     22    Top

                         B      1539.217     23    Mid

                         C      1533.875     24    Bottom

  Gilbert Pule: Experiment 2 data for statistical analysisNo Outliers 5cm only.SA 141
                             Project number = 000971-Y5   08:36 Friday, March 9, 2018

                                  The GLM Procedure

                          Dependent Variable: Longitude

                                         Sum of
 Source                      DF         Squares     Mean Square    F Value    Pr > F

 Model                        5      1.71641004      0.34328201    59595.2    <.0001

 Error                       64      0.00036865      0.00000576

 Corrected Total             69      1.71677869


               R-Square     Coeff Var      Root MSE    Longitude Mean

               0.999785      0.009256      0.002400          25.93067


 Source                      DF       Type I SS     Mean Square    F Value    Pr > F

 Rainfall                     1      1.71637520      1.71637520     297970    <.0001
 SlopePosition                2      0.00000822      0.00000411       0.71    0.4937
 Rainfall*SlopePositi         2      0.00002661      0.00001331       2.31    0.1075

  Gilbert Pule: Experiment 2 data for statistical analysisNo Outliers 5cm only.SA 142
                             Project number = 000971-Y5   08:36 Friday, March 9, 2018

                                  The GLM Procedure

                           Dependent Variable: Latitude

                                         Sum of
 Source                      DF         Squares     Mean Square    F Value    Pr > F

 Model                        5     25.79075174      5.15815035    3659825    <.0001

 Error                       64      0.00009020      0.00000141

 Corrected Total             69     25.79084195


                R-Square     Coeff Var      Root MSE    Latitude Mean

                0.999997      0.004231      0.001187         28.05684


 Source                      DF       Type I SS     Mean Square    F Value    Pr > F

 Rainfall                     1     25.79074933     25.79074933     1.83E7    <.0001
 SlopePosition                2      0.00000130      0.00000065       0.46    0.6317
 Rainfall*SlopePositi         2      0.00000111      0.00000056       0.39    0.6760

  Gilbert Pule: Experiment 2 data for statistical analysisNo Outliers 5cm only.SA 143
                             Project number = 000971-Y5   08:36 Friday, March 9, 2018

                                  The GLM Procedure

 Level of            ----------Longitude----------     -----------Latitude----------
 Rainfall      N             Mean          Std Dev             Mean          Std Dev

 593          36       26.0828506       0.00335588       27.4669514       0.00097073
 654          34       25.7695476       0.00053149       28.6814312       0.00134429


 Level of               ----------Longitude---------    ----------Latitude----------
 SlopePosition     N            Mean         Std Dev            Mean         Std Dev

 Bottom           24      25.9265175      0.16092259      28.0740496      0.62048501
 Mid              24      25.9257329      0.15949875      28.0741588      0.62021343
 Top              22      25.9406014      0.15929659      28.0191777      0.61887007


 Level of  Level of           ---------Longitude--------  ---------Latitude---------
 Rainfall  SlopePosition   N          Mean       Std Dev          Mean       Std Dev

 593       Bottom         12    26.0840142    0.00507767    27.4666300    0.00098021
 593       Mid            12    26.0818717    0.00105859    27.4670050    0.00099145
 593       Top            12    26.0826658    0.00252396    27.4672192    0.00092761
 654       Bottom         12    25.7690208    0.00036340    28.6814692    0.00141627
 654       Mid            12    25.7695942    0.00027665    28.6813125    0.00138389
 654       Top            10    25.7701240    0.00019699    28.6815280    0.00134264

  Gilbert Pule: Experiment 2 data for statistical analysisNo Outliers 5cm only.SA 144
                             Project number = 000971-Y5   08:36 Friday, March 9, 2018

                                  The GLM Procedure

                             t Tests (LSD) for Longitude

       NOTE: This test controls the Type I comparisonwise error rate, not the
                             experimentwise error rate.


                        Alpha                            0.05
                        Error Degrees of Freedom           64
                        Error Mean Square             5.76E-6
                        Critical Value of t           1.99773
                        Least Significant Difference   0.0011
                        Harmonic Mean of Cell Sizes  34.97143

                           NOTE: Cell sizes are not equal.


             Means with the same letter are not significantly different.


                t Grouping          Mean      N    Rainfall

                         A     26.082851     36    593

                         B     25.769548     34    654

  Gilbert Pule: Experiment 2 data for statistical analysisNo Outliers 5cm only.SA 145
                             Project number = 000971-Y5   08:36 Friday, March 9, 2018

                                  The GLM Procedure

                             t Tests (LSD) for Latitude

       NOTE: This test controls the Type I comparisonwise error rate, not the
                             experimentwise error rate.


                        Alpha                            0.05
                        Error Degrees of Freedom           64
                        Error Mean Square            1.409E-6
                        Critical Value of t           1.99773
                        Least Significant Difference   0.0006
                        Harmonic Mean of Cell Sizes  34.97143

                           NOTE: Cell sizes are not equal.


             Means with the same letter are not significantly different.


                t Grouping          Mean      N    Rainfall

                         A     28.681431     34    654

                         B     27.466951     36    593

  Gilbert Pule: Experiment 2 data for statistical analysisNo Outliers 5cm only.SA 146
                             Project number = 000971-Y5   08:36 Friday, March 9, 2018

                                  The GLM Procedure

                             t Tests (LSD) for Longitude

       NOTE: This test controls the Type I comparisonwise error rate, not the
                             experimentwise error rate.


                        Alpha                            0.05
                        Error Degrees of Freedom           64
                        Error Mean Square             5.76E-6
                        Critical Value of t           1.99773
                        Least Significant Difference   0.0014
                        Harmonic Mean of Cell Sizes  23.29412

                           NOTE: Cell sizes are not equal.


             Means with the same letter are not significantly different.


                                                   Slope
                t Grouping          Mean      N    Position

                         A     25.940601     22    Top

                         B     25.926518     24    Bottom
                         B
                         B     25.925733     24    Mid

  Gilbert Pule: Experiment 2 data for statistical analysisNo Outliers 5cm only.SA 147
                             Project number = 000971-Y5   08:36 Friday, March 9, 2018

                                  The GLM Procedure

                             t Tests (LSD) for Latitude

       NOTE: This test controls the Type I comparisonwise error rate, not the
                             experimentwise error rate.


                        Alpha                            0.05
                        Error Degrees of Freedom           64
                        Error Mean Square            1.409E-6
                        Critical Value of t           1.99773
                        Least Significant Difference   0.0007
                        Harmonic Mean of Cell Sizes  23.29412

                           NOTE: Cell sizes are not equal.


             Means with the same letter are not significantly different.


                                                   Slope
                t Grouping          Mean      N    Position

                         A     28.074159     24    Mid
                         A
                         A     28.074050     24    Bottom

                         B     28.019178     22    Top

  Gilbert Pule: Experiment 2 data for statistical analysisNo Outliers 5cm only.SA 148
                             Project number = 000971-Y5   08:36 Friday, March 9, 2018

                                  The GLM Procedure

                          Dependent Variable: SplumDens

                                         Sum of
 Source                      DF         Squares     Mean Square    F Value    Pr > F

 Model                        5     12702.56944      2540.51389       7.64    <.0001

 Error                       66     21945.08333       332.50126

 Corrected Total             71     34647.65278


               R-Square     Coeff Var      Root MSE    SplumDens Mean

               0.366621      57.55776      18.23462          31.68056


 Source                      DF       Type I SS     Mean Square    F Value    Pr > F

 Rainfall                     1     5356.125000     5356.125000      16.11    0.0002
 SlopePosition                2     2638.361111     1319.180556       3.97    0.0236
 Rainfall*SlopePositi         2     4708.083333     2354.041667       7.08    0.0016

  Gilbert Pule: Experiment 2 data for statistical analysisNo Outliers 5cm only.SA 149
                             Project number = 000971-Y5   08:36 Friday, March 9, 2018

                                  The GLM Procedure

                       Dependent Variable: SplumMeanConCov

                                         Sum of
 Source                      DF         Squares     Mean Square    F Value    Pr > F

 Model                        5      4.34661129      0.86932226       2.65    0.0301

 Error                       66     21.61064058      0.32743395

 Corrected Total             71     25.95725188


            R-Square     Coeff Var      Root MSE    SplumMeanConCov Mean

            0.167453      46.43845      0.572218                1.232208


 Source                      DF       Type I SS     Mean Square    F Value    Pr > F

 Rainfall                     1      1.90808112      1.90808112       5.83    0.0186
 SlopePosition                2      2.40637758      1.20318879       3.67    0.0307
 Rainfall*SlopePositi         2      0.03215258      0.01607629       0.05    0.9521

  Gilbert Pule: Experiment 2 data for statistical analysisNo Outliers 5cm only.SA 150
                             Project number = 000971-Y5   08:36 Friday, March 9, 2018

                                  The GLM Procedure

                        Dependent Variable: IndivIntercep

                                         Sum of
 Source                      DF         Squares     Mean Square    F Value    Pr > F

 Model                        5      388.777778       77.755556       2.13    0.0728

 Error                       66     2410.500000       36.522727

 Corrected Total             71     2799.277778


             R-Square     Coeff Var      Root MSE    IndivIntercep Mean

             0.138885      53.45517      6.043404              11.30556


 Source                      DF       Type I SS     Mean Square    F Value    Pr > F

 Rainfall                     1     180.5000000     180.5000000       4.94    0.0296
 SlopePosition                2      50.0277778      25.0138889       0.68    0.5077
 Rainfall*SlopePositi         2     158.2500000      79.1250000       2.17    0.1227

  Gilbert Pule: Experiment 2 data for statistical analysisNo Outliers 5cm only.SA 151
                             Project number = 000971-Y5   08:36 Friday, March 9, 2018

                                  The GLM Procedure

                           Dependent Variable: K5Depth

                                         Sum of
 Source                      DF         Squares     Mean Square    F Value    Pr > F

 Model                        5      71473.0866      14294.6173      10.83    <.0001

 Error                       66      87074.6859       1319.3134

 Corrected Total             71     158547.7725


                R-Square     Coeff Var      Root MSE    K5Depth Mean

                0.450798      43.30577      36.32235        83.87417


 Source                      DF       Type I SS     Mean Square    F Value    Pr > F

 Rainfall                     1     69946.72694     69946.72694      53.02    <.0001
 SlopePosition                2       101.82563        50.91282       0.04    0.9622
 Rainfall*SlopePositi         2      1424.53404       712.26702       0.54    0.5854

  Gilbert Pule: Experiment 2 data for statistical analysisNo Outliers 5cm only.SA 152
                             Project number = 000971-Y5   08:36 Friday, March 9, 2018

                                  The GLM Procedure

                         Dependent Variable: Org_C5Depth

                                         Sum of
 Source                      DF         Squares     Mean Square    F Value    Pr > F

 Model                        5     10.56347917      2.11269583      33.74    <.0001

 Error                       66      4.13260833      0.06261528

 Corrected Total             71     14.69608750


              R-Square     Coeff Var      Root MSE    Org_C5Depth Mean

              0.718795      21.87807      0.250230            1.143750


 Source                      DF       Type I SS     Mean Square    F Value    Pr > F

 Rainfall                     1     10.29823472     10.29823472     164.47    <.0001
 SlopePosition                2      0.16043333      0.08021667       1.28    0.2845
 Rainfall*SlopePositi         2      0.10481111      0.05240556       0.84    0.4376

  Gilbert Pule: Experiment 2 data for statistical analysisNo Outliers 5cm only.SA 153
                             Project number = 000971-Y5   08:36 Friday, March 9, 2018

                                  The GLM Procedure

                        Dependent Variable: SandPers5Depth

                                         Sum of
 Source                      DF         Squares     Mean Square    F Value    Pr > F

 Model                        5     9530.000000     1906.000000     898.54    <.0001

 Error                       66      140.000000        2.121212

 Corrected Total             71     9670.000000


             R-Square     Coeff Var      Root MSE    SandPers5Depth Mean

             0.985522      1.946243      1.456438               74.83333


 Source                      DF       Type I SS     Mean Square    F Value    Pr > F

 Rainfall                     1     9522.000000     9522.000000    4488.94    <.0001
 SlopePosition                2        4.000000        2.000000       0.94    0.3947
 Rainfall*SlopePositi         2        4.000000        2.000000       0.94    0.3947

  Gilbert Pule: Experiment 2 data for statistical analysisNo Outliers 5cm only.SA 154
                             Project number = 000971-Y5   08:36 Friday, March 9, 2018

                                  The GLM Procedure

                        Dependent Variable: SiltPers5Depth

                                         Sum of
 Source                      DF         Squares     Mean Square    F Value    Pr > F

 Model                        5     680.0000000     136.0000000     280.50    <.0001

 Error                       66      32.0000000       0.4848485

 Corrected Total             71     712.0000000


             R-Square     Coeff Var      Root MSE    SiltPers5Depth Mean

             0.955056      12.28783      0.696311               5.666667


 Source                      DF       Type I SS     Mean Square    F Value    Pr > F

 Rainfall                     1     648.0000000     648.0000000    1336.50    <.0001
 SlopePosition                2      16.0000000       8.0000000      16.50    <.0001
 Rainfall*SlopePositi         2      16.0000000       8.0000000      16.50    <.0001

  Gilbert Pule: Experiment 2 data for statistical analysisNo Outliers 5cm only.SA 155
                             Project number = 000971-Y5   08:36 Friday, March 9, 2018

                                  The GLM Procedure

                           Dependent Variable: P5Depth

                                         Sum of
 Source                      DF         Squares     Mean Square    F Value    Pr > F

 Model                        5     171.9289174      34.3857835      20.77    <.0001

 Error                       66     109.2618992       1.6554833

 Corrected Total             71     281.1908166


                R-Square     Coeff Var      Root MSE    P5Depth Mean

                0.611431      48.85468      1.286656        2.633639


 Source                      DF       Type I SS     Mean Square    F Value    Pr > F

 Rainfall                     1     162.5524702     162.5524702      98.19    <.0001
 SlopePosition                2       4.1582487       2.0791243       1.26    0.2915
 Rainfall*SlopePositi         2       5.2181985       2.6090993       1.58    0.2145

  Gilbert Pule: Experiment 2 data for statistical analysisNo Outliers 5cm only.SA 156
                             Project number = 000971-Y5   08:36 Friday, March 9, 2018

                                  The GLM Procedure

                         Dependent Variable: pHH2O5Depth

                                         Sum of
 Source                      DF         Squares     Mean Square    F Value    Pr > F

 Model                        5      0.28921111      0.05784222       1.26    0.2896

 Error                       66      3.01810000      0.04572879

 Corrected Total             71      3.30731111


              R-Square     Coeff Var      Root MSE    pHH2O5Depth Mean

              0.087446      4.378537      0.213843            4.883889


 Source                      DF       Type I SS     Mean Square    F Value    Pr > F

 Rainfall                     1      0.01445000      0.01445000       0.32    0.5759
 SlopePosition                2      0.00923611      0.00461806       0.10    0.9041
 Rainfall*SlopePositi         2      0.26552500      0.13276250       2.90    0.0619

  Gilbert Pule: Experiment 2 data for statistical analysisNo Outliers 5cm only.SA 157
                             Project number = 000971-Y5   08:36 Friday, March 9, 2018

                                  The GLM Procedure

 Level of            ----------SplumDens----------     -------SplumMeanConCov-------
 Rainfall      N             Mean          Std Dev             Mean          Std Dev

 593          36       23.0555556       14.2867460       1.39500000       0.64581510
 654          36       40.3055556       25.1553112       1.06941667       0.51965568

 Level of            --------IndivIntercep--------     -----------K5Depth-----------
 Rainfall      N             Mean          Std Dev             Mean          Std Dev

 593          36        9.7222222       4.60193196       115.042778       42.5562015
 654          36       12.8888889       7.32423678        52.705556       26.8407928

 Level of            ---------Org_C5Depth---------     --------SandPers5Depth-------
 Rainfall      N             Mean          Std Dev             Mean          Std Dev

 593          36       1.52194444       0.28723131       63.3333333       1.91236577
 654          36       0.76555556       0.20772846       86.3333333       0.75592895

 Level of            --------SiltPers5Depth-------     -----------P5Depth-----------
 Rainfall      N             Mean          Std Dev             Mean          Std Dev

 593          36       8.66666667       0.95618289       4.13619444       1.56901432
 654          36       2.66666667       0.95618289       1.13108333       0.96325547

                  Level of            ---------pHH2O5Depth---------
                  Rainfall      N             Mean          Std Dev

                  593          36       4.86972222       0.23071920
                  654          36       4.89805556       0.20211481


 Level of               ----------SplumDens---------    -------SplumMeanConCov------
 SlopePosition     N            Mean         Std Dev            Mean         Std Dev

 Bottom           24      39.3750000      29.6703812      0.98612500      0.57687135
 Mid              24      31.0833333      17.6756198      1.28658333      0.54763062
 Top              24      24.5833333      14.1049380      1.42391667      0.62551675

 Level of               --------IndivIntercep-------    -----------K5Depth----------
 SlopePosition     N            Mean         Std Dev            Mean         Std Dev

 Bottom           24      12.3333333      7.64236855      84.1675000      53.6082625
 Mid              24      11.2916667      5.98170520      85.1616667      42.3465312
 Top              24      10.2916667      5.03448254      82.2933333      47.1368190

 Level of               ---------Org_C5Depth--------    -------SandPers5Depth-------
 SlopePosition     N            Mean         Std Dev            Mean         Std Dev

 Bottom           24      1.16708333      0.40081959      74.6666667      11.6569318
 Mid              24      1.07791667      0.43359949      74.6666667      11.6569318
 Top              24      1.18625000      0.53227822      75.1666667      12.1857603

 Level of               -------SiltPers5Depth-------    -----------P5Depth----------
 SlopePosition     N            Mean         Std Dev            Mean         Std Dev

 Bottom           24      6.33333333      2.47889644      2.68333333      2.25091221
 Mid              24      5.33333333      3.47245893      2.31762500      1.56425998
 Top              24      5.33333333      3.47245893      2.89995833      2.12870364

                Level of               ---------pHH2O5Depth---------
                SlopePosition     N            Mean          Std Dev

                Bottom           24      4.89916667       0.21449469
                Mid              24      4.88041667       0.21786472
                Top              24      4.87208333       0.22343133


 Level of  Level of           ---------SplumDens--------  ------SplumMeanConCov-----
 Rainfall  SlopePosition   N          Mean       Std Dev          Mean       Std Dev

 593       Bottom         12    19.7500000    10.4196056    1.14083333    0.48690970
 593       Mid            12    25.2500000    17.1258709    1.47833333    0.54825066
 593       Top            12    24.1666667    15.1347483    1.56583333    0.82432847
 654       Bottom         12    59.0000000    29.8633250    0.83141667    0.63758272
 654       Mid            12    36.9166667    16.9032452    1.09483333    0.49624807
 654       Top            12    25.0000000    13.6581644    1.28200000    0.30765654

 Level of  Level of           -------IndivIntercep------  ----------K5Depth---------
 Rainfall  SlopePosition   N          Mean       Std Dev          Mean       Std Dev

 593       Bottom         12     9.0000000    4.06761042    109.951667    57.0722282
 593       Mid            12     9.5833333    5.03548018    116.205833    34.7511354
 593       Top            12    10.5833333    4.90747729    118.970833    35.3681319
 654       Bottom         12    15.6666667    9.01849951     58.383333    36.0719786
 654       Mid            12    13.0000000    6.56436662     54.117500    20.9554459
 654       Top            12    10.0000000    5.35978290     45.615833    21.4424064

 Level of  Level of           --------Org_C5Depth-------  ------SandPers5Depth------
 Rainfall  SlopePosition   N          Mean       Std Dev          Mean       Std Dev

 593       Bottom         12    1.50000000    0.23977262    63.3333333    1.96946386
 593       Mid            12    1.45333333    0.25748198    63.3333333    1.96946386
 593       Top            12    1.61250000    0.35229443    63.3333333    1.96946386
 654       Bottom         12    0.83416667    0.19133304    86.0000000    0.00000000
 654       Mid            12    0.70250000    0.13896533    86.0000000    0.00000000
 654       Top            12    0.76000000    0.26809089    87.0000000    1.04446594

 Level of  Level of           ------SiltPers5Depth------  ----------P5Depth---------
 Rainfall  SlopePosition   N          Mean       Std Dev          Mean       Std Dev

 593       Bottom         12    8.66666667    0.98473193    4.34500000    1.67832438
 593       Mid            12    8.66666667    0.98473193    3.44108333    1.17224784
 593       Top            12    8.66666667    0.98473193    4.62250000    1.67613259
 654       Bottom         12    4.00000000    0.00000000    1.02166667    1.32391179
 654       Mid            12    2.00000000    0.00000000    1.19416667    0.99413241
 654       Top            12    2.00000000    0.00000000    1.17741667    0.43760722

             Level of  Level of           ---------pHH2O5Depth---------
             Rainfall  SlopePosition   N          Mean          Std Dev

             593       Bottom         12    4.82416667       0.24343968
             593       Mid            12    4.84416667       0.20192070
             593       Top            12    4.94083333       0.24666718
             654       Bottom         12    4.97416667       0.15704419
             654       Mid            12    4.91666667       0.23580937
             654       Top            12    4.80333333       0.18227518

  Gilbert Pule: Experiment 2 data for statistical analysisNo Outliers 5cm only.SA 158
                             Project number = 000971-Y5   08:36 Friday, March 9, 2018

                                  The GLM Procedure

                             t Tests (LSD) for SplumDens

       NOTE: This test controls the Type I comparisonwise error rate, not the
                             experimentwise error rate.


                        Alpha                            0.05
                        Error Degrees of Freedom           66
                        Error Mean Square            332.5013
                        Critical Value of t           1.99656
                        Least Significant Difference   8.5811


             Means with the same letter are not significantly different.


                t Grouping          Mean      N    Rainfall

                         A        40.306     36    654

                         B        23.056     36    593

  Gilbert Pule: Experiment 2 data for statistical analysisNo Outliers 5cm only.SA 159
                             Project number = 000971-Y5   08:36 Friday, March 9, 2018

                                  The GLM Procedure

                          t Tests (LSD) for SplumMeanConCov

       NOTE: This test controls the Type I comparisonwise error rate, not the
                             experimentwise error rate.


                        Alpha                            0.05
                        Error Degrees of Freedom           66
                        Error Mean Square            0.327434
                        Critical Value of t           1.99656
                        Least Significant Difference   0.2693


             Means with the same letter are not significantly different.


                t Grouping          Mean      N    Rainfall

                         A        1.3950     36    593

                         B        1.0694     36    654

  Gilbert Pule: Experiment 2 data for statistical analysisNo Outliers 5cm only.SA 160
                             Project number = 000971-Y5   08:36 Friday, March 9, 2018

                                  The GLM Procedure

                           t Tests (LSD) for IndivIntercep

       NOTE: This test controls the Type I comparisonwise error rate, not the
                             experimentwise error rate.


                        Alpha                            0.05
                        Error Degrees of Freedom           66
                        Error Mean Square            36.52273
                        Critical Value of t           1.99656
                        Least Significant Difference    2.844


             Means with the same letter are not significantly different.


                t Grouping          Mean      N    Rainfall

                         A        12.889     36    654

                         B         9.722     36    593

  Gilbert Pule: Experiment 2 data for statistical analysisNo Outliers 5cm only.SA 161
                             Project number = 000971-Y5   08:36 Friday, March 9, 2018

                                  The GLM Procedure

                              t Tests (LSD) for K5Depth

       NOTE: This test controls the Type I comparisonwise error rate, not the
                             experimentwise error rate.


                        Alpha                            0.05
                        Error Degrees of Freedom           66
                        Error Mean Square            1319.313
                        Critical Value of t           1.99656
                        Least Significant Difference   17.093


             Means with the same letter are not significantly different.


                t Grouping          Mean      N    Rainfall

                         A       115.043     36    593

                         B        52.706     36    654

  Gilbert Pule: Experiment 2 data for statistical analysisNo Outliers 5cm only.SA 162
                             Project number = 000971-Y5   08:36 Friday, March 9, 2018

                                  The GLM Procedure

                            t Tests (LSD) for Org_C5Depth

       NOTE: This test controls the Type I comparisonwise error rate, not the
                             experimentwise error rate.


                        Alpha                            0.05
                        Error Degrees of Freedom           66
                        Error Mean Square            0.062615
                        Critical Value of t           1.99656
                        Least Significant Difference   0.1178


             Means with the same letter are not significantly different.


                t Grouping          Mean      N    Rainfall

                         A       1.52194     36    593

                         B       0.76556     36    654

  Gilbert Pule: Experiment 2 data for statistical analysisNo Outliers 5cm only.SA 163
                             Project number = 000971-Y5   08:36 Friday, March 9, 2018

                                  The GLM Procedure

                          t Tests (LSD) for SandPers5Depth

       NOTE: This test controls the Type I comparisonwise error rate, not the
                             experimentwise error rate.


                        Alpha                            0.05
                        Error Degrees of Freedom           66
                        Error Mean Square            2.121212
                        Critical Value of t           1.99656
                        Least Significant Difference   0.6854


             Means with the same letter are not significantly different.


                t Grouping          Mean      N    Rainfall

                         A       86.3333     36    654

                         B       63.3333     36    593

  Gilbert Pule: Experiment 2 data for statistical analysisNo Outliers 5cm only.SA 164
                             Project number = 000971-Y5   08:36 Friday, March 9, 2018

                                  The GLM Procedure

                          t Tests (LSD) for SiltPers5Depth

       NOTE: This test controls the Type I comparisonwise error rate, not the
                             experimentwise error rate.


                        Alpha                            0.05
                        Error Degrees of Freedom           66
                        Error Mean Square            0.484848
                        Critical Value of t           1.99656
                        Least Significant Difference   0.3277


             Means with the same letter are not significantly different.


                t Grouping          Mean      N    Rainfall

                         A        8.6667     36    593

                         B        2.6667     36    654

  Gilbert Pule: Experiment 2 data for statistical analysisNo Outliers 5cm only.SA 165
                             Project number = 000971-Y5   08:36 Friday, March 9, 2018

                                  The GLM Procedure

                              t Tests (LSD) for P5Depth

       NOTE: This test controls the Type I comparisonwise error rate, not the
                             experimentwise error rate.


                        Alpha                            0.05
                        Error Degrees of Freedom           66
                        Error Mean Square            1.655483
                        Critical Value of t           1.99656
                        Least Significant Difference   0.6055


             Means with the same letter are not significantly different.


                t Grouping          Mean      N    Rainfall

                         A        4.1362     36    593

                         B        1.1311     36    654

  Gilbert Pule: Experiment 2 data for statistical analysisNo Outliers 5cm only.SA 166
                             Project number = 000971-Y5   08:36 Friday, March 9, 2018

                                  The GLM Procedure

                            t Tests (LSD) for pHH2O5Depth

       NOTE: This test controls the Type I comparisonwise error rate, not the
                             experimentwise error rate.


                        Alpha                            0.05
                        Error Degrees of Freedom           66
                        Error Mean Square            0.045729
                        Critical Value of t           1.99656
                        Least Significant Difference   0.1006


             Means with the same letter are not significantly different.


                t Grouping          Mean      N    Rainfall

                         A       4.89806     36    654
                         A
                         A       4.86972     36    593

  Gilbert Pule: Experiment 2 data for statistical analysisNo Outliers 5cm only.SA 167
                             Project number = 000971-Y5   08:36 Friday, March 9, 2018

                                  The GLM Procedure

                             t Tests (LSD) for SplumDens

       NOTE: This test controls the Type I comparisonwise error rate, not the
                             experimentwise error rate.


                        Alpha                            0.05
                        Error Degrees of Freedom           66
                        Error Mean Square            332.5013
                        Critical Value of t           1.99656
                        Least Significant Difference    10.51


             Means with the same letter are not significantly different.


                                                      Slope
                   t Grouping          Mean      N    Position

                            A        39.375     24    Bottom
                            A
                       B    A        31.083     24    Mid
                       B
                       B             24.583     24    Top

  Gilbert Pule: Experiment 2 data for statistical analysisNo Outliers 5cm only.SA 168
                             Project number = 000971-Y5   08:36 Friday, March 9, 2018

                                  The GLM Procedure

                          t Tests (LSD) for SplumMeanConCov

       NOTE: This test controls the Type I comparisonwise error rate, not the
                             experimentwise error rate.


                        Alpha                            0.05
                        Error Degrees of Freedom           66
                        Error Mean Square            0.327434
                        Critical Value of t           1.99656
                        Least Significant Difference   0.3298


             Means with the same letter are not significantly different.


                                                      Slope
                   t Grouping          Mean      N    Position

                            A        1.4239     24    Top
                            A
                       B    A        1.2866     24    Mid
                       B
                       B             0.9861     24    Bottom

  Gilbert Pule: Experiment 2 data for statistical analysisNo Outliers 5cm only.SA 169
                             Project number = 000971-Y5   08:36 Friday, March 9, 2018

                                  The GLM Procedure

                           t Tests (LSD) for IndivIntercep

       NOTE: This test controls the Type I comparisonwise error rate, not the
                             experimentwise error rate.


                        Alpha                            0.05
                        Error Degrees of Freedom           66
                        Error Mean Square            36.52273
                        Critical Value of t           1.99656
                        Least Significant Difference   3.4832


             Means with the same letter are not significantly different.


                                                   Slope
                t Grouping          Mean      N    Position

                         A        12.333     24    Bottom
                         A
                         A        11.292     24    Mid
                         A
                         A        10.292     24    Top

  Gilbert Pule: Experiment 2 data for statistical analysisNo Outliers 5cm only.SA 170
                             Project number = 000971-Y5   08:36 Friday, March 9, 2018

                                  The GLM Procedure

                              t Tests (LSD) for K5Depth

       NOTE: This test controls the Type I comparisonwise error rate, not the
                             experimentwise error rate.


                        Alpha                            0.05
                        Error Degrees of Freedom           66
                        Error Mean Square            1319.313
                        Critical Value of t           1.99656
                        Least Significant Difference   20.935


             Means with the same letter are not significantly different.


                                                   Slope
                t Grouping          Mean      N    Position

                         A         85.16     24    Mid
                         A
                         A         84.17     24    Bottom
                         A
                         A         82.29     24    Top

  Gilbert Pule: Experiment 2 data for statistical analysisNo Outliers 5cm only.SA 171
                             Project number = 000971-Y5   08:36 Friday, March 9, 2018

                                  The GLM Procedure

                            t Tests (LSD) for Org_C5Depth

       NOTE: This test controls the Type I comparisonwise error rate, not the
                             experimentwise error rate.


                        Alpha                            0.05
                        Error Degrees of Freedom           66
                        Error Mean Square            0.062615
                        Critical Value of t           1.99656
                        Least Significant Difference   0.1442


             Means with the same letter are not significantly different.


                                                   Slope
                t Grouping          Mean      N    Position

                         A       1.18625     24    Top
                         A
                         A       1.16708     24    Bottom
                         A
                         A       1.07792     24    Mid

  Gilbert Pule: Experiment 2 data for statistical analysisNo Outliers 5cm only.SA 172
                             Project number = 000971-Y5   08:36 Friday, March 9, 2018

                                  The GLM Procedure

                          t Tests (LSD) for SandPers5Depth

       NOTE: This test controls the Type I comparisonwise error rate, not the
                             experimentwise error rate.


                        Alpha                            0.05
                        Error Degrees of Freedom           66
                        Error Mean Square            2.121212
                        Critical Value of t           1.99656
                        Least Significant Difference   0.8394


             Means with the same letter are not significantly different.


                                                   Slope
                t Grouping          Mean      N    Position

                         A       75.1667     24    Top
                         A
                         A       74.6667     24    Mid
                         A
                         A       74.6667     24    Bottom

  Gilbert Pule: Experiment 2 data for statistical analysisNo Outliers 5cm only.SA 173
                             Project number = 000971-Y5   08:36 Friday, March 9, 2018

                                  The GLM Procedure

                          t Tests (LSD) for SiltPers5Depth

       NOTE: This test controls the Type I comparisonwise error rate, not the
                             experimentwise error rate.


                        Alpha                            0.05
                        Error Degrees of Freedom           66
                        Error Mean Square            0.484848
                        Critical Value of t           1.99656
                        Least Significant Difference   0.4013


             Means with the same letter are not significantly different.


                                                   Slope
                t Grouping          Mean      N    Position

                         A        6.3333     24    Bottom

                         B        5.3333     24    Mid
                         B
                         B        5.3333     24    Top

  Gilbert Pule: Experiment 2 data for statistical analysisNo Outliers 5cm only.SA 174
                             Project number = 000971-Y5   08:36 Friday, March 9, 2018

                                  The GLM Procedure

                              t Tests (LSD) for P5Depth

       NOTE: This test controls the Type I comparisonwise error rate, not the
                             experimentwise error rate.


                        Alpha                            0.05
                        Error Degrees of Freedom           66
                        Error Mean Square            1.655483
                        Critical Value of t           1.99656
                        Least Significant Difference   0.7416


             Means with the same letter are not significantly different.


                                                   Slope
                t Grouping          Mean      N    Position

                         A        2.9000     24    Top
                         A
                         A        2.6833     24    Bottom
                         A
                         A        2.3176     24    Mid

  Gilbert Pule: Experiment 2 data for statistical analysisNo Outliers 5cm only.SA 175
                             Project number = 000971-Y5   08:36 Friday, March 9, 2018

                                  The GLM Procedure

                            t Tests (LSD) for pHH2O5Depth

       NOTE: This test controls the Type I comparisonwise error rate, not the
                             experimentwise error rate.


                        Alpha                            0.05
                        Error Degrees of Freedom           66
                        Error Mean Square            0.045729
                        Critical Value of t           1.99656
                        Least Significant Difference   0.1233


             Means with the same letter are not significantly different.


                                                   Slope
                t Grouping          Mean      N    Position

                         A       4.89917     24    Bottom
                         A
                         A       4.88042     24    Mid
                         A
                         A       4.87208     24    Top

  Gilbert Pule: Experiment 2 data for statistical analysisNo Outliers 5cm only.SA 176
                             Project number = 000971-Y5   08:36 Friday, March 9, 2018

                                  The GLM Procedure

                        Dependent Variable: BulkDens5Depth

                                         Sum of
 Source                      DF         Squares     Mean Square    F Value    Pr > F

 Model                        5      0.45275940      0.09055188      66.42    <.0001

 Error                       63      0.08589182      0.00136336

 Corrected Total             68      0.53865122


             R-Square     Coeff Var      Root MSE    BulkDens5Depth Mean

             0.840543      3.367484      0.036924               1.096478


 Source                      DF       Type I SS     Mean Square    F Value    Pr > F

 Rainfall                     1      0.45106469      0.45106469     330.85    <.0001
 SlopePosition                2      0.00147626      0.00073813       0.54    0.5846
 Rainfall*SlopePositi         2      0.00021845      0.00010922       0.08    0.9231

  Gilbert Pule: Experiment 2 data for statistical analysisNo Outliers 5cm only.SA 177
                             Project number = 000971-Y5   08:36 Friday, March 9, 2018

                                  The GLM Procedure

                  Level of            --------BulkDens5Depth-------
                  Rainfall      N             Mean          Std Dev

                  593          33       1.01203030       0.04074730
                  654          36       1.17388889       0.03137586


               Level of                 --------BulkDens5Depth-------
               SlopePosition      N             Mean          Std Dev

               Bottom            23       1.09160870       0.08781216
               Mid               23       1.10269565       0.09036815
               Top               23       1.09513043       0.09241030


         Level of     Level of                 --------BulkDens5Depth-------
         Rainfall     SlopePosition      N             Mean          Std Dev

         593          Bottom            11       1.00972727       0.04518870
         593          Mid               11       1.01745455       0.04558150
         593          Top               11       1.00890909       0.03383033
         654          Bottom            12       1.16666667       0.02674232
         654          Mid               12       1.18083333       0.02274696
         654          Top               12       1.17416667       0.04231018

  Gilbert Pule: Experiment 2 data for statistical analysisNo Outliers 5cm only.SA 178
                             Project number = 000971-Y5   08:36 Friday, March 9, 2018

                                  The GLM Procedure

                          t Tests (LSD) for BulkDens5Depth

       NOTE: This test controls the Type I comparisonwise error rate, not the
                             experimentwise error rate.


                        Alpha                            0.05
                        Error Degrees of Freedom           63
                        Error Mean Square            0.001363
                        Critical Value of t           1.99834
                        Least Significant Difference   0.0178
                        Harmonic Mean of Cell Sizes  34.43478

                           NOTE: Cell sizes are not equal.


             Means with the same letter are not significantly different.


                t Grouping          Mean      N    Rainfall

                         A      1.173889     36    654

                         B      1.012030     33    593

  Gilbert Pule: Experiment 2 data for statistical analysisNo Outliers 5cm only.SA 179
                             Project number = 000971-Y5   08:36 Friday, March 9, 2018

                                  The GLM Procedure

                          t Tests (LSD) for BulkDens5Depth

       NOTE: This test controls the Type I comparisonwise error rate, not the
                             experimentwise error rate.


                        Alpha                            0.05
                        Error Degrees of Freedom           63
                        Error Mean Square            0.001363
                        Critical Value of t           1.99834
                        Least Significant Difference   0.0218


             Means with the same letter are not significantly different.


                                                   Slope
                t Grouping          Mean      N    Position

                         A       1.10270     23    Mid
                         A
                         A       1.09513     23    Top
                         A
                         A       1.09161     23    Bottom

  Gilbert Pule: Experiment 2 data for statistical analysisNo Outliers 5cm only.SA 180
                             Project number = 000971-Y5   08:36 Friday, March 9, 2018

                                  The GLM Procedure

                           Dependent Variable: Na5Dpth

                                         Sum of
 Source                      DF         Squares     Mean Square    F Value    Pr > F

 Model                        5     179.0531742      35.8106348      18.70    <.0001

 Error                       62     118.7512729       1.9153431

 Corrected Total             67     297.8044471


                R-Square     Coeff Var      Root MSE    Na5Dpth Mean

                0.601244      61.67053      1.383959        2.244118


 Source                      DF       Type I SS     Mean Square    F Value    Pr > F

 Rainfall                     1     153.5177485     153.5177485      80.15    <.0001
 SlopePosition                2       9.1320750       4.5660375       2.38    0.1006
 Rainfall*SlopePositi         2      16.4033507       8.2016753       4.28    0.0181

  Gilbert Pule: Experiment 2 data for statistical analysisNo Outliers 5cm only.SA 181
                             Project number = 000971-Y5   08:36 Friday, March 9, 2018

                                  The GLM Procedure

                  Level of            -----------Na5Dpth-----------
                  Rainfall      N             Mean          Std Dev

                  593          33       3.79151515       1.71833881
                  654          35       0.78514286       1.21025853


               Level of                 -----------Na5Dpth-----------
               SlopePosition      N             Mean          Std Dev

               Bottom            24       2.78083333       2.73115029
               Mid               21       2.00523810       1.79183877
               Top               23       1.90217391       1.52596305


         Level of     Level of                 -----------Na5Dpth-----------
         Rainfall     SlopePosition      N             Mean          Std Dev

         593          Bottom            12       4.92750000       2.10710972
         593          Mid               10       3.33300000       1.10929457
         593          Top               11       2.96909091       0.97693863
         654          Bottom            12       0.63416667       1.04993037
         654          Mid               11       0.79818182       1.39579954
         654          Top               12       0.92416667       1.26795797

  Gilbert Pule: Experiment 2 data for statistical analysisNo Outliers 5cm only.SA 182
                             Project number = 000971-Y5   08:36 Friday, March 9, 2018

                                  The GLM Procedure

                              t Tests (LSD) for Na5Dpth

       NOTE: This test controls the Type I comparisonwise error rate, not the
                             experimentwise error rate.


                        Alpha                            0.05
                        Error Degrees of Freedom           62
                        Error Mean Square            1.915343
                        Critical Value of t           1.99897
                        Least Significant Difference   0.6713
                        Harmonic Mean of Cell Sizes  33.97059

                           NOTE: Cell sizes are not equal.


             Means with the same letter are not significantly different.


                t Grouping          Mean      N    Rainfall

                         A        3.7915     33    593

                         B        0.7851     35    654

  Gilbert Pule: Experiment 2 data for statistical analysisNo Outliers 5cm only.SA 183
                             Project number = 000971-Y5   08:36 Friday, March 9, 2018

                                  The GLM Procedure

                              t Tests (LSD) for Na5Dpth

       NOTE: This test controls the Type I comparisonwise error rate, not the
                             experimentwise error rate.


                        Alpha                            0.05
                        Error Degrees of Freedom           62
                        Error Mean Square            1.915343
                        Critical Value of t           1.99897
                        Least Significant Difference    0.823
                        Harmonic Mean of Cell Sizes  22.59649

                           NOTE: Cell sizes are not equal.


             Means with the same letter are not significantly different.


                                                      Slope
                   t Grouping          Mean      N    Position

                            A        2.7808     24    Bottom
                            A
                       B    A        2.0052     21    Mid
                       B
                       B             1.9022     23    Top

  Gilbert Pule: Experiment 2 data for statistical analysisNo Outliers 5cm only.SA 184
                             Project number = 000971-Y5   08:36 Friday, March 9, 2018

                                  The GLM Procedure

                           Dependent Variable: Ca5Depth

                                         Sum of
 Source                      DF         Squares     Mean Square    F Value    Pr > F

 Model                        5     296506.8404      59301.3681      21.16    <.0001

 Error                       65     182145.7799       2802.2428

 Corrected Total             70     478652.6202


                R-Square     Coeff Var      Root MSE    Ca5Depth Mean

                0.619461      73.75524      52.93621         71.77282


 Source                      DF       Type I SS     Mean Square    F Value    Pr > F

 Rainfall                     1     293393.9165     293393.9165     104.70    <.0001
 SlopePosition                2       2507.4531       1253.7266       0.45    0.6412
 Rainfall*SlopePositi         2        605.4708        302.7354       0.11    0.8978

  Gilbert Pule: Experiment 2 data for statistical analysisNo Outliers 5cm only.SA 185
                             Project number = 000971-Y5   08:36 Friday, March 9, 2018

                                  The GLM Procedure

                  Level of            -----------Ca5Depth----------
                  Rainfall      N             Mean          Std Dev

                  593          35       136.967714       68.7690144
                  654          36         8.388889       26.4395128


               Level of                 -----------Ca5Depth----------
               SlopePosition      N             Mean          Std Dev

               Bottom            24       66.1670833       78.7202839
               Mid               24       71.5570833       81.7784480
               Top               23       77.8473913       90.6298328


         Level of     Level of                 -----------Ca5Depth----------
         Rainfall     SlopePosition      N             Mean          Std Dev

         593          Bottom            12       126.503333       69.7334810
         593          Mid               12       138.388333       64.4999467
         593          Top               11       146.833636       76.8960696
         654          Bottom            12         5.830833       12.3098464
         654          Mid               12         4.725833        8.8460412
         654          Top               12        14.610000       43.9370167

  Gilbert Pule: Experiment 2 data for statistical analysisNo Outliers 5cm only.SA 186
                             Project number = 000971-Y5   08:36 Friday, March 9, 2018

                                  The GLM Procedure

                             t Tests (LSD) for Ca5Depth

       NOTE: This test controls the Type I comparisonwise error rate, not the
                             experimentwise error rate.


                        Alpha                            0.05
                        Error Degrees of Freedom           65
                        Error Mean Square            2802.243
                        Critical Value of t           1.99714
                        Least Significant Difference   25.096
                        Harmonic Mean of Cell Sizes  35.49296

                           NOTE: Cell sizes are not equal.


             Means with the same letter are not significantly different.


                t Grouping          Mean      N    Rainfall

                         A        136.97     35    593

                         B          8.39     36    654

  Gilbert Pule: Experiment 2 data for statistical analysisNo Outliers 5cm only.SA 187
                             Project number = 000971-Y5   08:36 Friday, March 9, 2018

                                  The GLM Procedure

                             t Tests (LSD) for Ca5Depth

       NOTE: This test controls the Type I comparisonwise error rate, not the
                             experimentwise error rate.


                        Alpha                            0.05
                        Error Degrees of Freedom           65
                        Error Mean Square            2802.243
                        Critical Value of t           1.99714
                        Least Significant Difference   30.739
                        Harmonic Mean of Cell Sizes  23.65714

                           NOTE: Cell sizes are not equal.


             Means with the same letter are not significantly different.


                                                   Slope
                t Grouping          Mean      N    Position

                         A         77.85     23    Top
                         A
                         A         71.56     24    Mid
                         A
                         A         66.17     24    Bottom

  Gilbert Pule: Experiment 2 data for statistical analysisNo Outliers 5cm only.SA 188
                             Project number = 000971-Y5   08:36 Friday, March 9, 2018

                                  The GLM Procedure

                           Dependent Variable: Mg5Depth

                                         Sum of
 Source                      DF         Squares     Mean Square    F Value    Pr > F

 Model                        5     20508.67550      4101.73510      11.64    <.0001

 Error                       64     22554.00868       352.40639

 Corrected Total             69     43062.68418


                R-Square     Coeff Var      Root MSE    Mg5Depth Mean

                0.476252      58.76214      18.77249         31.94657


 Source                      DF       Type I SS     Mean Square    F Value    Pr > F

 Rainfall                     1     19549.38724     19549.38724      55.47    <.0001
 SlopePosition                2       397.64445       198.82222       0.56    0.5716
 Rainfall*SlopePositi         2       561.64381       280.82190       0.80    0.4552

  Gilbert Pule: Experiment 2 data for statistical analysisNo Outliers 5cm only.SA 189
                             Project number = 000971-Y5   08:36 Friday, March 9, 2018

                                  The GLM Procedure

                  Level of            -----------Mg5Depth----------
                  Rainfall      N             Mean          Std Dev

                  593          34       49.1426471       24.8012395
                  654          36       15.7058333        9.5841358


               Level of                 -----------Mg5Depth----------
               SlopePosition      N             Mean          Std Dev

               Bottom            23       28.2995652       21.2495847
               Mid               24       33.8783333       26.1174344
               Top               23       33.5778261       27.7842745


         Level of     Level of                 -----------Mg5Depth----------
         Rainfall     SlopePosition      N             Mean          Std Dev

         593          Bottom            11       41.5509091       24.2142778
         593          Mid               12       51.9683333       24.8224121
         593          Top               11       53.6518182       25.8919110
         654          Bottom            12       16.1525000        5.7877663
         654          Mid               12       15.7883333        9.8032498
         654          Top               12       15.1766667       12.7332445

  Gilbert Pule: Experiment 2 data for statistical analysisNo Outliers 5cm only.SA 190
                             Project number = 000971-Y5   08:36 Friday, March 9, 2018

                                  The GLM Procedure

                             t Tests (LSD) for Mg5Depth

       NOTE: This test controls the Type I comparisonwise error rate, not the
                             experimentwise error rate.


                        Alpha                            0.05
                        Error Degrees of Freedom           64
                        Error Mean Square            352.4064
                        Critical Value of t           1.99773
                        Least Significant Difference   8.9684
                        Harmonic Mean of Cell Sizes  34.97143

                           NOTE: Cell sizes are not equal.


             Means with the same letter are not significantly different.


                t Grouping          Mean      N    Rainfall

                         A        49.143     34    593

                         B        15.706     36    654

  Gilbert Pule: Experiment 2 data for statistical analysisNo Outliers 5cm only.SA 191
                             Project number = 000971-Y5   08:36 Friday, March 9, 2018

                                  The GLM Procedure

                             t Tests (LSD) for Mg5Depth

       NOTE: This test controls the Type I comparisonwise error rate, not the
                             experimentwise error rate.


                        Alpha                            0.05
                        Error Degrees of Freedom           64
                        Error Mean Square            352.4064
                        Critical Value of t           1.99773
                        Least Significant Difference   10.982
                        Harmonic Mean of Cell Sizes  23.32394

                           NOTE: Cell sizes are not equal.


             Means with the same letter are not significantly different.


                                                   Slope
                t Grouping          Mean      N    Position

                         A        33.878     24    Mid
                         A
                         A        33.578     23    Top
                         A
                         A        28.300     23    Bottom

  Gilbert Pule: Experiment 2 data for statistical analysisNo Outliers 5cm only.SA 192
                             Project number = 000971-Y5   08:36 Friday, March 9, 2018

                                  The GLM Procedure

                         Dependent Variable: TotalN5Depth

                                         Sum of
 Source                      DF         Squares     Mean Square    F Value    Pr > F

 Model                        5      0.00330775      0.00066155       8.60    <.0001

 Error                       65      0.00499735      0.00007688

 Corrected Total             70      0.00830510


              R-Square     Coeff Var      Root MSE    TotalN5Depth Mean

              0.398279      14.83312      0.008768             0.059113


 Source                      DF       Type I SS     Mean Square    F Value    Pr > F

 Rainfall                     1      0.00280077      0.00280077      36.43    <.0001
 SlopePosition                2      0.00027088      0.00013544       1.76    0.1799
 Rainfall*SlopePositi         2      0.00023610      0.00011805       1.54    0.2231

  Gilbert Pule: Experiment 2 data for statistical analysisNo Outliers 5cm only.SA 193
                             Project number = 000971-Y5   08:36 Friday, March 9, 2018

                                  The GLM Procedure

                  Level of            ---------TotalN5Depth--------
                  Rainfall      N             Mean          Std Dev

                  593          36       0.06530556       0.01074861
                  654          35       0.05274286       0.00655449


               Level of                 ---------TotalN5Depth--------
               SlopePosition      N             Mean          Std Dev

               Bottom            24       0.06175000       0.00960638
               Mid               24       0.05745833       0.01184677
               Top               23       0.05808696       0.01107377


         Level of     Level of                 ---------TotalN5Depth--------
         Rainfall     SlopePosition      N             Mean          Std Dev

         593          Bottom            12       0.06550000       0.01069834
         593          Mid               12       0.06516667       0.01173056
         593          Top               12       0.06525000       0.01074604
         654          Bottom            12       0.05800000       0.00691507
         654          Mid               12       0.04975000       0.00511904
         654          Top               11       0.05027273       0.00374409

  Gilbert Pule: Experiment 2 data for statistical analysisNo Outliers 5cm only.SA 194
                             Project number = 000971-Y5   08:36 Friday, March 9, 2018

                                  The GLM Procedure

                           t Tests (LSD) for TotalN5Depth

       NOTE: This test controls the Type I comparisonwise error rate, not the
                             experimentwise error rate.


                        Alpha                            0.05
                        Error Degrees of Freedom           65
                        Error Mean Square            0.000077
                        Critical Value of t           1.99714
                        Least Significant Difference   0.0042
                        Harmonic Mean of Cell Sizes  35.49296

                           NOTE: Cell sizes are not equal.


             Means with the same letter are not significantly different.


                t Grouping          Mean      N    Rainfall

                         A      0.065306     36    593

                         B      0.052743     35    654

  Gilbert Pule: Experiment 2 data for statistical analysisNo Outliers 5cm only.SA 195
                             Project number = 000971-Y5   08:36 Friday, March 9, 2018

                                  The GLM Procedure

                           t Tests (LSD) for TotalN5Depth

       NOTE: This test controls the Type I comparisonwise error rate, not the
                             experimentwise error rate.


                        Alpha                            0.05
                        Error Degrees of Freedom           65
                        Error Mean Square            0.000077
                        Critical Value of t           1.99714
                        Least Significant Difference   0.0051
                        Harmonic Mean of Cell Sizes  23.65714

                           NOTE: Cell sizes are not equal.


             Means with the same letter are not significantly different.


                                                   Slope
                t Grouping          Mean      N    Position

                         A      0.061750     24    Bottom
                         A
                         A      0.058087     23    Top
                         A
                         A      0.057458     24    Mid

  Gilbert Pule: Experiment 2 data for statistical analysisNo Outliers 5cm only.SA 196
                             Project number = 000971-Y5   08:36 Friday, March 9, 2018

                                 The CORR Procedure

  17 With Variables:    Altitude        Longitude       Latitude        SplumDens
                        SplumMeanConCov IndivIntercep   BulkDens5Depth  K5Depth
                        Na5Dpth         Ca5Depth        Mg5Depth        Org_C5Depth
                        TotalN5Depth    SandPers5Depth  SiltPers5Depth  P5Depth
                        pHH2O5Depth
  17      Variables:    Altitude        Longitude       Latitude        SplumDens
                        SplumMeanConCov IndivIntercep   BulkDens5Depth  K5Depth
                        Na5Dpth         Ca5Depth        Mg5Depth        Org_C5Depth
                        TotalN5Depth    SandPers5Depth  SiltPers5Depth  P5Depth
                        pHH2O5Depth


                                  Simple Statistics

Variable                N        Mean     Std Dev         Sum     Minimum     Maximum

Altitude               69        1539    79.16159      106203        1447        1623
Longitude              70    25.93067     0.15774        1815    25.76853    26.09920
Latitude               70    28.05684     0.61138        1964    27.46497    28.68356
SplumDens              72    31.68056    22.09061        2281     2.00000   112.00000
SplumMeanConCov        72     1.23221     0.60464    88.71900           0     3.05000
IndivIntercep          72    11.30556     6.27905   814.00000           0    32.00000
BulkDens5Depth         69     1.09648     0.08900    75.65700     0.93700     1.22000
K5Depth                72    83.87417    47.25534        6039    21.82000   199.84000
Na5Dpth                68     2.24412     2.10828   152.60000           0     8.70000
Ca5Depth               71    71.77282    82.69156        5096           0   282.53000
Mg5Depth               70    31.94657    24.98193        2236     3.50000   104.42000
Org_C5Depth            72     1.14375     0.45496    82.35000     0.50000     2.37000
TotalN5Depth           71     0.05911     0.01089     4.19700     0.03900     0.08700
SandPers5Depth         72    74.83333    11.67035        5388    62.00000    88.00000
SiltPers5Depth         72     5.66667     3.16673   408.00000     2.00000    10.00000
P5Depth                72     2.63364     1.99008   189.62200     0.01000     7.84000
pHH2O5Depth            72     4.88389     0.21583   351.64000     4.42000     5.40000


                         Pearson Correlation Coefficients
                            Prob > |r| under H0: Rho=0
                               Number of Observations

                                                                   Splum
                                                        Splum    MeanCon      Indiv
                   Altitude   Longitude   Latitude       Dens        Cov   Intercep

 Altitude           1.00000     0.99814   -0.99817   -0.40129    0.26798   -0.22194
                                 <.0001     <.0001     0.0006     0.0260     0.0668
                         69          69         69         69         69         69

 Longitude          0.99814     1.00000   -0.99988   -0.40406    0.27097   -0.24162
                     <.0001                 <.0001     0.0005     0.0233     0.0439
                         69          70         70         70         70         70

 Latitude          -0.99817    -0.99988    1.00000    0.40229   -0.27034    0.23992
                     <.0001      <.0001                0.0006     0.0236     0.0454
                         69          70         70         70         70         70

 SplumDens         -0.40129    -0.40406    0.40229    1.00000   -0.60137    0.64235
                     0.0006      0.0005     0.0006                <.0001     <.0001
                         69          70         70         72         72         72

 SplumMeanConCov    0.26798     0.27097   -0.27034   -0.60137    1.00000   -0.29957
                     0.0260      0.0233     0.0236     <.0001                0.0106
                         69          70         70         72         72         72

 IndivIntercep     -0.22194    -0.24162    0.23992    0.64235   -0.29957    1.00000
                     0.0668      0.0439     0.0454     <.0001     0.0106
                         69          70         70         72         72         72

 BulkDens5Depth    -0.92607    -0.93051    0.93052    0.36065   -0.31454    0.16887
                     <.0001      <.0001     <.0001     0.0023     0.0085     0.1654
                         66          67         67         69         69         69

 K5Depth            0.67610     0.67320   -0.67239   -0.29116    0.14147   -0.14842
                     <.0001      <.0001     <.0001     0.0131     0.2359     0.2134
                         69          70         70         72         72         72

 Na5Dpth            0.71078     0.71767   -0.71680   -0.26005    0.12786   -0.20422
                     <.0001      <.0001     <.0001     0.0322     0.2988     0.0948
                         65          66         66         68         68         68

 Ca5Depth           0.80746     0.80693   -0.80690   -0.28084    0.24292   -0.17832
                     <.0001      <.0001     <.0001     0.0177     0.0412     0.1368
                         68          69         69         71         71         71

 Mg5Depth           0.69654     0.69343   -0.69347   -0.27557    0.23271   -0.18950
                     <.0001      <.0001     <.0001     0.0209     0.0525     0.1161
                         67          68         68         70         70         70

 Org_C5Depth        0.85883     0.85938   -0.86001   -0.27210    0.15134   -0.16952
                     <.0001      <.0001     <.0001     0.0208     0.2044     0.1546
                         69          70         70         72         72         72

 TotalN5Depth       0.57716     0.57842   -0.57880   -0.12782    0.03140   -0.06210
                     <.0001      <.0001     <.0001     0.2881     0.7949     0.6069
                         69          70         70         71         71         71

 SandPers5Depth    -0.98822    -0.99178    0.99220    0.41844   -0.30853    0.27325
                     <.0001      <.0001     <.0001     0.0003     0.0084     0.0202
                         69          70         70         72         72         72

 SiltPers5Depth     0.94344     0.95154   -0.95300   -0.31643    0.22664   -0.19881
                     <.0001      <.0001     <.0001     0.0068     0.0556     0.0941
                         69          70         70         72         72         72

 P5Depth            0.75622     0.75717   -0.75765   -0.35447    0.13338   -0.24635
                     <.0001      <.0001     <.0001     0.0023     0.2640     0.0370
                         69          70         70         72         72         72

 pHH2O5Depth       -0.02695    -0.04149    0.04025    0.17249   -0.14147    0.20458
                     0.8260      0.7331     0.7407     0.1474     0.2359     0.0847
                         69          70         70         72         72         72

                          Pearson Correlation Coefficients
                             Prob > |r| under H0: Rho=0
                               Number of Observations

                         Bulk                                                   Org_
                   Dens5Depth    K5Depth    Na5Dpth   Ca5Depth   Mg5Depth    C5Depth

 Altitude            -0.92607    0.67610    0.71078    0.80746    0.69654    0.85883
                       <.0001     <.0001     <.0001     <.0001     <.0001     <.0001
                           66         69         65         68         67         69

 Longitude           -0.93051    0.67320    0.71767    0.80693    0.69343    0.85938
                       <.0001     <.0001     <.0001     <.0001     <.0001     <.0001
                           67         70         66         69         68         70

 Latitude             0.93052   -0.67239   -0.71680   -0.80690   -0.69347   -0.86001
                       <.0001     <.0001     <.0001     <.0001     <.0001     <.0001
                           67         70         66         69         68         70

 SplumDens            0.36065   -0.29116   -0.26005   -0.28084   -0.27557   -0.27210
                       0.0023     0.0131     0.0322     0.0177     0.0209     0.0208
                           69         72         68         71         70         72

 SplumMeanConCov     -0.31454    0.14147    0.12786    0.24292    0.23271    0.15134
                       0.0085     0.2359     0.2988     0.0412     0.0525     0.2044
                           69         72         68         71         70         72

 IndivIntercep        0.16887   -0.14842   -0.20422   -0.17832   -0.18950   -0.16952
                       0.1654     0.2134     0.0948     0.1368     0.1161     0.1546
                           69         72         68         71         70         72

 BulkDens5Depth       1.00000   -0.77053   -0.64373   -0.84902   -0.77884   -0.89600
                                  <.0001     <.0001     <.0001     <.0001     <.0001
                           69         69         66         68         67         69

 K5Depth             -0.77053    1.00000    0.37916    0.84024    0.86124    0.82833
                       <.0001                0.0014     <.0001     <.0001     <.0001
                           69         72         68         71         70         72

 Na5Dpth             -0.64373    0.37916    1.00000    0.53429    0.42013    0.59187
                       <.0001     0.0014                <.0001     0.0004     <.0001
                           66         68         68         67         66         68

 Ca5Depth            -0.84902    0.84024    0.53429    1.00000    0.95436    0.92278
                       <.0001     <.0001     <.0001                <.0001     <.0001
                           68         71         67         71         70         71

 Mg5Depth            -0.77884    0.86124    0.42013    0.95436    1.00000    0.86751
                       <.0001     <.0001     0.0004     <.0001                <.0001
                           67         70         66         70         70         70

 Org_C5Depth         -0.89600    0.82833    0.59187    0.92278    0.86751    1.00000
                       <.0001     <.0001     <.0001     <.0001     <.0001
                           69         72         68         71         70         72

 TotalN5Depth        -0.71749    0.79436    0.40197    0.82106    0.82304    0.85946
                       <.0001     <.0001     0.0007     <.0001     <.0001     <.0001
                           68         71         67         70         69         71

 SandPers5Depth       0.91462   -0.65177   -0.71404   -0.76600   -0.67562   -0.81969
                       <.0001     <.0001     <.0001     <.0001     <.0001     <.0001
                           69         72         68         71         70         72

 SiltPers5Depth      -0.89300    0.64341    0.66518    0.74232    0.65924    0.80974
                       <.0001     <.0001     <.0001     <.0001     <.0001     <.0001
                           69         72         68         71         70         72

 P5Depth             -0.73797    0.67289    0.56408    0.72374    0.65497    0.80071
                       <.0001     <.0001     <.0001     <.0001     <.0001     <.0001
                           69         72         68         71         70         72

 pHH2O5Depth         -0.09794    0.53954   -0.13228    0.36580    0.46414    0.32624
                       0.4233     <.0001     0.2822     0.0017     <.0001     0.0052
                           69         72         68         71         70         72

                         Pearson Correlation Coefficients
                            Prob > |r| under H0: Rho=0
                               Number of Observations

                       Total          Sand          Silt                         p
                     N5Depth    Pers5Depth    Pers5Depth     P5Depth    HH2O5Depth

  Altitude           0.57716      -0.98822       0.94344     0.75622      -0.02695
                      <.0001        <.0001        <.0001      <.0001        0.8260
                          69            69            69          69            69

  Longitude          0.57842      -0.99178       0.95154     0.75717      -0.04149
                      <.0001        <.0001        <.0001      <.0001        0.7331
                          70            70            70          70            70

  Latitude          -0.57880       0.99220      -0.95300    -0.75765       0.04025
                      <.0001        <.0001        <.0001      <.0001        0.7407
                          70            70            70          70            70

  SplumDens         -0.12782       0.41844      -0.31643    -0.35447       0.17249
                      0.2881        0.0003        0.0068      0.0023        0.1474
                          71            72            72          72            72

  SplumMeanConCov    0.03140      -0.30853       0.22664     0.13338      -0.14147
                      0.7949        0.0084        0.0556      0.2640        0.2359
                          71            72            72          72            72

  IndivIntercep     -0.06210       0.27325      -0.19881    -0.24635       0.20458
                      0.6069        0.0202        0.0941      0.0370        0.0847
                          71            72            72          72            72

  BulkDens5Depth    -0.71749       0.91462      -0.89300    -0.73797      -0.09794
                      <.0001        <.0001        <.0001      <.0001        0.4233
                          68            69            69          69            69

  K5Depth            0.79436      -0.65177       0.64341     0.67289       0.53954
                      <.0001        <.0001        <.0001      <.0001        <.0001
                          71            72            72          72            72

  Na5Dpth            0.40197      -0.71404       0.66518     0.56408      -0.13228
                      0.0007        <.0001        <.0001      <.0001        0.2822
                          67            68            68          68            68

  Ca5Depth           0.82106      -0.76600       0.74232     0.72374       0.36580
                      <.0001        <.0001        <.0001      <.0001        0.0017
                          70            71            71          71            71

  Mg5Depth           0.82304      -0.67562       0.65924     0.65497       0.46414
                      <.0001        <.0001        <.0001      <.0001        <.0001
                          69            70            70          70            70

  Org_C5Depth        0.85946      -0.81969       0.80974     0.80071       0.32624
                      <.0001        <.0001        <.0001      <.0001        0.0052
                          71            72            72          72            72

  TotalN5Depth       1.00000      -0.56402       0.62295     0.64077       0.53366
                                    <.0001        <.0001      <.0001        <.0001
                          71            71            71          71            71

  SandPers5Depth    -0.56402       1.00000      -0.96191    -0.73503       0.08078
                      <.0001                      <.0001      <.0001        0.4999
                          71            72            72          72            72

  SiltPers5Depth     0.62295      -0.96191       1.00000     0.69783      -0.03641
                      <.0001        <.0001                    <.0001        0.7614
                          71            72            72          72            72

  P5Depth            0.64077      -0.73503       0.69783     1.00000       0.16661
                      <.0001        <.0001        <.0001                    0.1619
                          71            72            72          72            72

  pHH2O5Depth        0.53366       0.08078      -0.03641     0.16661       1.00000
                      <.0001        0.4999        0.7614      0.1619
                          71            72            72          72            72

  Gilbert Pule: Experiment 2 data for statistical analysisNo Outliers 5cm only.SA 197
                             Project number = 000971-Y5   08:36 Friday, March 9, 2018

Altitude*Longitude$GrassLandCommunity='*    Altitude*Latitude$GrassLandCommunity='*'

    1650 +                                      1650 +
         |                                           |
         |                                           |
         |                       * Gh15              |       * Gh15
         |                       * Gh15              |       * Gh15
    1600 +                       * Gh15         1600 +       * Gh15
         |                                           |
Altitude |                                  Altitude |
         |                                           |
         |                                           |
    1550 +                                      1550 +
         |                                           |
         |                                           |
         |                                           |
         |                                           |
    1500 +                                      1500 +
         |                                           |
         |                                           |
         |         * GM11                            |                        * GM11
         |         * GM11                            |                        * GM11
    1450 +         * GM11                       1450 +                        * GM11
         --+--------+--------+--------+-             -+-------------+-------------+-
         25.6     25.8     26.0     26.2             27            28            29

                    Longitude                                    Latitude

NOTE: 3 missing.  63 hidden.                NOTE: 3 missing.  63 hidden.


Altitude*SplumDens$GrassLandCommunity='*    Altitude*SplumMeanConCov$GrassLandCommun

    1650 +                                      1650 +
         |                                           |
         | Gh15 Gh15                                 |   Gh15 Gh15 Gh15 Gh15
         | G*1****     * Gh15                        | *  ***Gh1****   *** Gh15 Gh15
         | ******** Gh15                             |Gh15 Gh1***** ****   **     *
    1600 +Gh15* Gh15                            1600 +     Gh15Gh*5  Gh15
         |   Gh15                                    |          Gh15
Altitude |                                  Altitude |
         |                                           |
         |                                           |
    1550 +                                      1550 +
         |                                           |
         |                                           |
         |                                           |
         |                                           |
    1500 +                                      1500 +
         |                                           |
         | GM11 GM11                                 |            GM11   GM11 GM11
         |  *GM1*  GM11 GM11                         |GM11GM11 GM11* GM11 *    *
         | GM1***G**1** **GM11 * GM11                |G*11G*1*****G***M1* GM11
    1450 +GM11** *M*1**GM11** GM11              1450 + *GM*1******1**GM11
         --+--------+--------+--------+-             --+--------+--------+--------+-
           0       50       100      150               0        1        2        3

                    SplumDens                                SplumMeanConCov

NOTE: 66 gone.                              NOTE: 59 gone.


  Gilbert Pule: Experiment 2 data for statistical analysisNo Outliers 5cm only.SA 198
                             Project number = 000971-Y5   08:36 Friday, March 9, 2018

Altitude*IndivIntercep$GrassLandCommunit    Altitude*BulkDens5Depth$GrassLandCommuni

      1650 +                                    1650 +
           |                                         |
           |Gh15 Gh15 Gh15                           |       Gh15 Gh15
           |  * *G*1***** Gh15                       |    Gh15****** Gh15
           | Gh******* Gh15                          |    Gh1****** ** Gh15
      1600 +  Gh15 * Gh15                       1600 +     Gh15Gh1* Gh15
           |                                         |
  Altitude |                                Altitude |
           |                                         |
           |                                         |
      1550 +                                    1550 +
           |                                         |
           |                                         |
           |                                         |
           |                                         |
      1500 +                                    1500 +
           |                                         |
           |  GM11   GM11                            |
           |GM1* *GM1* GM11 GM11                     |             GM11 ** GM11
           |GM*1********G*11* * GM11                 |            GM11****GM11
      1450 +M1*G*M1*****GM1* GM11               1450 +           GGM1******M11
           ---+---------+---------+--                --+--------+--------+--------+-
              0        20        40                   0.8      1.0      1.2      1.4

                  IndivIntercep                               BulkDens5Depth

NOTE: 81 gone.                              NOTE: 73 gone.


Altitude*K5Depth$GrassLandCommunity='*'.    Altitude*Na5Dpth$GrassLandCommunity='*'.

    1650 +                                      1650 +
         |                                           |
         |         Gh15 Gh15 Gh15  Gh15              |   Gh15 Gh15 Gh15
         |  Gh15 * **Gh15** **  ** ***               |Gh15 ** *** *** Gh15 * Gh15
         |Gh15 *  *  ****  *** *Gh*5  *              | Gh15 *********Gh15     * Gh15
    1600 +     Gh15 *  Gh15 Gh15 Gh15Gh1        1600 +    Gh15 Gh15 *Gh15
         |                                           |             Gh15
Altitude |                                  Altitude |
         |                                           |
         |                                           |
    1550 +                                      1550 +
         |                                           |
         |                                           |
         |                                           |
         |                                           |
    1500 +                                      1500 +
         |                                           |
         |    GM11 GM11                              |GM11
         |GM11 *GM*1*GM11                            |*GMGGM11    * GM11
         | GM******G*GM11                            |******11 GM11
    1450 +GGM1*******M*1GM11     * GM11         1450 +**G*M*1GM*1  * GM11
         -+-------------+-------------+-             -+-------------+-------------+-
          0            100           200              0             5            10

                     K5Depth                                     Na5Dpth

NOTE: 64 gone.                              NOTE: 89 gone.


  Gilbert Pule: Experiment 2 data for statistical analysisNo Outliers 5cm only.SA 199
                             Project number = 000971-Y5   08:36 Friday, March 9, 2018

Altitude*Ca5Depth$GrassLandCommunity='*'    Altitude*Mg5Depth$GrassLandCommunity='*'

    1650 +                                      1650 +
         |                                           |
         |  Gh15 Gh15 Gh15 Gh15 Gh15                 | Gh15 Gh15 Gh15 Gh15
         | Gh15**  **** ***Gh*5* * *Gh15             |   * * *****  * *** Gh15
         |  Gh*5***** ***  **    *  *                | Gh1***** * **  * * * Gh15
    1600 +   Gh15 *Gh15Gh15Gh15 Gh15            1600 +  Gh15 *Gh15Gh15 Gh15
         |       Gh15                                |
Altitude |                                  Altitude |
         |                                           |
         |                                           |
    1550 +                                      1550 +
         |                                           |
         |                                           |
         |                                           |
         |                                           |
    1500 +                                      1500 +
         |                                           |
         |                                           |   GM11
         | * GM11                                    |GM1*** GM11
         |G**** GM11                                 |GM*** ** GM11
    1450 +G*1* GM11                             1450 +M1***G**GGM11
         --+--------+--------+--------+-             --+--------+--------+--------+-
           0       100      200      300               0       50       100      150

                     Ca5Depth                                    Mg5Depth

NOTE: 99 gone.                              NOTE: 84 gone.


Altitude*Org_C5Depth$GrassLandCommunity=    Altitude*TotalN5Depth$GrassLandCommunity

    1650 +                                      1650 +
         |                                           |
         |          Gh15Gh15GGh15                    |         Gh15 Gh15 Gh15
         |       Gh15G*******   * Gh15               |     Gh15 * ****** ****Gh15
         |      Gh15******* ** Gh15                  |   Gh15 ***  ****  ** ** Gh15
    1600 +         Gh15* Gh15                   1600 +          Gh15*Gh15 Gh15
         |                                           |             Gh15
Altitude |                                  Altitude |
         |                                           |
         |                                           |
    1550 +                                      1550 +
         |                                           |
         |                                           |
         |                                           |
         |                                           |
    1500 +                                      1500 +
         |                                           |
         |       GM11                                |          GM11
         |    GM1**GM11                              |       GM1** GM11
         | GM11 ***G*1GM11                           |   GM1GM*******1* GM11
    1450 + GGM11******M11                       1450 + GM1GG*M1******GG*11
         --+--------+--------+--------+-             --+--------+--------+--------+-
           0        1        2        3              0.025    0.050    0.075   0.100

                   Org_C5Depth                                 TotalN5Depth

NOTE: 76 gone.                              NOTE: 67 gone.


  Gilbert Pule: Experiment 2 data for statistical analysisNo Outliers 5cm only.SA 200
                             Project number = 000971-Y5   08:36 Friday, March 9, 2018

Altitude*SandPers5Depth$GrassLandCommuni    Altitude*SiltPers5Depth$GrassLandCommuni

    1650 +                                      1650 +
         |                                           |
         |Gh15                                       |
         | *   * Gh15                                |                     * Gh15 *
         | *   * Gh15                                |                     * Gh15 *
    1600 + *Gh15                                1600 +                       Gh15 *
         |Gh15                                       |
Altitude |                                  Altitude |
         |                                           |
         |                                           |
    1550 +                                      1550 +
         |                                           |
         |                                           |
         |                                           |
         |                                           |
    1500 +                                      1500 +
         |                                           |
         |                                           |
         |                       * GM11              |* GM11 * GM11
         |                  GM11 ** GM11             |* GM11 * GM11
    1450 +                   GM11**GM11         1450 +* GM11 * GM11
         --+--------+--------+--------+-             -+------+------+------+------+-
          62       72       82       92               2      4      6      8     10

                  SandPers5Depth                              SiltPers5Depth

NOTE: 3 missing.  59 hidden.                NOTE: 3 missing.  58 hidden.


Altitude*P5Depth$GrassLandCommunity='*'.    Altitude*pHH2O5Depth$GrassLandCommunity=

    1650 +                                      1650 +
         |                                           |
         |   Gh15 Gh15 Gh15 Gh15                     |           Gh15 Gh15 Gh15 Gh15
         |    * ** ****   ** *  * Gh15               |      Gh15 * * *** ** *** *
         | Gh15 * *******  ** Gh15                   |         *  *** *** * ** Gh15
    1600 +     Gh15G*15Gh15 Gh15                1600 +         Gh15*Gh15Gh15 Gh15
         |         Gh15                              |            Gh15
Altitude |                                  Altitude |
         |                                           |
         |                                           |
    1550 +                                      1550 +
         |                                           |
         |                                           |
         |                                           |
         |                                           |
    1500 +                                      1500 +
         |                                           |
         | GM11                                      |            GM11 GM11
         |*G**M11GM11                                |          GM1*GM1**  GM11
         |*******GM1* GM11                           |       GM11G***** *GM*1*GM11
    1450 +****G**GGM11* GM11                    1450 +        GM1GM** ****M11***GM11
         -+-------------+-------------+-             --+--------+--------+--------+-
          0             5            10               4.0      4.5      5.0      5.5

                     P5Depth                                   pHH2O5Depth

NOTE: 95 gone.                              NOTE: 56 gone.


  Gilbert Pule: Experiment 2 data for statistical analysisNo Outliers 5cm only.SA 201
                             Project number = 000971-Y5   08:36 Friday, March 9, 2018

Longitude*Altitude$GrassLandCommunity='*    Longitude*Latitude$GrassLandCommunity='*

     26.1 +                   * Gh15             26.1 +       * Gh15
          |             Gh15 *** Gh15                 |       * Gh15
          |                  Gh15                     |
          |                                           |
          |                                           |
     26.0 +                                      26.0 +
          |                                           |
Longitude |                                 Longitude |
          |                                           |
          |                                           |
     25.9 +                                      25.9 +
          |                                           |
          |                                           |
          |                                           |
          |                                           |
     25.8 +     GM11                             25.8 +
          |GM11 **                                    |                   GM11 *
          |    *** GM11                               |                   GM11 *
          |   GM11                                    |
          |                                           |
     25.7 +                                      25.7 +
          -+--------+--------+--------+-              -+-------------+-------------+
         1400     1500     1600     1700              27            28            29

                     Altitude                                    Latitude

NOTE: 3 missing.  60 hidden.                NOTE: 2 missing.  66 hidden.


Longitude*SplumDens$GrassLandCommunity='    Longitude*SplumMeanConCov$GrassLandCommu

     26.1 +Gh15 * Gh15                           26.1 +Gh15 Gh15 *  Gh15   Gh15 Gh15
          |********    * Gh15                         |*  *** ************ **     *
          |    Gh15                                   |         Gh15   Gh15
          |                                           |
          |                                           |
     26.0 +                                      26.0 +
          |                                           |
Longitude |                                 Longitude |
          |                                           |
          |                                           |
     25.9 +                                      25.9 +
          |                                           |
          |                                           |
          |                                           |
          |                                           |
     25.8 +  GM11 GM11                           25.8 +        GM11 GM11
          |GM1*** ** GM11  GM11                       |   GM11  ** **  GM11   GM11
          | * ****** ** ** **  * GM11                 |*  ******** ***  * *    *
          |GM11GM11GM11 GM11                          |GM11 GM11 GM11 GM11
          |                                           |
     25.7 +                                      25.7 +
          -+--------+--------+--------+-              -+--------+--------+--------+-
           0       50       100      150               0        1        2        3

                     SplumDens                                SplumMeanConCov

NOTE: 45 gone.                              NOTE: 2 missing.  31 hidden.


  Gilbert Pule: Experiment 2 data for statistical analysisNo Outliers 5cm only.SA 202
                             Project number = 000971-Y5   08:36 Friday, March 9, 2018

Longitude*IndivIntercep$GrassLandCommuni    Longitude*BulkDens5Depth$GrassLandCommun

       26.1 +   Gh15 * Gh15                      26.1 +     Gh15 * Gh15
            |  *********** Gh15                       | Gh15 ********* Gh15
            |      Gh15                               |         Gh15
            |                                         |
            |                                         |
       26.0 +                                    26.0 +
            |                                         |
  Longitude |                               Longitude |
            |                                         |
            |                                         |
       25.9 +                                    25.9 +
            |                                         |
            |                                         |
            |                                         |
            |                                         |
       25.8 +   GM11 GM11                        25.8 +                GM11
            |GM11*** *** GM11 GM11                    |           GM11 *** GM11
            |  ********** * ** * GM11                 |         GM11 ****** GM11
            |GM11 GM11 GM11                           |              GM11 GM11
            |                                         |
       25.7 +                                    25.7 +
            ---+---------+---------+--                -+--------+--------+--------+-
               0        20        40                  0.8      1.0      1.2      1.4

                   IndivIntercep                              BulkDens5Depth

NOTE: 2 missing.  38 hidden.                NOTE: 5 missing.  48 hidden.


Longitude*K5Depth$GrassLandCommunity='*'    Longitude*Na5Dpth$GrassLandCommunity='*'

     26.1 +      Gh15 Gh15 Gh15   * Gh15         26.1 +     Gh15   * Gh15     Gh15
          |Gh15 * ************* ** *****              |Gh15 ********** Gh15 *  *
          |         Gh15 Gh15   Gh15                  |      Gh15 Gh15
          |                                           |
          |                                           |
     26.0 +                                      26.0 +
          |                                           |
Longitude |                                 Longitude |
          |                                           |
          |                                           |
     25.9 +                                      25.9 +
          |                                           |
          |                                           |
          |                                           |
          |                                           |
     25.8 +    GM11                              25.8 +GM11 GM11
          | GM1***   GM11                             |**GM1* GM11 * GM11
          |   ********** GM11     * GM11              |******   *   * GM11
          |GM11  GM11 GM11                            |GM11 GM11
          |                                           |
     25.7 +                                      25.7 +
          -+-------------+-------------+              -+-------------+-------------+
           0            100          200               0             5            10

                      K5Depth                                     Na5Dpth

NOTE: 39 gone.                              NOTE: 49 gone.


  Gilbert Pule: Experiment 2 data for statistical analysisNo Outliers 5cm only.SA 203
                             Project number = 000971-Y5   08:36 Friday, March 9, 2018

Longitude*Ca5Depth$GrassLandCommunity='*    Longitude*Mg5Depth$GrassLandCommunity='*

     26.1 +   Gh15 Gh15 *  Gh15   Gh15           26.1 +   Gh15  * Gh15  Gh15
          |   **************** * * **                 |  ************ *** * Gh15
          |            Gh15   Gh15                    |        Gh15  Gh15
          |                                           |
          |                                           |
     26.0 +                                      26.0 +
          |                                           |
Longitude |                                 Longitude |
          |                                           |
          |                                           |
     25.9 +                                      25.9 +
          |                                           |
          |                                           |
          |                                           |
          |                                           |
     25.8 +GM11                                  25.8 +GM11
          |* GM11                                     | ** GM11
          |**** GM11                                  | ****** GM11
          |GM11                                       |GM11
          |                                           |
     25.7 +                                      25.7 +
          -+--------+--------+--------+-              -+--------+--------+--------+-
           0       100      200      300               0       50       100      150

                     Ca5Depth                                    Mg5Depth

NOTE: 3 missing.  43 hidden.                NOTE: 4 missing.  43 hidden.


Longitude*Org_C5Depth$GrassLandCommunity    Longitude*TotalN5Depth$GrassLandCommunit

     26.1 +       Gh15 *   Gh15                  26.1 +        Gh15  * Gh15  Gh15
          |    Gh15 **********  * Gh15                |  Gh15 *** ****** ***** Gh15
          |           Gh15                            |             Gh15  Gh15
          |                                           |
          |                                           |
     26.0 +                                      26.0 +
          |                                           |
Longitude |                                 Longitude |
          |                                           |
          |                                           |
     25.9 +                                      25.9 +
          |                                           |
          |                                           |
          |                                           |
          |                                           |
     25.8 +     GM11                             25.8 +      GM11 GM11
          |GM11 *** GM11                              |       ** ** GM11
          |GM11 ****** GM11                           |GM11 * ******* ** GM11
          |      GM11                                 |    GM11 GM11 GM11
          |                                           |
     25.7 +                                      25.7 +
          -+--------+--------+--------+-              -+--------+--------+--------+-
           0        1        2        3              0.025    0.050    0.075   0.100

                    Org_C5Depth                                TotalN5Depth

NOTE: 2 missing.  49 hidden.                NOTE: 2 missing.  41 hidden.


  Gilbert Pule: Experiment 2 data for statistical analysisNo Outliers 5cm only.SA 204
                             Project number = 000971-Y5   08:36 Friday, March 9, 2018

Longitude*SandPers5Depth$GrassLandCommun    Longitude*SiltPers5Depth$GrassLandCommun

     26.1 +    * Gh15                            26.1 +                     * Gh15
          |*   * Gh15                                 |                     * Gh15 *
          |Gh15                                       |
          |                                           |
          |                                           |
     26.0 +                                      26.0 +
          |                                           |
Longitude |                                 Longitude |
          |                                           |
          |                                           |
     25.9 +                                      25.9 +
          |                                           |
          |                                           |
          |                                           |
          |                                           |
     25.8 +                                      25.8 +
          |                 GM11 ** GM11              |* GM11
          |                 GM11 ** GM11              |* GM11 * GM11
          |                                           |
          |                                           |
     25.7 +                                      25.7 +
          -+--------+--------+--------+-              -+------+------+------+------+
          62       72       82       92                2      4      6      8     10

                  SandPers5Depth                              SiltPers5Depth

NOTE: 2 missing.  63 hidden.                NOTE: 2 missing.  64 hidden.


Longitude*P5Depth$GrassLandCommunity='*'    Longitude*pHH2O5Depth$GrassLandCommunity

     26.1 +  Gh15 *   Gh15  Gh15                 26.1 +           Gh15 *  Gh15 Gh15
          |    * ********* ****  * Gh15               |   Gh15 * ********** *** *
          |      Gh15                                 |               Gh15
          |                                           |
          |                                           |
     26.0 +                                      26.0 +
          |                                           |
Longitude |                                 Longitude |
          |                                           |
          |                                           |
     25.9 +                                      25.9 +
          |                                           |
          |                                           |
          |                                           |
          |                                           |
     25.8 +GM11 GM11                             25.8 +           GM11 GM11
          |  *****  GM11                              |            **** *  GM11 GM11
          |*******   * * GM11                         |      GM11 ** ******* ***
          |GM11 GM11                                  |           GM11 GM11 GM11
          |                                           |
     25.7 +                                      25.7 +
          -+-------------+-------------+              -+--------+--------+--------+-
           0             5            10              4.0      4.5      5.0      5.5

                      P5Depth                                   pHH2O5Depth

NOTE: 2 missing.  40 hidden.                NOTE: 2 missing.  37 hidden.


  Gilbert Pule: Experiment 2 data for statistical analysisNo Outliers 5cm only.SA 205
                             Project number = 000971-Y5   08:36 Friday, March 9, 2018

Latitude*Altitude$GrassLandCommunity='*'    Latitude*Longitude$GrassLandCommunity='*

    29.0 +                                      29.0 +
         |                                           |
         |     GM11                                  |
         |GM11 *** GM11                              |         * GM11
         |                                           |
    28.5 +                                      28.5 +
         |                                           |
Latitude |                                  Latitude |
         |                                           |
         |                                           |
    28.0 +                                      28.0 +
         |                                           |
         |                                           |
         |                                           |
         |                   Gh15                    |
    27.5 +              Gh15 *** Gh15           27.5 +                       * Gh15
         |                                           |
         |                                           |
         |                                           |
         |                                           |
    27.0 +                                      27.0 +
         --+--------+--------+--------+-             --+--------+--------+--------+-
         1400     1500     1600     1700             25.6     25.8     26.0     26.2

                     Altitude                                   Longitude

NOTE: 3 missing.  63 hidden.                NOTE: 2 missing.  68 hidden.


Latitude*SplumDens$GrassLandCommunity='*    Latitude*SplumMeanConCov$GrassLandCommun

    29.0 +                                      29.0 +
         |                                           |
         | GM11 GM11 GM11  GM11                      |   GM11  GM11 GM11 GM11 GM11
         |  * ****** ** ** **  * GM11                | *  ******** ***  * *    *
         |              GM11                         |GM11  GM11
    28.5 +                                      28.5 +
         |                                           |
Latitude |                                  Latitude |
         |                                           |
         |                                           |
    28.0 +                                      28.0 +
         |                                           |
         |                                           |
         |                                           |
         |Gh15  Gh15                                 |    Gh15 Gh15  Gh15  Gh15 Gh15
    27.5 + ********    * Gh15                   27.5 + *  *** ************ **     *
         |   Gh15                                    |Gh15        Gh15  Gh15
         |                                           |
         |                                           |
         |                                           |
    27.0 +                                      27.0 +
         --+--------+--------+--------+-             --+--------+--------+--------+-
           0       50       100      150               0        1        2        3

                    SplumDens                                SplumMeanConCov

NOTE: 2 missing.  47 hidden.                NOTE: 2 missing.  36 hidden.


  Gilbert Pule: Experiment 2 data for statistical analysisNo Outliers 5cm only.SA 206
                             Project number = 000971-Y5   08:36 Friday, March 9, 2018

Latitude*IndivIntercep$GrassLandCommunit    Latitude*BulkDens5Depth$GrassLandCommuni

      29.0 +                                    29.0 +
           |                                         |
           |  GM11 GM11 GM11 GM11                    |             GM11 GM11
           |  ********** * ** * GM11                 |          GM11 ****** GM11
           |                                         |
      28.5 +                                    28.5 +
           |                                         |
  Latitude |                                Latitude |
           |                                         |
           |                                         |
      28.0 +                                    28.0 +
           |                                         |
           |                                         |
           |                                         |
           |  Gh15  Gh15                             |       Gh15  Gh15
      27.5 +  *********** Gh15                  27.5 +  Gh15 ********* Gh15
           |     Gh15                                |          Gh15
           |                                         |
           |                                         |
           |                                         |
      27.0 +                                    27.0 +
           ---+---------+---------+--                --+--------+--------+--------+-
              0        20        40                   0.8      1.0      1.2      1.4

                  IndivIntercep                               BulkDens5Depth

NOTE: 2 missing.  45 hidden.                NOTE: 5 missing.  52 hidden.


Latitude*K5Depth$GrassLandCommunity='*'.    Latitude*Na5Dpth$GrassLandCommunity='*'.

    29.0 +                                      29.0 +
         |                                           |
         |  GM11 GM11 GM11                           |  GM11  GM11 GM11
         |   ********** GM11     * GM11              |******   *  ** GM11
         |                                           |GM11
    28.5 +                                      28.5 +
         |                                           |
Latitude |                                  Latitude |
         |                                           |
         |                                           |
    28.0 +                                      28.0 +
         |                                           |
         |                                           |
         |                                           |
         |      Gh15     Gh15 Gh15  Gh15             |     Gh15  Gh15
    27.5 +Gh15 * ************* ********         27.5 +Gh15 ********** Gh15 *  * Gh15
         |         Gh15 Gh15     Gh15                |        Gh15
         |                                           |
         |                                           |
         |                                           |
    27.0 +                                      27.0 +
         -+-------------+-------------+-             -+-------------+-------------+-
          0            100           200              0             5            10

                     K5Depth                                     Na5Dpth

NOTE: 2 missing.  37 hidden.                NOTE: 6 missing.  45 hidden.


  Gilbert Pule: Experiment 2 data for statistical analysisNo Outliers 5cm only.SA 207
                             Project number = 000971-Y5   08:36 Friday, March 9, 2018

Latitude*Ca5Depth$GrassLandCommunity='*'    Latitude*Mg5Depth$GrassLandCommunity='*'

    29.0 +                                      29.0 +
         |                                           |
         | GM11                                      |  GM11 GM11
         | **** GM11                                 |  ****** GM11
         |                                           |
    28.5 +                                      28.5 +
         |                                           |
Latitude |                                  Latitude |
         |                                           |
         |                                           |
    28.0 +                                      28.0 +
         |                                           |
         |                                           |
         |                                           |
         |    Gh15 Gh15 Gh15  Gh15 Gh15              | Gh15 Gh15 Gh15  Gh15
    27.5 +    **************** * * **           27.5 +   ************ *** * Gh15
         |                 Gh15                      |
         |                                           |
         |                                           |
         |                                           |
    27.0 +                                      27.0 +
         --+--------+--------+--------+-             --+--------+--------+--------+-
           0       100      200      300               0       50       100      150

                     Ca5Depth                                    Mg5Depth

NOTE: 3 missing.  45 hidden.                NOTE: 4 missing.  46 hidden.


Latitude*Org_C5Depth$GrassLandCommunity=    Latitude*TotalN5Depth$GrassLandCommunity

    29.0 +                                      29.0 +
         |                                           |
         |      GM11 GM11                            |     GM11  GM11 GM11
         | GM11 ****** GM11                          |      * ******* ** GM11
         |                                           |        GM11
    28.5 +                                      28.5 +
         |                                           |
Latitude |                                  Latitude |
         |                                           |
         |                                           |
    28.0 +                                      28.0 +
         |                                           |
         |                                           |
         |                                           |
         |        Gh15 Gh15 Gh15                     |        Gh15 Gh15  Gh15
    27.5 +     Gh15 **********  * Gh15          27.5 +   Gh15 *** ****** ***** Gh15
         |                                           |                Gh15
         |                                           |
         |                                           |
         |                                           |
    27.0 +                                      27.0 +
         --+--------+--------+--------+-             --+--------+--------+--------+-
           0        1        2        3              0.025    0.050    0.075   0.100

                   Org_C5Depth                                 TotalN5Depth

NOTE: 2 missing.  53 hidden.                NOTE: 2 missing.  46 hidden.


  Gilbert Pule: Experiment 2 data for statistical analysisNo Outliers 5cm only.SA 208
                             Project number = 000971-Y5   08:36 Friday, March 9, 2018

Latitude*SandPers5Depth$GrassLandCommuni    Latitude*SiltPers5Depth$GrassLandCommuni

    29.0 +                                      29.0 +
         |                                           |
         |                                           |
         |                  GM11 ** GM11             |* GM11 * GM11
         |                                           |
    28.5 +                                      28.5 +
         |                                           |
Latitude |                                  Latitude |
         |                                           |
         |                                           |
    28.0 +                                      28.0 +
         |                                           |
         |                                           |
         |                                           |
         |Gh15                                       |
    27.5 + *   * Gh15                           27.5 +                     * Gh15 *
         |                                           |
         |                                           |
         |                                           |
         |                                           |
    27.0 +                                      27.0 +
         --+--------+--------+--------+-             -+------+------+------+------+-
          62       72       82       92               2      4      6      8     10

                  SandPers5Depth                              SiltPers5Depth

NOTE: 2 missing.  66 hidden.                NOTE: 2 missing.  66 hidden.


Latitude*P5Depth$GrassLandCommunity='*'.    Latitude*pHH2O5Depth$GrassLandCommunity=

    29.0 +                                      29.0 +
         |                                           |
[truncated: 770,060 more chars]
